# Annotated checklist of the terrestrial molluscs from Laos (Mollusca, Gastropoda)

**DOI:** 10.3897/zookeys.834.28800

**Published:** 2019-04-03

**Authors:** Khamla Inkhavilay, Chirasak Sutcharit, Ueangfa Bantaowong, Ratmanee Chanabun, Warut Siriwut, Ruttapon Srisonchai, Arthit Pholyotha, Parin Jirapatrasilp, Somsak Panha

**Affiliations:** 1 Department of Biology, Faculty of Natural Science, National University of Laos, P.O. Box 7322, Dongdok, Vientiane, Laos National University of Laos Vientiane Laos; 2 Animal Systematics Research Unit, Department of Biology, Faculty of Science, Chulalongkorn University, Bangkok 10330, Thailand Chulalongkorn University Bangkok Thailand; 3 Division of Biology, Faculty of Science and Technology, Rajamangala University of Technology Thanyaburi, Pathumthani 12110, Thailand University of Technology Thanyaburi Pathumthani Thailand; 4 Program in Animal Science, Faculty of Agriculture Technology, Sakon Nakhon Rajabhat University, Sakon Nakhon 47000, Thailand Sakon Nakhon Rajabhat University Sakon Nakhon Thailand; 5 Department of Biology, Faculty of Science, Mahidol University, Bangkok, 10400, Thailand Mahidol University Bangkok Thailand

**Keywords:** Biodiversity, conservation, land snail, type specimen, Southeast Asia, new name, Indochina

## Abstract

The land area of Laos is composed of a large variety of undisturbed habitats, such as high mountainous areas, huge limestone karsts and the lower Mekong Basin. Therefore, Laos is expected to have a high species diversity, especially for the land snails. However, with respect to research on malacology, Laos is probably the least well-researched area for land snail diversity in Indochina (including Laos) over the past few centuries. The handful of species lists have never been systematically revised from the colonial period to the present, so these classifications are outdated. Herein we present the first comprehensive annotated checklist with an up-to-date systematic framework of the land snail fauna in Laos based on both field investigations and literature surveys. This annotated checklist is collectively composed of 231 nominal species (62 ‘prosobranch’ and 169 heterobranches), of which 221 nominal species are illustrated. The type specimens of 143 species from several museum collections and/or 144 species of newly collected specimens are illustrated. There are 58 species recorded as new to the malacofauna of the country, and two new replacement names are proposed as *Hemiplectalanxangnica* Inkhavilay and Panha, **nomen novum** (Ariophantidae) and *Chloritiskhammouanensis* Inkhavilay and Panha, **nomen novum** (Camaenidae). Four recently described species of the genus *Amphidromus* from Laos, “*thakhekensis*”, “*richgoldbergi*”, “*attapeuensis*” and “*phuonglinhae*” are synonymized with previously described species. In addition, thirteen nominal species are listed as uncertain records that may or may not occur in Laos. This annotated checklist may inspire malacologists to carry on systematic research in this region.

## Introduction

Laos, or Lao PDR, is located in the center of the Indo-Burma region, one of the global biodiversity hotspots (Myers et al. 2000). The geographical subdivisions of the country comprise the mountainous and dry-evergreen forests in the north, massive limestone karsts, the Annamite ranges and deciduous forests in the central region, and sandstones with the Mekong floodplain and dry-dipterocarp forests in the south (King et al. 1975). These complex geographical areas associated with different climatic and vegetation conditions provide highly diverse habitats, many of which are still undisturbed, allowing the notion that Laos likely has numerous faunal and floral ‘treasures’ (Mittermeier et al. 2004, Prosperi et al. 2018). Several vertebrate faunal inventories have been performed and reported (see Duckworth et al. 1999). However, various groups of invertebrates well known as important bio-indicators for all types of forest (Hodkinson and Jackson 2005), especially the land snails are still poorly investigated.

The basic information on land snail biodiversity in Laos is scarce and scantly known. So far, the most likely first record dates back to around the middle of the nineteenth century. The well-known naturalist and traveller, Henri Mouhot, had explored the Indochina region especially during his fourth mission in Siam where he journeyed from Bangkok to Luang Phrabang (previously under the rule of Siam [Thailand]). The land snail collections gathered during Mouhot’s travel were then sent back to England under the Hugh Cuming legacy (Dance 1980, 1986) and were primarily examined and described by L. Pfeiffer (1860a, 1861a, 1863a). However, the localities recorded by H. Mouhot were very rough and only at a broad scale, such as “Camboja”, “Siam” and “Lao Mountains” (Mouhot 1864a, b). This has made the distribution range for several species to be far from certain.

Later in the late nineteenth century, the Siamese territory on the east bank of the Mekong River (forming the present-day Laos) was appended to the French colonial territories as French Indochina. Around this period, the most important explorer was the French civil servant and diplomat Auguste Pavie (his surveys so-called “Mission Pavie”), who dealed with not only diplomacy and politics but also geology and natural history (Pavie 1904). His collected natural objects included land snail specimens from French Indochina [Cambodia, Laos, Vietnam and a part of Thailand] which were subsequently examined and described by several malacologists such as C.-F. Ancey, A. Bavay, H. Crosse, P. Dautzenberg, P. Fischer and L. Morlet. There were fifty nominal species of land snails from so called “Laos” which were collected during the Mission Pavie (Fischer 1891, Fischer and Dautzenberg 1904), and these were the only species lists of land snails from this area. Later, the land snail collections by the French geologist H. Counillon, mainly from the Luang Phrabang area were examined and these included some 23 nominal species (Ancey 1898). In addition, land snail collections by the German orchid collector Carl Roebelen from “Boloven” which included 13 new species were studied and published by Möllendorff (1898). About 50 years later, the French geologist, Edmund Saurin (1953) examined land snails from Pa Hia, Tran Ninh Province [probably refers to Ban Namthong, Longchaeng District, Xaisomboun Province, Laos; see Páll-Gergely et al. (2016: 13)] and described nine new species, which brought the diversity of land snails in Laos to 64 nominal species. Another fifty years later, Panha et al. (2002), Lehmann and Maassen (2004) and Maassen (2008) studied a range of land snails from various parts of Laos, recording five new taxa. The most recent treatments on Laotian land snails are from Inkhavilay et al. (2016a, b, 2017) and Páll-Gergely et al. (2016, 2017b).

This paper is the first comprehensive treatment to update the land snail diversity from Laos since the “Mission Pavie” by Fischer and Dautzenberg (1904). This study focuses on the list of the species that were formerly recorded in the literature and additionally collected from a two-year (2013–2014) field survey throughout the country, which yielded a number of new records. This includes the taxonomic updates, illustrations of type specimens (when possible) and photos of newly collected specimens. Although many land snail groups have been paid little attention and never been subjected to modern systematic revision, we also attempt to classify and clarify the vague taxa in a modern systematic framework. We hope that this article will provide the fundamental and overall knowledge on land snail biodiversity in Laos and inspire local biologists who are interested in the country’s land snail heritages.

## Materials and methods

### Sources

**Figure 1.**
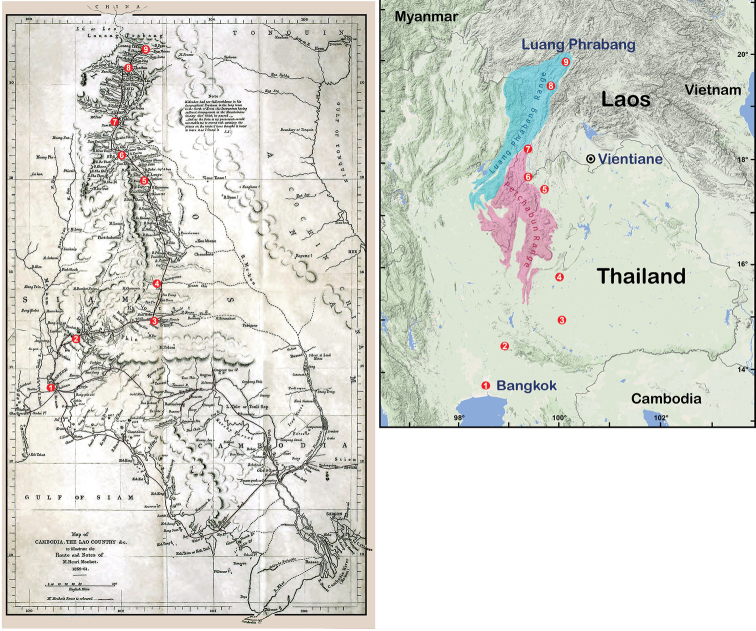
Henri Mouhot travel routes map (after Mouhot 1864b) and the present-day geographical map of the 4^th^ trip from Bangkok to Luang Phrabang during 1860–1861. The numbers indicating the important cities or towns are depicted on the Mouhot map. The numbers 1 to 6 are located in present-day Thailand and 7 to 9 are in Laos. The present use of location names is given in the square brackets. **1** Bangkok **2** Patawi [Wat Phra Phutthachai, Saraburi Province] **3** Korat [Nakhon Ratchasima Province] **4** Chaiapume [Chaiyaphum Province] **5** Leuye [Loei Province] **6** Kenne Tao [Kenethao District, Xayaboury Province] **7** Paklaie [Parklai District, Xayaboury Province] **8** Thadua [Tha Deua village, Xayaboury District, Xayaboury Province] and **9** Luang Phrabang.

The data compiled in the checklist of land snails from Laos are from two main sources. The first is from the published malacological literature ranging from the nineteenth century until 2017. This historical literature, including the “*Journal de Conchyliologie*”, is available online at www.biodiversitylibrary.org and www.archive.org. The taxa described in Thach (2018) were not taken into account in this study. Furthermore, this list includes all taxa that have the type locality or subsequent reports as recorded in the areas forming the present-day “Laos”. The taxa with the type locality of “Lao Mountains, Camboja” which were described by L. Pfeiffer based on specimens from the H. Cuming ex. H. Mouhot collection are also included. This uncertain and broad-scale geographical area more likely refers to the mountainous areas of the Luang Phrabang Ranges and Petchabun Ranges in Laos and Thailand, respectively (Fig. 1). There are many taxa described by Pfeiffer (1860a, 1861a, 1863a) that were found in several localities in Laos and Thailand, but not in present-day Cambodia. Moreover, two species, *Moellendorffiahorrida* (Pfeiffer, 1863) and *Naggsialaomontana* (Pfeiffer, 1863), seem to be endemic to limestone karsts in present-day Laos. Although Fischer (1973a, b) listed *Camaenaillustris* (Pfeiffer, 1863) and *Haploptychiuspellucens* (Pfeiffer, 1863) as being found in Cambodia, the distribution of these species has to be verified with newly collected specimens and precise location records.

**Figure 2.**
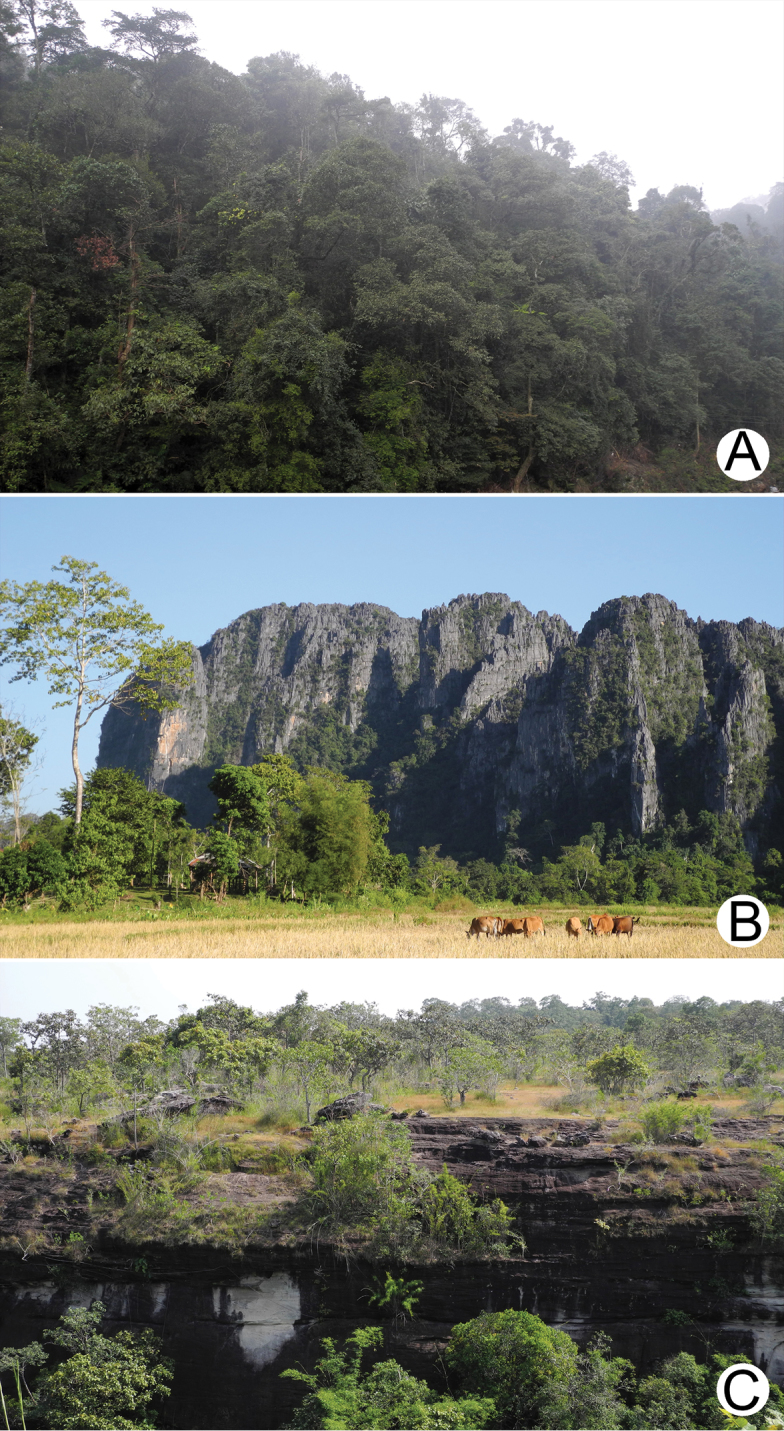
Three main habitat types following geographical subdivisions, north, central and south of Laos. **A** Natural vegetation type of mountainous and dry-evergreen forests in the north, Luang Phrabang Province **B** Massive limestone karsts and caves in the central, Vientiane Province **C** Sandstones and dry-dipterocarp forests in lowlands of the south, Champasak Province.

The other source of information was from field surveys performed during the years 2013–2014. Land snails in Laos were sampled using direct search techniques throughout the country, including the north mountainous forests, limestone areas in the central area and deciduous forests in the south (Fig. 2). Moreover, surveys were conducted in both the National Protected Areas (NPA) with permission (NUL-2013-2014), and non-protected areas including anthropogenic and plantation areas. The direct searching involved all potential land snail microhabitats that could be accessed, such as deep litter beds, decaying tree trunks, rock surfaces and crevices and, especially, limestone cliffs and caves.

All sampled locations were recorded. At each locality, we searched intensively for land snails for about 1 to 2 hours by 3 to 4 well-trained assistants. All living snails and slugs were photographed before being preserved in 70% ethanol, and empty shells were air dried in mesh-bags for one to two weeks before being sorted.

### Structure of the list

Species determination and identification of specimens are based on the literature and comparisons with the type specimens and/or reference collections from several natural history museums. The classification of the higher taxa in the list is according to Bouchet et al. (2017) and the generic placements follow Kobelt (1902a), Wenz (1938), Zilch (1959–1960) and Vaught (1989). Within each family, genera and species are listed alphabetically. Within each species or subspecies, the treatment includes the original combination of the taxon name with original spelling, references to the page(s) and plate and/or figures. The type locality is given verbatim as stated in the original publication. However, when possible the modern name and/or regional name of the type locality is provided in square brackets. The most recent usage of the locality name and distribution records that addresses the occurrences of that particular taxon in “Laos” is also provided. When possible, the type materials with catalogue numbers, the illustrations of the type specimens and/or newly collected specimens are also provided. For taxa that could not be assigned to an existing name, a provisional taxonomic name is given (for example, *Trochomorpha* (?) sp.). The taxa that have long been known only from the literature, where the type specimens could not be traced and no new specimen was found during the field survey were also included, where their distribution records were based on the literature.

### Institutional Abbreviations

**AMNH** American Museum of Natural History, New York

**ANSP** The Academy of Natural Sciences of Drexel University, Philadelphia

**CUMZ** Chulalongkorn University Museum of Zoology, Bangkok

**FMNH** Field Museum of Natural History, Chicago

**HNHM** Hungarian Natural History Museum, Budapest

**MNHN** Muséum National ďHistoire Naturelle, Paris

**NHMUK** Natural History Museum, London

**NMW** National Museum of Wales, Cardiff

**RBINS** Royal Belgian Institute of Natural Sciences, Brussels

**RMNH** Naturalis Biodiversity Center, Rijksmuseum van Natuurlijke Historie, Leiden

**SMF** Forschungsinstitut und Naturmuseum Senckenberg, Frankfurt am Main

**ZMB** Museum of Natural History (Museum für Naturkunde), Berlin

### Photo credits

Photos of the type specimens from the Molluscs Collection (IM) of MNHN are credited to the museum taken under the project E-RECOLNAT: ANR-11-INBS-0004, or stated otherwise. Photos of the type specimens from FMNH and HNHM are credited to Jochen Gerber and Barna Páll-Gergely, respectively. Photos of the type specimens from the other museum collections are credited to each respective museum.

## Results

This annotated checklist is based on the published literature up to 2017 and field surveys from 2013 to 2014. It comprises 231 nominal species of land snail fauna in Laos. There are 24 genera and 62 species of the ‘prosobranchs’, and 57 genera and 169 species and nine subspecies of the heterobranchs. Among these, 221 nominal species are accompanied with figures, 67 of which are known only from the type specimens. The list also includes 11 unidentified species that were collected during our field surveys and five species known only from the literature without newly collected specimens and without indication of the type specimens. In addition, thirteen taxa are considered as uncertain records requiring verification through further surveys.

### Systematic lists

#### Class Gastropoda

##### Subclass Neritimorpha

###### Order Cycloneritida

####### Superfamily Helicinoidea

######## Family Helicinidae Férussac, 1822

######### *Aphanoconia* Wagner, 1905

########## Aphanoconia hungerfordiana

Taxon classificationAnimaliaCycloneritidaHelicinidae

(Möllendorff, 1882)

Helicina hungerfordiana Möllendorff, 1882a: 182. Type locality: Hong Kong; Tung-dshou, Macao [Hong Kong and Macau, China]. Möllendorff 1882b: 354.
Aphanoconia hungerfordiana : Wagner 1905: 389, pl. 4, figs 10a–c. Wagner 1907: 190, 191, pl. 38, figs 1–5.

########### Material examined.

**Figure 3.**
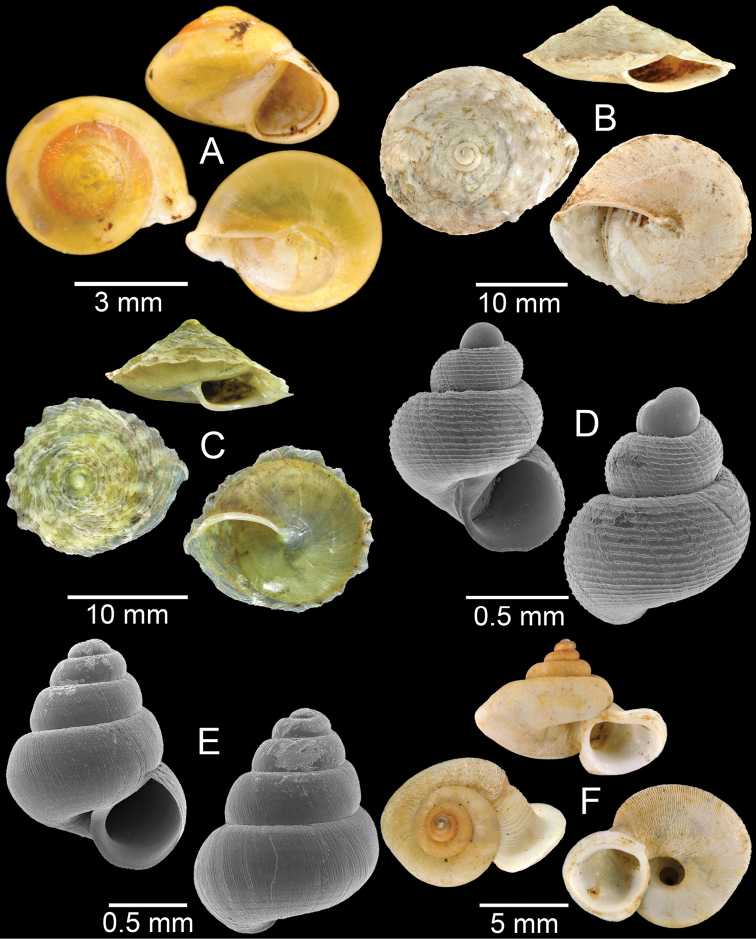
**A***Aphanoconiahungerfordiana*, CUMZ collection **B***Calybiummassiei*, CUMZ collection **C***Geotrochatellamouhoti*, CUMZ collection **D***Georissadecora*, CUMZ collection **E***Acmella* sp., CUMZ collection **F***Alycaeusrolfbrandti*, CUMZ collection.

Specimens from km 30, Laos-Vietnam border road, Yommalath District, Khammouan Province (Fig. 3A).

########### Distribution.

Hong Kong, China and Vietnam (Wagner 1907).

########### Remarks.

Two of the three subspecies, *Aphanoconiahungerfordianahalongensis* Wagner, 1905 and *Aphanoconiahungerfordianatonkinensis* Möllendorff in Wagner, 1907, were reported from Northern Vietnam. However, in this survey, only one shell was collected, so the status of the subspecific taxa could not be confidently determined.

######### *Calybium* Morlet, 1892

########## Calybium massiei

Taxon classificationAnimaliaCycloneritidaHelicinidae

Morlet, 1892

Calybium massiei Morlet, 1892a[1891]: 316, 317. Type locality: propè Kham-Keut in Provinciâ Laos dicta [around Khamkeut District, Bolikhamxay Province, Laos]. Morlet 1893[1892]: 327, pl. 8, figs 2, 2a–d.
Calybium masiei [sic]: Wagner 1907: 15, 16, pl. 2, figs 8–11.

########### Material examined.

Specimens from Tam Mungkorn Cave, Khamkeut District, Bolikhamxay Province (Fig. 3B).

########### Distribution.

Laos (Wagner 1907).

######### *Geotrochatella* Fischer, 1891

########## Geotrochatella mouhoti

Taxon classificationAnimaliaCycloneritidaHelicinidae

(Pfeiffer, 1863)

Trochatella mouhoti Pfeiffer, 1863a[1862]: 277, pl. 36, fig. 14. Type locality: Lao Mountains, Camboja [Cambodia or Laos]. Pfeiffer 1865: 254, 255, pl. 64, figs 9–11.
Geotrochatella mouhoti : Wagner 1907: 11, 12, pl. 1, figs 5, 6, 20, 21.

########### Material examined.

Specimens from limestone near Tam Tarn Kaison Cave, Viengxay District, Houaphanh Province (Fig. 3C).

########### Distribution.

Cambodia, Laos and Thailand (Wagner 1907).

####### Superfamily Hydrocenoidea

######## Family Hydrocenidae Troschel, 1857

######### *Georissa* Blanford, 1864

########## Georissa decora

Taxon classificationAnimaliaCycloneritidaHydrocenidae

Möllendorff, 1900

Georissa decora Möllendorff, 1900: 138. Type locality: Touranne [Danang Province, Vietnam]. Zilch 1973b: 264, pl. 12, fig. 3. Do et al. 2015: 131, fig. 7f.

########### Material examined.

Specimens from Tam Xang Cave, Thakhek District, Khammouan Province (Fig. 3D).

########### Distribution.

Vietnam (Zilch 1973b, Do et al. 2015).

##### Subclass Caenogastropoda

###### Cohort Sorbeoconcha

####### Subcohort Hypsogastropoda

######## Superfamily Truncatelloidea

######### Family Assimineidae H. Adams & A. Adams, 1856

########## *Acmella* Blanford, 1869

########### Acmella

Taxon classificationAnimaliaCycloneritidaAssimineidae

sp.

############ Material examined.

Specimens from Tam Mungkorn Cave, Khamkeut District, Bolikhamxay Province (Fig. 3E).

############ Remarks.

These specimens are small, rather thick and hardly translucent. The species differs from *Georissadecora* in having a dull, relatively smooth shell surface with thin and inconspicuous growth lines only, and spiral sculpture absent, while the former species has very strong and prominent spiral ridges.

####### Grade Architaenioglossa

######## Superfamily Cyclophoroidea

######### Family Cyclophoridae Gray, 1847

########## Subfamily Alycaeinae Blanford, 1864

########### *Alycaeus* Gray, 1850

############ Alycaeus mouhoti

Taxon classificationAnimaliaCycloneritidaCyclophoridae

Pfeiffer, 1863

Alycaeus mouhoti Pfeiffer, 1863a[1862]: 275, pl. 36, figs 1, 2. Type locality: Lao Mountains, Camboja [Cambodia or Laos]. Pfeifer 1863b: 228, 229, pl. 59, figs 9–11. Kobelt 1902a: 347. Saurin 1953: 113.

############# Material examined.

**Figure 4.**
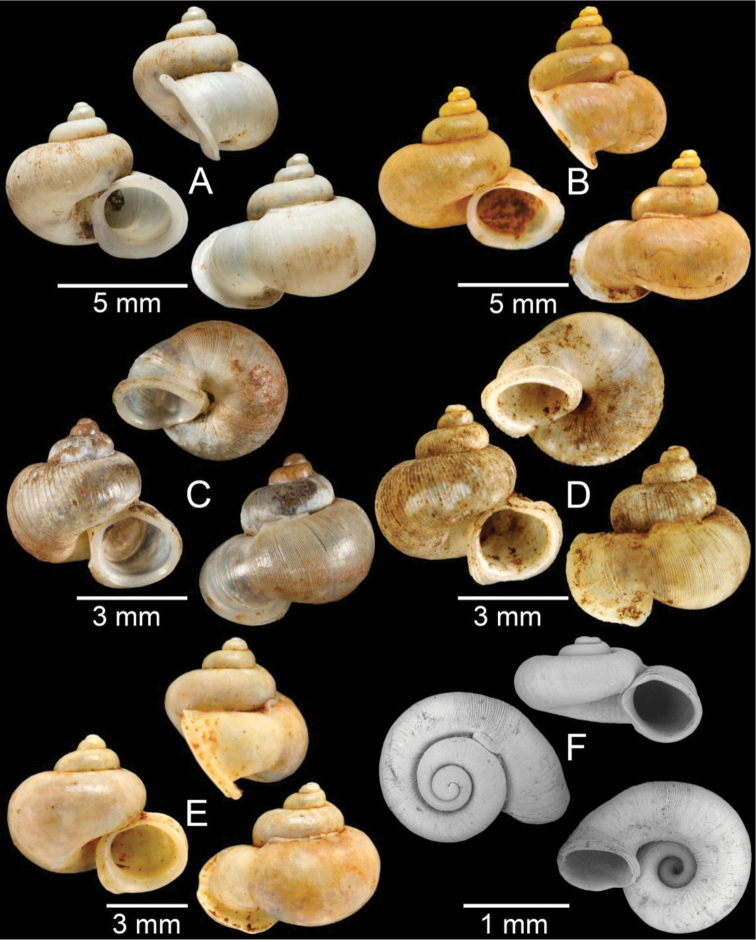
**A, B***Alycaeusmouhoti***A** syntype NHMUK 20170120 and **B** CUMZ collection **C–E***Alycaeusvanbuensis***C** syntype MNHN-IM-2000-31798 and **D, E** CUMZ collection **F***Dicharaxabdoui*, holotype MNHN-IM-2012-27329. Photo: B. Páll-Gergely (**F**).

Syntypes NHMUK 20170120 from “Lao Mountains” (3 shells; Fig. 4A). Specimens from Tam Phatok Cave, Ngoy District, Luang Phrabang Province (Fig. 4B).

############# Distribution.

Laos (Kobelt 1902a, Saurin 1953).

############ Alycaeus rolfbrandti

Taxon classificationAnimaliaCycloneritidaCyclophoridae

Maassen, 2006

“Alycaeuscarinata Brandt”: unpublished name [not Maassen (2006b: 137, 138, figs 10–13)].
Alycaeus rolfbrandti Maassen, 2006b: 136, 137, figs 6–9. Type locality: limestone hills 20 km E. of Takek, Laos [Thakhek District, Khammouan Province, Laos]. Páll-Gergely et al. 2017b: 10, fig. 3b.

############# Material examined.

Holotype RMNH 104423 Brandt ex. Hemmen collection figured in Maassen (2006b: figs 6–9) and paratype RMNH 104425 (1 shell). Specimens from Tam Xieng Liab Cave, Thakhek District, Khammouan Province (Fig. 3F).

############# Distribution.

Known from the limestone karsts in Khammouan and Bolikhamxay Provinces, Laos (Maassen 2006b).

############# Remarks.

Brandt collects these shells in November 1963 from Laos and recognised this as a new species, but the finding was not published. Later, Maassen (2006b) described this species from Brandt’s specimens in the Hemmen collection.

############ Alycaeus vanbuensis

Taxon classificationAnimaliaCycloneritidaCyclophoridae

Bavay & Dautzenberg, 1900

Alycaeus (Dyorix [sic]) vanbuensis Bavay & Dautzenberg, 1900b: 120. Type locality: Van-Bu, Tonkin [Van Ban District, Lao Cai Province, Vietnam].
Alycaeus (Dioryx) vanbuensis : Bavay and Dautzenberg 1900a: 455, 456, pl. 11, figs 19–21.
Dioryx vanbuensis : Kobelt 1902a: 340. Do et al. 2015: 120, fig. 2c.
Alycaeus vanbuensis : Páll-Gergely et al. 2017b: 10, fig. 3c.

############# Material examined.

Syntype MNHN-IM-2000-31798 from “Van Bu” (1 shell; Fig. 4C). Specimens from Tam Ka Rao Cave, Ban Nam Air village, Vieng Phouka District, Luang Namtha Province (Fig. 4D). Specimens from Tam Phatok Cave, Ngoy District, Luang Phrabang Province (Fig. 4E).

############# Distribution.

Vietnam (Kobelt 1902a, Do et al. 2015).

########### *Dicharax* Kobelt & Möllendorff, 1900

############ Dicharax abdoui

Taxon classificationAnimaliaCycloneritidaCyclophoridae

Páll-Gergely, 2017

Dicharax abdoui Páll-Gergely in Páll-Gergely et al. 2017b: 14, fig. 6. Type locality: approx. 9 km northeast of Thakhek, Khammouane Province, Laos.

############# Material examined.

Holotype MNHN IM-2012-27329 (Fig. 4F).

############# Distribution.

Known only from the type locality in Laos (Páll-Gergely et al. 2017b).

############# Remarks.

No material of this species was found, and only the type specimens were examined.

############ Dicharax depressus

Taxon classificationAnimaliaCycloneritidaCyclophoridae

(Bavay & Dautzenberg, 1912)

Alycaeus depressus Bavay & Dautzenberg, 1912: 51–52, pl. 6, figs 10–13. Type locality: Pac-Kha, Tonkin [Pa Kha in Long Luong Commune, Van Ho District, Son La Province, Vietnam]. Saurin 1953: 113.
Dicharax depressus : Páll-Gergely et al. 2017b: 43–45, figs 12e, f, 13d, 28e–h, 29e, f, 31a–c.

############# Material examined.

**Figure 5.**
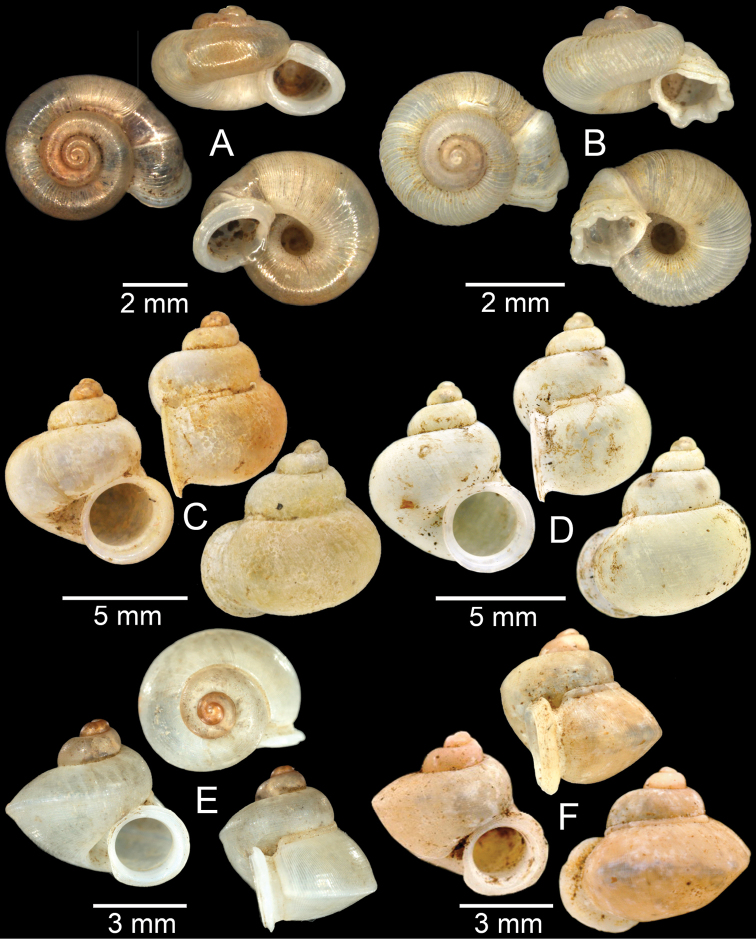
**A***Dicharaxdepressus*, syntype MNHN-IM-2012-27165 **B***Dicharaxfimbriatus*, syntype MNHN-IM-2012-27166 **C, D***Dioryxbacca***C** NHMUK 1903.7.1.2714 and **D** CUMZ collection **E, F***Dioryxcariniger***E** lectotype SMF 171804 and **F** CUMZ collection. Photos: B. Páll-Gergely (**A, B, E**).

Syntype MNHN IM-2012-27165 from “Pac-Kha, Tokin” (1 shell; Fig. 5A).

############# Distribution.

Laos and Vietnam (Saurin 1953, Páll-Gergely et al. 2017b).

############# Remarks.

No material of this species was found, and only the type specimen was examined.

############ Dicharax fimbriatus

Taxon classificationAnimaliaCycloneritidaCyclophoridae

(Bavay & Dautzenberg, 1912)

Alycaens [sic] (Charax) fimbriatus Bavay & Dautzenberg, 1912: 52–53, pl. 6, figs 14–17. Type locality: Pac-Kha [Pa Kha in Long Luong Commune, Van Ho District, Son La Province, Vietnam].
Charax fimbriatus : Saurin 1953: 113.
Chamalycaeus aff. fimbriatus : Solem 1966: 12.
Dicharax fimbriatus : Páll-Gergely et al. 2017b: 54–61, figs 13e, 35–37, 38a–d, 39.

############# Material examined.

Syntype MNHN IM-2012-27166 from “Pac-Kha” (1 shell; Fig. 5B).

############# Distribution.

China, Laos, Thailand and Vietnam (Saurin 1953, Solem 1966, Páll-Gergely et al. 2017b).

############# Remarks.

No material of this species was found, and only the type specimen was examined.

########### *Dioryx* Benson, 1859

############ Dioryx bacca

Taxon classificationAnimaliaCycloneritidaCyclophoridae

(Pfeiffer, 1863)

Alycaeus (Dioryx) bacca Pfeiffer, 1863a[1862]: 275, 276. Type locality: Lao Mountains, Camboja [Cambodia or Laos]. Pfeiffer 1863b: 229, pl. 59, figs 12–14.
Dioryx bacca : Kobelt 1902a: 337. Solem 1966: 12. Páll-Gergely et al. 2017b: 10.

############# Material examined.

Specimen NHMUK 1903.7.1.2714 from “Lao Mts.” (1 shell; Fig. 5C) Specimens from Ban Homexay village, road to Laos-Thailand border, Ngeun District, Xayaboury Province (Fig. 5D).

############# Distribution.

Laos and Thailand (Kobelt 1902a, Solem 1966).

############ Dioryx cariniger

Taxon classificationAnimaliaCycloneritidaCyclophoridae

Möllendorff, 1897

Dioryx cariniger Möllendorff, 1897: 41. Type locality: Prope Oppidum Luang-Prabang in regione Laos dicta [near Luang Phrabang District, Luang Phrabang Province, Laos]. Kobelt 1902a: 337. Zilch 1957: 141, pl. 5, fig. 1. Páll-Gergely et al. 2017b: 10.

############# Material examined.

Lectotype SMF 171804 (Fig. 5E) and paralectotype 109264 (1 shell). Specimens from Tam Xieng Liab Cave, Thakhek District, Khammouan Province (Fig. 5F).

############# Distribution.

Laos (Kobelt 1902a).

############ Dioryx messageri

Taxon classificationAnimaliaCycloneritidaCyclophoridae

(Bavay & Dautzenberg, 1900)

Alycaeus (Dioryx) messageri Bavay & Dautzenberg, 1900b: 119. Type locality: That-Khe [That Khe Town, Trang Dinh District, Lang Son Province, Vietnam]. Bavay and Dautzenberg 1900a: 453, pl. 11, figs 7, 8.
Dioryx messageri : Kobelt 1902a: 339. Do et al. 2015: 118, 120, fig. 2b. Páll-Gergely et al. 2017b: 10, fig. 4b.

############# Material examined.

**Figure 6.**
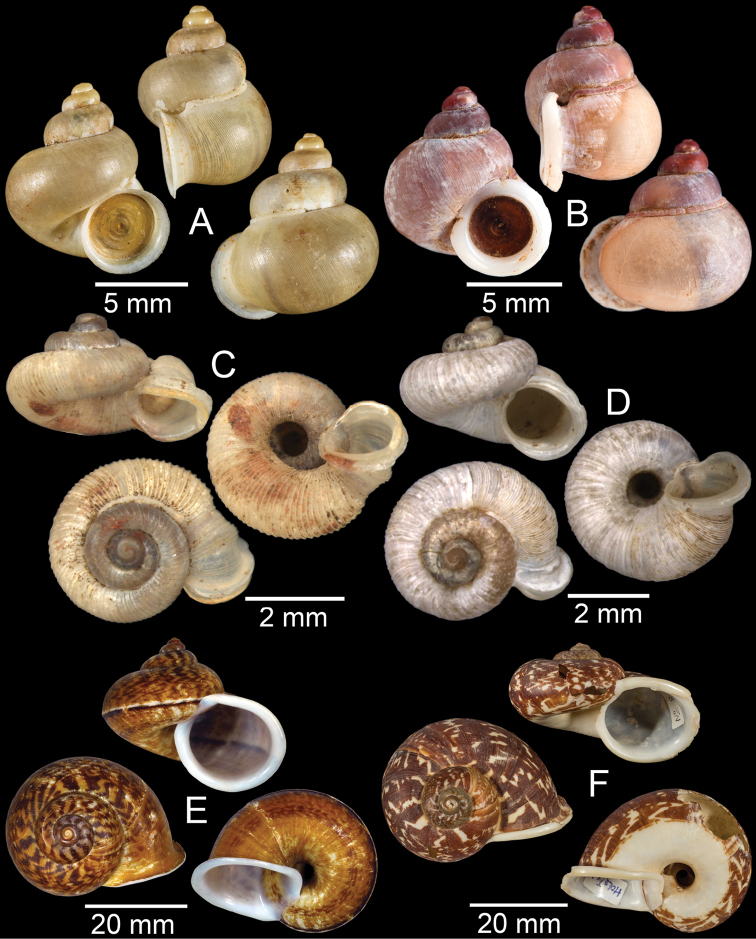
**A, B***Dioryxmessageri***A** syntype MNHN-IM-2000-31785 and **B** CUMZ collection **C***Metalycaeusheudei*, syntype MNHN-IM-2012-27169 **D***Metalycaeuslaosensis*, holotype MNHN-IM-2012-27172 **E***Cyclophorusfloridus*, CUMZ collection **F***Cyclophorusfranzhuberi*, holotype MNHN-IM-2000-33194. Photos: B. Páll-Gergely (**C, D**).

Syntype MNHN-IM-2000-31785 from “That-Khe” (1 shell; Fig. 6A). Specimen CUMZ from Tam Pou Kham Cave, Vangvieng District, Vientiane Province (Fig. 6B).

############# Distribution.

Vietnam (Kobelt 1902a, Do et al. 2015).

############# Remarks.

This species has a shell morphology very similar to *Dioryxbacca*, except it has a relatively larger shell and a shorter sutural tube.

########### *Metalycaeus* Pilsbry, 1900

############ Metalycaeus heudei

Taxon classificationAnimaliaCycloneritidaCyclophoridae

(Bavay & Dautzenberg, 1900)

Alycaeus (Charax) heudei Bavay & Dautzenberg, 1900b: 121, 122. Type locality: Haut-Tonkin [North Vietnam]. Bavay and Dautzenberg 1900a: 458–460, pl. 11, figs 15–18.
Alycaeus (Dicharax) heudei : Kobelt 1902a: 372.
Metalycaeus heudei : Páll-Gergely et al. 2017b: 74–84, figs 49c, d, 50–52, 53c, d.

############# Material examined.

Syntype MNHN IM-2012-27169 from “Haut-Tonkin” (1 shell; Fig. 6C).

############# Distribution.

China, Laos and Vietnam (Páll-Gergely et al. 2017b).

############# Remarks.

No material of this species was found, and only the type specimen was examined.

############ Metalycaeus laosensis

Taxon classificationAnimaliaCycloneritidaCyclophoridae

Páll-Gergely, 2017

Metalycaeus laosensis Páll-Gergely in Páll-Gergely et al. 2017b: 86, 87, fig. 47c. Type locality: old forest near stream approx. 1 km southwest of a stream and Nam Ou (river) confluence, Phongsaly Province, Laos.

############# Material examined.

Holotype MNHN IM-2012-27172 (Fig. 6D).

############# Distribution.

Known from several localities in Phongsaly Province, Laos (Páll-Gergely et al. 2017b).

############# Remarks.

No material of this species was found, and only the type specimens were examined.

########## Subfamily Cyclophorinae Gray, 1847

########### *Cyclophorus* Montfort, 1810

############ Cyclophorus floridus

Taxon classificationAnimaliaCycloneritidaCyclophoridae

(Pfeiffer, 1855)

Cyclostoma (Cyclophorus) floridum Pfeiffer, 1855[1854]: 300. Type locality: unknown.
Cyclophorus (Cyclophorus) floridus : Kobelt 1902a: 138.

############# Material examined.

Specimens from Ban Na Phong village, Pakkading District, Bolikhamxay Province (Fig. 6E).

############# Distribution.

Thailand (Kobelt 1902a).

############ Cyclophorus franzhuberi

Taxon classificationAnimaliaCycloneritidaCyclophoridae

Thach, 2017

Cyclophorus franzhuberi Thach, 2017: 14, figs 54–56. Type locality: Thakhek, Khammouane Province, Central Laos.

############# Material examined.

Holotype MNHN-IM-2000-33194 (Fig. 6F).

############# Distribution.

Known only from the type locality in Laos (Thach 2017).

############# Remarks.

No material of this species was found, and only the type specimens were examined.

############ Cyclophorus fulguratus

Taxon classificationAnimaliaCycloneritidaCyclophoridae

(Pfeiffer, 1854)

Cyclostoma (Cyclophorus) fulguratum Pfeiffer, 1854[1852]: 63. Type locality: unknown.
Cyclophorus fulguratus : Pfeiffer 1869: 440, 441, pl. 98, figs 1, 2. Nantarat et al. 2014: 11, fig. 8a, b.
Cyclophorus (Glossostylus) fulguratus : Kobelt 1902a: 112. Solem 1966: 10.

############# Material examined.

**Figure 7.**
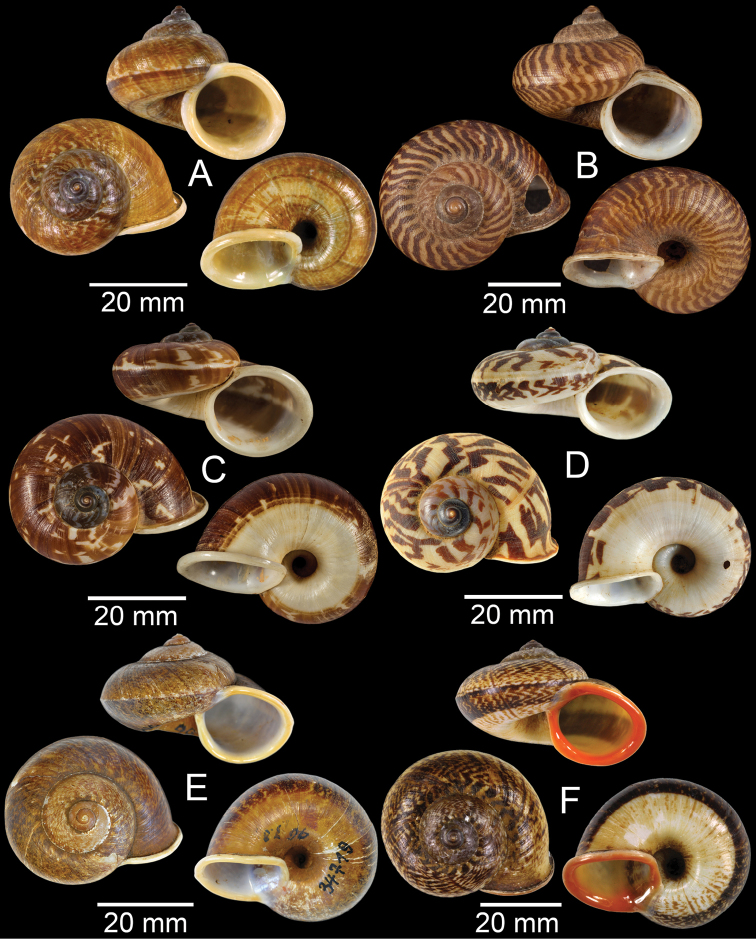
**A***Cyclophorusfulguratus*, CUMZ collection **B***Cyclophoruskhongensis*, holotype MNHN-IM-2000-33202 **C, D***Cyclophorusmansuyi***C** syntype MNHN-IM-2000-33835 and **D** CUMZ collection **E, F***Cyclophorusorthostylus***E** lectotype SMF 34719 and **F** CUMZ collection.

Lectotype NHMUK 20130117/1 and paralectotypes NHMUK 20130117/2-3 (2 shells) figured in Nantarat et al. (2014: fig. 8a, b). Specimens from Hot Spring, Ban Napair, Lak 20, Khamkeut District, Bolikhamxay Province (Fig. 7A).

############# Distribution.

Myanmar, Thailand and Vietnam (Kobelt 1902a, Solem 1966).

############ Cyclophorus khongensis

Taxon classificationAnimaliaCycloneritidaCyclophoridae

Thach & Huber, 2017

Cyclophorus khongensis Thach & Huber in Thach, 2017: 14, 15, figs 59–61. Type locality: Khong Island on Mekong River, Champasak Province, South Laos [Khong District, Champasak Province, Laos].

############# Material examined.

Holotype MNHN-IM-2000-33202 (Fig. 7B).

############# Distribution.

Known only from the type locality in Laos (Thach 2017).

############# Remarks.

No material of this species was found, and only the type specimens were examined.

############ Cyclophorus mansuyi

Taxon classificationAnimaliaCycloneritidaCyclophoridae

Dautzenberg & Fischer, 1908

Cyclophorus mansuyi Dautzenberg & Fischer, 1908: 204, 205, pl. 8, figs 1–4. Type locality: Quang-Huyen [Quang Uyen District, Cao Bang Province, Vietnam].

############# Material examined.

Syntype MNHN-IM-2000-33835 from “Quang-Huyen” (1 shell; Fig. 7C). Specimens from Tam Mungkorn Cave, Khamkeut District, Bolikhamxay Province (Fig. 7D).

############# Distribution.

Known only from the type locality in Vietnam (Dautzenberg and Fischer 1908).

############ Cyclophorus orthostylus

Taxon classificationAnimaliaCycloneritidaCyclophoridae

Möllendorff, 1898

Cyclophorus (Litostylus) orthostylus Möllendorff, 1898: 80, 81. Type locality: Boloven [Boloven Plateau, Paksong District, Champasak Province, Laos]. Kobelt 1902a: 101, 102. Zilch 1956a: 34.

############# Material examined.

**Figure 8.**
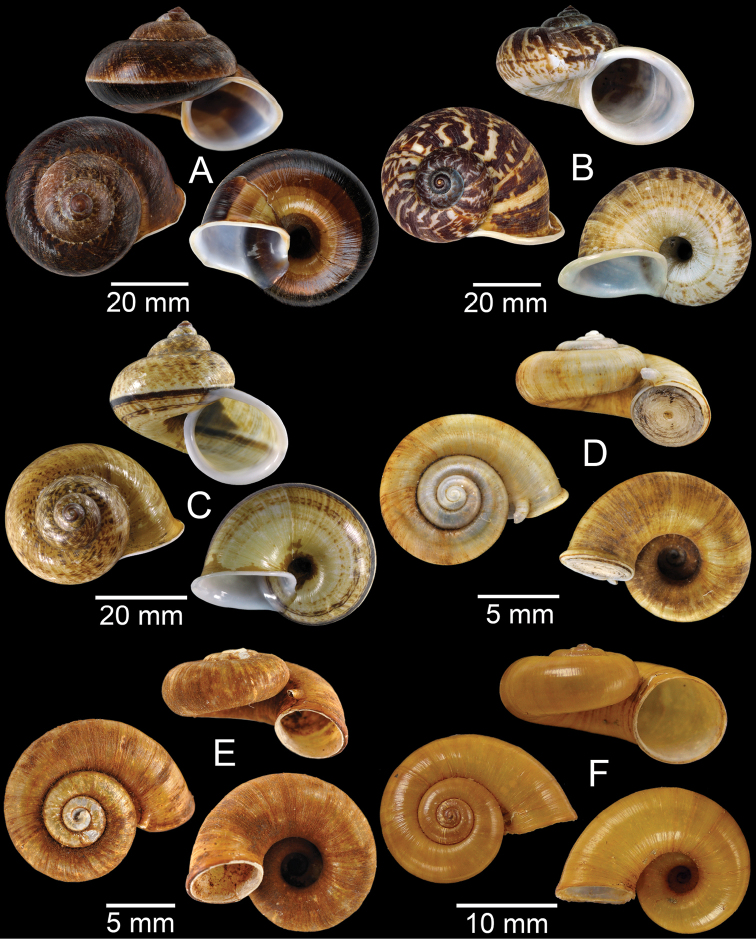
**A***Cyclophorusorthostylus*, CUMZ collection **B***Cyclophorussiamensis*, CUMZ collection **C***Cyclophorusvolvulus*, CUMZ collection **D, E***Cyclotusbernardii***D** NHMUK ex Cuming collection and **E** CUMZ collection **F***Cyclotusporrectus*, Holotype SMF 132373.

Lectotype SMF 34719 (Fig. 7E). Specimen from Ban Phone village, Lamam District, Sekong Province (Figs 7F, 8A).

############# Distribution.

Laos and Vietnam (Kobelt 1902a, Zilch 1956a)

############ Cyclophorus siamensis

Taxon classificationAnimaliaCycloneritidaCyclophoridae

(Sowerby I, 1850)

Cyclostoma siamense Sowerby I, 1850: 158, pl. 31a, figs 292, 293. Type locality: Siam [Thailand].
Cyclophorus (Salpingophorus) siamensis : Kobelt 1902a: 132, 133.
Cyclophorus siamensis : Nantarat et al. 2014: 23, fig. 20a, b.

############# Material examined.

Lectotype NHMUK 20130088/1 and paralectotype NHMUK 20130088/2 (1 shell) figured in Nantarat et al. (2014: fig. 20a, b). Specimens from Ban Na Phong village, Pakkading District, Bolikhamxay Province (Figs 8B, 18A).

############# Distribution.

India and Thailand (Kobelt 1902a, Nantarat et al. 2014).

############ Cyclophorus volvulus

Taxon classificationAnimaliaCycloneritidaCyclophoridae

(Müller, 1774)

Helix volvulus Müller, 1774: 82. Type locality: unknown.
Cyclophorus (Cyclophorus) volvulus : Kobelt 1902a: 143, 144.
Cyclophorus volvulus : Do et al. 2015: 120, 122, fig. 3b.

############# Material examined.

Specimens from Pathoumphone District, Champasak Province (Figs 8C, 18B).

############# Distribution.

Malaysia, Thailand and Vietnam (Kobelt 1902a, Do et al. 2015).

########### *Cyclotus* Swainson, 1840

############ Cyclotus bernardii

Taxon classificationAnimaliaCycloneritidaCyclophoridae

(Pfeiffer, 1862)

Rhiostoma bernardii Pfeiffer, 1862: 45, 46, pl. 6, fig. 5. Type locality: Siam [Thailand]. Kobelt 1902a: 177.

############# Material examined.

Specimens from unknown locality NHMUX ex. Cuming collection (1 shell; Fig. 8D). Specimens from Ban Phone Pai village, Bachiang District, Champasak Province (Fig. 8E).

############# Distribution.

Thailand (Kobelt 1902a)

############ Cyclotus porrectus

Taxon classificationAnimaliaCycloneritidaCyclophoridae

Möllendorff, 1898

Cyclotus (Procyclotus) porrectus Möllendorff, 1898: 84. Type locality: Boloven [Boloven Plateau, Paksong District, Champasak Province, Laos]. Kobelt 1902a: 207. Kobelt 1912: 852, 853, pl. 128, figs 21–23. Zilch 1956b: 188.

############# Material examined.

Holotype SMF 132373 (Fig. 8F).

############# Distribution.

Laos (Kobelt 1902a).

############# Remarks.

No material of this species was found, and only the type specimen was examined.

########### *Lagocheilus* Blanford, 1864

############ Lagocheilus conicus

Taxon classificationAnimaliaCycloneritidaCyclophoridae

(Martens, 1860)

Cyclotus conicus Martens, 1860: 10. Type locality: Siam [Thailand].
Japonia (Lagochilus [sic]) *conica*: Kobelt 1902a: 40.

############# Material examined.

**Figure 9.**
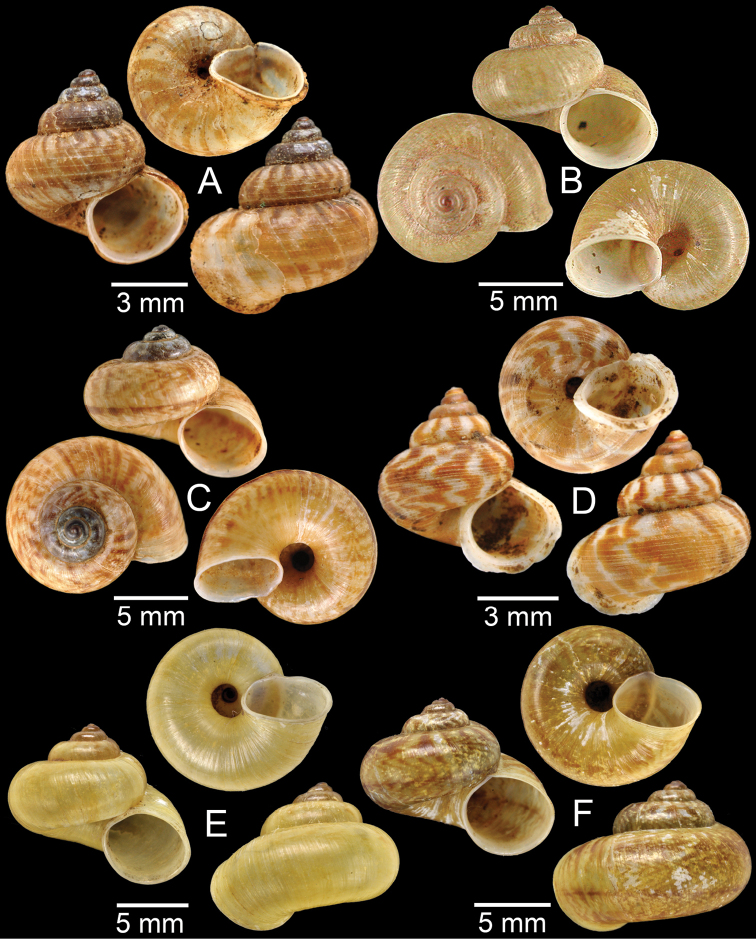
**A***Lagocheilusconicus*, CUMZ collection **B, C***Lagocheilusklobukowskii***B** syntype MNHN-IM-2000-26699 and **C** CUMZ collection **D***Lagocheiluslandesi*, CUMZ collection **E, F***Lagocheiluslaomontanus*, possible syntypes NHMUK ex. Cuming collection.

Specimens from Tam Xieng Liab Cave, Thakhek District, Khammouan Province (Fig. 9A).

############# Distribution.

Thailand (Kobelt 1902a).

############ Lagocheilus klobukowskii

Taxon classificationAnimaliaCycloneritidaCyclophoridae

(Morlet, 1885)

Cyclophorus klobukowskii Morlet, 1885[1884]: 391, 392, pl. 12, fig. 1. Type locality: Près des rapides de Kamchay, aux environs de la grotte de Kébal-Réméas (route de Kampot à Hatien); trouvé communément sur les montagnes, dans les forêts, jusqu’à Compong-Som, et sur les rives de Tap-Chéang [In the area of Preah Sihanouk and Kampot Provinces, Cambodia].
Japonia (Lagochilus [sic]) klobukowskii: Kobelt 1902a: 46, 47.

############# Material examined.

Syntype MNHN-IM-2000-26699 (1 shell; Fig. 9B). Specimens from Tam Pew Cave, Kham District, Xieng Khaung Province (Figs 9C, 18C).

############# Distribution.

Cambodia (Kobelt 1902a).

############ Lagocheilus landesi

Taxon classificationAnimaliaCycloneritidaCyclophoridae

(Morlet, 1885)

Cyclophorus landesi Morlet, 1885[1884]: 392, 393, pl. 11, figs 5, 5a. Type locality: extrémité de la chaîne de ľÉléphant, non loin de la mer [probably refers to the Damrei Mountains, south of Cardamom Ranges, Cambodia].
Japonia (Lagochilus [sic]) landesi: Kobelt 1902a: 47.

############# Material examined.

Specimens from km 30, Laos-Vietnam border road, Yommalath District, Khammouan Province (Fig. 9D).

############# Distribution.

Cambodia (Kobelt 1902a).

############ Lagocheilus laomontanus

Taxon classificationAnimaliaCycloneritidaCyclophoridae

(Pfeiffer, 1863)

Cyclophorus laomontanus Pfeiffer, 1863a[1862]: 276. Type locality: Lao Mountains, Camboja [Cambodia or Laos].
Japonia (Lagochilus [sic]) laomontana: Kobelt 1902a: 47.

############# Material examined.

Possible syntypes NHMUK ex. Cuming collection from “Lao Mountains, Camboja” (2 shells; Fig. 9E, F).

############# Distribution.

Laos (Kobelt 1902a).

############# Remarks.

No material of this species was found, and only the possible type specimens were examined. This species has a shell morphology very similar to *Lagocheilusklobukowskii*. Further taxonomic revision is needed to clarify their taxonomic statuses.

############ Lagocheilus michaui

Taxon classificationAnimaliaCycloneritidaCyclophoridae

(Crosse & Fischer, 1863)

Leptopoma michaui Crosse & Fischer, 1863b: 367–369, pl. 14, fig. 7. Type locality: insula Poulo-Condor dicta, Cochinchine [Con Dao Islands, Ba Ria–Vung Tau Province, Vietnam].
Japonia (Japonia) michaui : Kobelt 1902a: 62.

############# Material examined.

**Figure 10.**
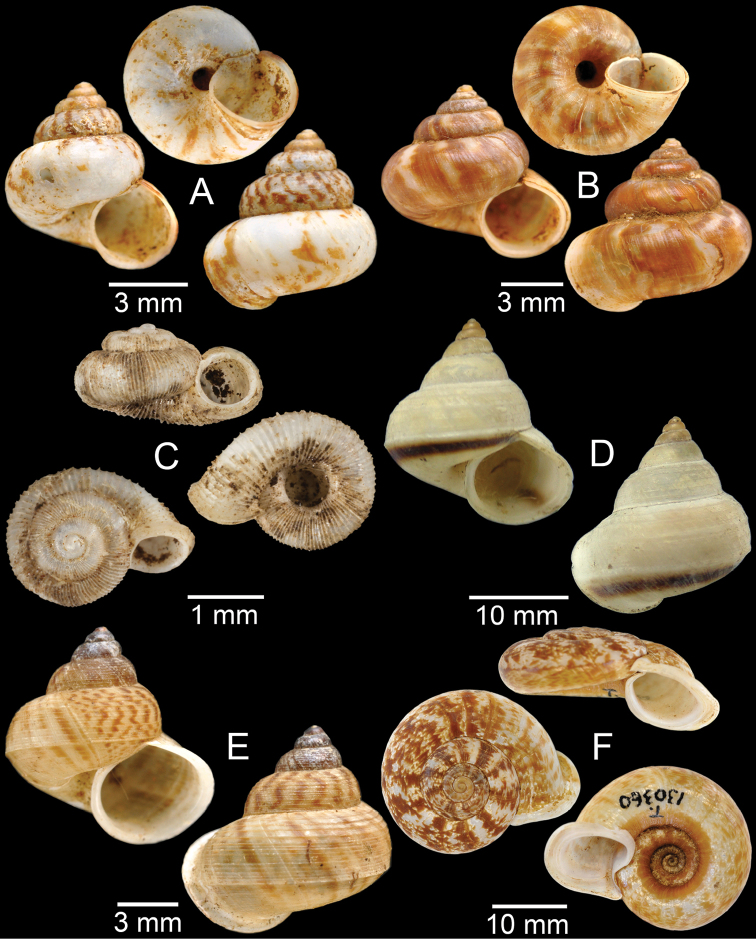
**A***Lagocheilusmichaui*, CUMZ collection **B***Lagocheilusscissimargo*, CUMZ collection **C***Laotiapahiensis*, syntype MNHN-IM-2000-28217 **D, E***Leptopomaannamiticum***D** lectotype SMF 126975 and **E** CUMZ collection **F***Ptychopomabathyschisma*, lectotype SMF 130360.

Specimens from Nam Ork Roo, Ban Nathong village, Namo District, Oudomxay Province (Fig. 10A).

############# Distribution.

Vietnam (Kobelt 1902a).

############ Lagocheilus scissimargo

Taxon classificationAnimaliaCycloneritidaCyclophoridae

(Benson, 1856)

Cyclophorus (?) scissimargo Benson, 1856a: 228. Type locality: Phie Than vallis Tenasserim [Payathonzu or Phaya Thone Zu Town, Kyain Seikgyi Township, Kawkareik District, Kayin State, Myanmar].
Cyclophorus scissimargo : Pfeiffer 1860b: 144, pl. 37, figs 19–21.
Japonia (Lagochilus [sic]) scissimargo: Kobelt 1902a: 53, 54.
Lagochilus [sic] scissimargo: Saurin 1953: 113.
Japonia scissimargo : Do et al. 2015: 122.

############# Material examined.

Specimens from Tam Xang Cave, Thakhek District, Khammouan Province (Fig. 10B).

############# Distribution.

Cambodia, Laos, Myanmar and Vietnam (Kobelt 1902a, Saurin 1953, Do et al. 2015).

############# Remarks.

This species is the type species of *Lagocheilus* Blanford, 1864.

########### *Laotia* Saurin, 1953

############ Laotia pahiensis

Taxon classificationAnimaliaCycloneritidaCyclophoridae

Saurin, 1953

Laotia pahiensis Saurin, 1953: 113, 114, pl. 4, figs 1a–c, 2a–c. Type locality: environs du village méo de Pah Hia, à 100 kilomètres au Sud de Xieng-Khouang, chef-lieu de la province du Tran Ninh, Laos [probably refers to Ban Namthong, Longchaeng District, Xaisomboun Province, Laos]. Páll-Gergely 2014: 290, figs 1, 2.

############# Material examined.

Syntypes MNHN-IM-2000-28217 from “Pah Hia” (2 shells; Fig. 10C).

############# Distribution.

Known only from the type locality in Laos (Saurin 1953).

############# Remarks.

No material of this species was found, and only the type specimens were examined. For the current interpretation of Pa Hia, see Páll-Gergely et al. (2016: 13).

########### *Leptopoma* Pfeiffer, 1847

############ Leptopoma annamiticum

Taxon classificationAnimaliaCycloneritidaCyclophoridae

Möllendorff, 1900

Leptopoma (Trocholeptopoma) annamiticum Möllendorff, 1900: 134. Type locality: Insel Bay-Min [Bay Min Island, Ha Long Provincial, Quang Ninh Province, Vietnam]. Kobelt 1902a: 18. Zilch 1954b: 147, pl. 16, fig. 48. Solem 1966: 9.

############# Material examined.

Lectotype SMF 126975 (Fig. 10D) and paralectotypes SMF 126976 (1 shell), SMF 126977 (3 shells). Specimens from km 30, Laos-Vietnam border road, Yommalath District, Khammouan Province (Fig. 10E).

############# Distribution.

Thailand and Vietnam (Kobelt 1902a, Solem 1966).

########### *Ptychopoma* Möllendorff, 1885

############ Ptychopoma bathyschisma

Taxon classificationAnimaliaCycloneritidaCyclophoridae

(Möllendorff, 1898)

Pterocyclus [sic] bathyschisma Möllendorff, 1898: 82, 83. Type locality: Boloven [Boloven Plateau, Paksong District, Champasak Province, Laos]. Kobelt 1902a: 162. Kobelt 1911: 743, pl. 108, figs 4–6.
Pterocyclos bathyschisma : Zilch 1956b: 171, 172, pl. 12, fig. 1.
Ptychopoma bathyschisma : Foon 2015: 7–10, fig. 2.

############# Material examined.

Lectotype SMF 130360 (Fig. 10F) and paralectotypes SMF 130361 (2 shells).

############# Distribution.

Laos (Foon 2015).

############# Remarks.

No material of this species was found, and only the type specimens were examined.

########### *Rhiostoma* Benson, 1860

############ Rhiostoma marioni

Taxon classificationAnimaliaCycloneritidaCyclophoridae

(Ancey, 1898)

Pterocyclos marioni Ancey, 1898: 137, pl. 9, fig. f. Type locality: Luang-prabang, Laos and Mont Hou, Tonkin [Luang Phrabang Province, Laos and Muang Khua District, Phongsaly Province, Laos]. Wood and Gallichan 2008: 64, pl. 25, figs 3, iv (label).
Pterocyclus [sic] marioni: Kobelt 1911: 757.

############# Material examined.

**Figure 11.**
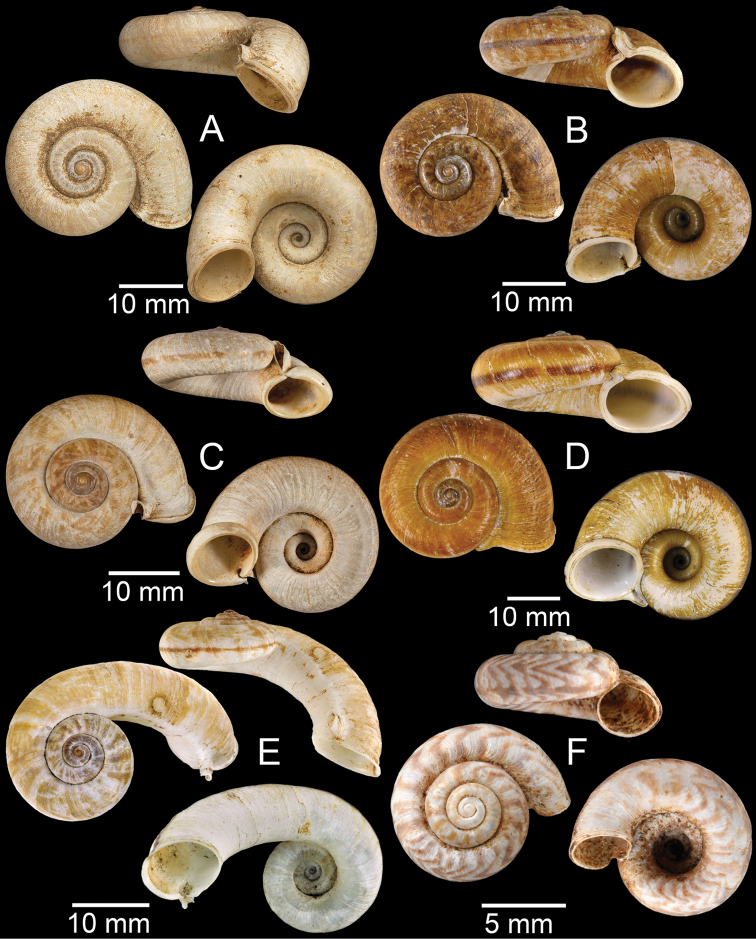
**A, B***Rhiostomamarioni***A** syntype NMW 1955.158.24090 and **B** CUMZ collection **C, D***Rhiostomamorleti***C** syntype MNHN-IM-2000-20961 and **D** CUMZ collection **E***Rhiostoma* sp., CUMZ collection **F***Scabrinalaotica*, CUMZ collection.

Syntype NMW 1955.158.24090 from “Luang-prabang, Laos” (1 shell; Fig. 11A). Specimens from Ban Homexay village road to Laos-Thailand border, Ngeun District, Xayaboury Province (Figs 11B, 18E).

############# Distribution.

Laos (Kobelt 1911).

############ Rhiostoma morleti

Taxon classificationAnimaliaCycloneritidaCyclophoridae

Dautzenberg & Fischer, 1906

Rhiostoma morleti Dautzenberg & Fischer, 1906[1905]: 429–431, pl. 10, figs 1–4. Type locality: Luang-Prabang, Laos; Ha Giang, Tonkin [Ha Giang Province, Vietnam]. Kobelt 1911: 755, 756, pl. 110, figs 1–4.

############# Material examined.

Syntype MNHN-IM-2000-20961 from “Laos” (1 shell; Fig. 11C). Specimens from Wat Pathammawath Sen Oudom, Lak 20 village, Khamkeut District, Bolikhamxay Province (Fig. 11D).

############# Distribution.

Laos and Vietnam (Kobelt 1911).

############ Rhiostoma

Taxon classificationAnimaliaCycloneritidaCyclophoridae

sp.

############# Material examined.

Specimens from limestone outcrops in Ngoy Town, Ngoy District, Luang Phrabang Province (Figs 11E, 18F).

############# Remarks.

These specimens differ from *Rhiostomamorleti*, *R.marioni*, *R.christae* Thach, 2016 and *R.herosae* Thach & Huber in Thach, 2017 from Laos and Vietnam in having a long, descending and curved detached-whorl (proboscis-like detached-whorl), an aperture opened subventrally, and with a short and complete tubular accessory respiratory structure close to the aperture. In contrast, these four nominal species have a short to absent detached-whorl, a complete tubular or canal-like accessory respiratory structure, and an aperture opened laterally.

########### *Scabrina* Blanford, 1863

############ Scabrina laotica

Taxon classificationAnimaliaCycloneritidaCyclophoridae

Möllendorff, 1897

Scabrina laotica Möllendorff, 1897: 35. Type locality: Prope Luang-Prabang regionis Lao [Luang Phrabang Province, Laos]. Kobelt 1902a: 89. Zilch 1955: 206, pl. 15, fig. 59.

############# Material examined.

Lectotype SMF 128489 figured in Zilch (1955: pl. 15, fig. 59) and paralectotype SMF 128490 (1 shell). Specimens from limestone hills at Ban Oudom village, Pakbeg Ditrict, Oudomxay Province (Fig. 11F).

############# Distribution.

Laos (Kobelt 1902a).

############ Scabrina patera

Taxon classificationAnimaliaCycloneritidaCyclophoridae

(Pfeiffer, 1854)

Cyclostoma (?) patera Pfeiffer, 1854[1852]: 61. Type locality: unknown.
Scabrina patera : Kobelt 1902a: 90.

############# Material examined.

**Figure 12.**
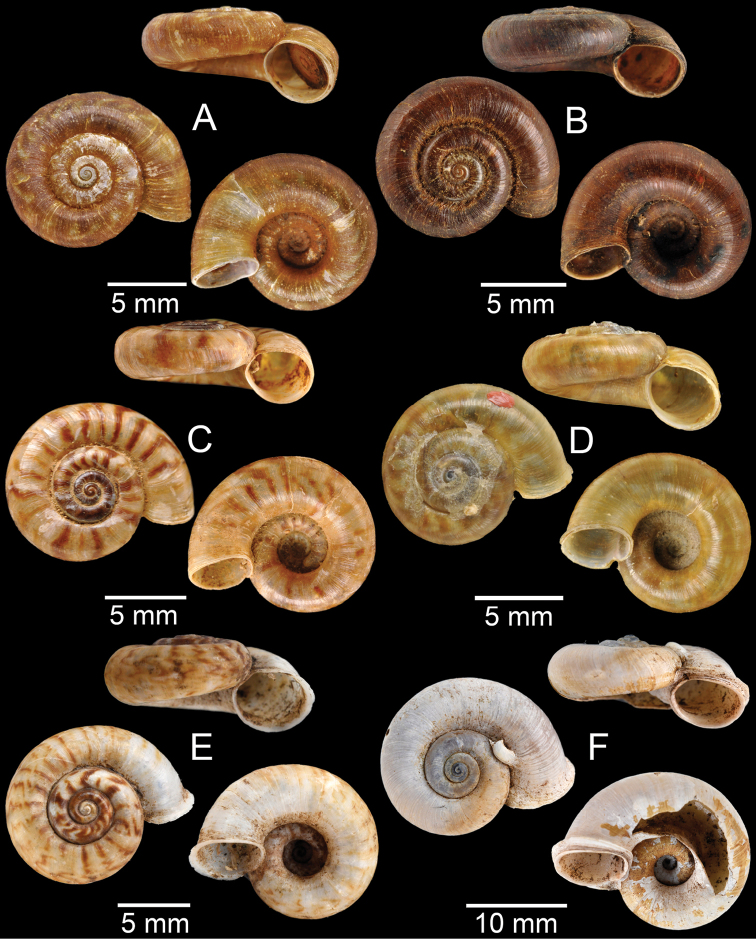
**A–C***Scabrinapatera***A** NHMUK collection and **B, C** CUMZ collection **D, E***Scabrinavanbuensis***D** syntype NHMUK 1896.1.25.7-8 and **E** CUMZ collection **F***Spiraculumvilvensi*, CUMZ collection.

Specimens NHMUK ex. Cuming collection from “Lao Mountain” (4 shells; Fig. 12A). Specimens from Ban Nong Tang village, Phookood District, Xieng Khaung Province (Fig. 12B, C).

############# Distribution.

Southern India, Cambodia, Laos and Myanmar (Kobelt 1902a).

############ Scabrina vanbuensis

Taxon classificationAnimaliaCycloneritidaCyclophoridae

(Smith, 1896)

Pterocyclus [sic] vanbuensis Smith, 1896: 130. Type locality: Vanbu, Tonkin [Van Ban District, Lao Cai Province, Vietnam].
Scabrina vanbuensis : Kobelt 1902a: 90, 91. Do et al. 2015: 124, fig. 4f.

############# Material examined.

Syntypes NHMUK 1896.1.25.7-8 from “Vanbu, Tonkin” (2 shells; Fig. 12D). Specimens from Ban Naweed village, Viengxay District, Houaphanh Province (Fig. 12E).

############# Distribution.

Vietnam (Kobelt 1902a, Do et al. 2015).

############ Spiraculum

Taxon classificationAnimaliaCycloneritidaCyclophoridae

Pearson, 1833

############# Remarks.

The name *Spiraculum* was nominated by Pearson (1833) with *Spiraculumhispidum* as a type species in order to replace the name *Pterocyclos* Benson, 1832. This was an invalid action and *Spiraculum* was treated as a junior subjective synonym or a subgenus to *Pterocyclos* by later authors (Benson 1836: 355–358, Pfeiffer 1852: 41, Adams and Adams 1855: 278). Blanford (1863) discovered and described the second species, *Spiraculumavanum* and thus regarded *Spiraculum* as a valid genus. Kobelt (1902a: 171) incorrectly proposed a new replacement name, *Pearsonia* to replace *Spiraculum* due to the synonym, as he stated in the footnote that “The previously used name *Spiraculum* was introduced by Pearson as a synonym of *Pterocyclos* Benson”. The name, *Pearsonia*, was subsequently adopted as a valid genus since then. However, this substitute name was invalid as the name *Spiraculum* was never preoccupied (ICZN 1999, Art. 60). Thus, *Spiraculum* is resurrected as a valid genus and *Pearsonia* is treated as a junior objective synonym to *Spiraculum*.

############ Spiraculum massiei

Taxon classificationAnimaliaCycloneritidaCyclophoridae

Morlet, 1892

Spiraculum massiei Morlet, 1892b: 85. Type locality: Mont Pou-Khiou, dans le Laos [Pou Khiou Mountain, Khamkeut District, Bolikhamxay Province, Laos]. Morlet 1893[1892]: 323, 324, pl. 8, figs 4, 4a–c.
Pearsonia (Pearsonia) massiei : Kobelt 1902a: 174.

############# Material examined.

**Figure 13.**
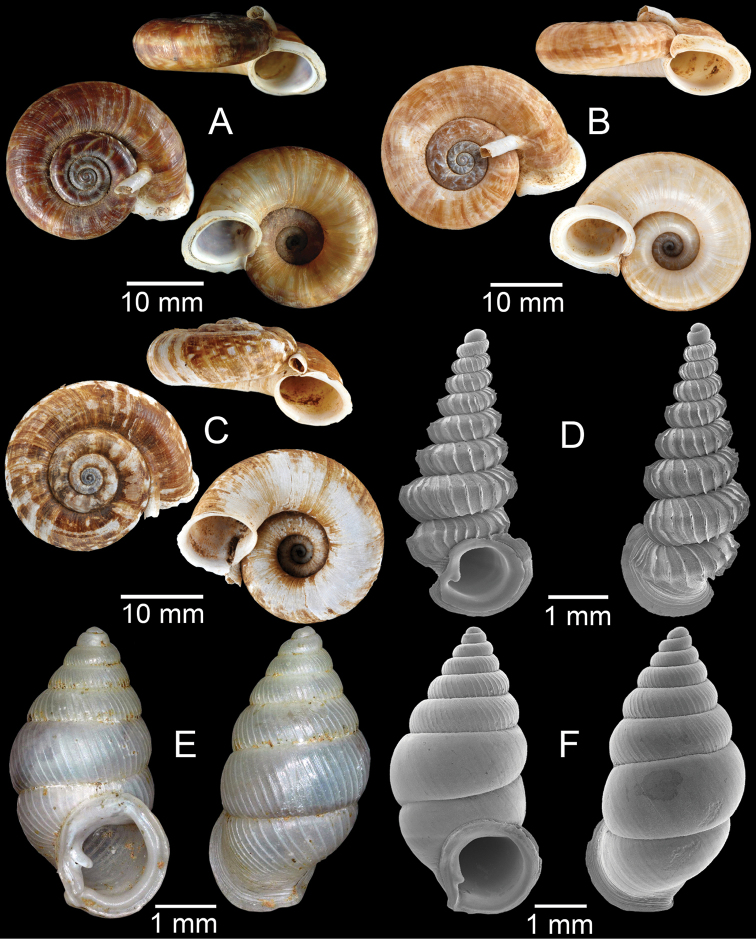
**A, B***Spiraculummassiei***A** syntype MNHN-IM-2000-20837 and **B** CUMZ collection **C***Spiraculum* sp., CUMZ collection **D***Diplommatinabelonis*, CUMZ collection **E, F***Diplommatinabifissurata***E** syntype MNHN-IM-2000-32429 and **F** CUMZ collection. Photo: B. Páll-Gergely (**E**).

Syntype MNHN-IM-2000-20837 from “Mont Pou-Khiou, dans le Laos” (1 shell; Fig. 13A). Specimens from Tam Mungkorn Cave, Khamkeut District, Bolikhamxay Province (Fig. 13B).

############# Distribution.

Laos (Kobelt 1902a).

############ Spiraculum vilvensi

Taxon classificationAnimaliaCycloneritidaCyclophoridae

(Thach & Huber, 2017)

Pearsonia vilvensi Thach & Huber in Thach, 2017: 16, figs 74, 75, 77. Type locality: suburb of Vang Vieng town, Ventiane Province, Central Laos [Vangvieng District, Vientiane Province, Laos].
Pearsonia “*viviensis*” Thach and Huber in Thach, 2017: 73 (figure captions).

############# Material examined.

Specimen from Tam Pou Kham Cave, Vangvieng District, Vientiane Province (Fig. 12F).

############# Distribution.

Known only from the type locality in Laos (Thach 2017).

############# Remarks.

There are two different original spellings “*vilvensi*” in the species description and “*viviensis*” in the figure caption. However, “*viviensis*” seemed to be an inadvertent error, since the author clearly proposed this species name in honor to “Claude Vilvens”.

############ Spiraculum

Taxon classificationAnimaliaCycloneritidaCyclophoridae

sp.

############# Material examined.

Specimens from Ban Phone Can village, Yommalath District, Khammouan Province (Figs 13C, 18D).

############# Remarks.

These specimens differ from *Spiraculummassiei* and *S.vilvensi* from Laos and *Pterocycloshuberi* Thach, 2015, *S.franzhuberi* (Thach, 2017) and *S.thachi* (Huber in Thach, 2017) from Vietnam in having a short complete tubular accessory respiratory structure located close to the aperture and projecting forward to the aperture. In comparison, *S.massiei* has a long complete tubular accessory respiratory structure located further away from the aperture and projecting up to the apex. *Spiraculumvilvensi* has a long complete tubular accessory respiratory structure laying in the suture and projecting backwards to the aperture, and an apertural lip expanded near the suture (see Thach (2017) for comparison). *Pterocycloshuberi* has an expanded lip forming a canal-like accessory respiratory structure and projecting forward to the aperture. *Spiraculumfranzhuberi* and *S.thachi* have both an expanded lip forming a canal-like accessory respiratory structure and a short tubular accessory respiratory structure located away from the aperture (see Thach (2017) for comparison).

######### Family Diplommatinidae Pfeiffer, 1857

########## *Diplommatina* Benson, 1849

########### Diplommatina belonis

Taxon classificationAnimaliaCycloneritidaDiplommatinidae

Möllendorff, 1900

Diplommatina belonis Möllendorff, 1900: 137. Type locality: Touranne [Danang Province, Vietnam]. Bavay and Dautzenberg 1904[1903]: 223, pl. 10, figs 9–12.
Diplommatina (Diplommatina) belonis : Kobelt 1902a: 426. Zilch 1953a: 19, pl. 8, fig. 124.

############ Material examined.

Lectotype SMF 105380 figured in Zilch (1953a: pl. 8, fig. 124) and paralectotype SMF 105381 (13 shells). Specimens from limestone near Tam Tarn Kaison Cave, Viengxay District, Houaphanh Province (Fig. 13D).

############ Distribution.

Vietnam (Kobelt 1902a).

########### Diplommatina bifissurata

Taxon classificationAnimaliaCycloneritidaDiplommatinidae

Bavay & Dautzenberg, 1912

Diplommatina bifissurata Bavay & Dautzenberg, 1912: 45, 46, pl. 5, figs 9, 10. Type locality: Nat-Son [Nat Son Commune, Kim Boi District, Hoa Binh Province, Vietnam], Cam-Duong [Cam Duong Commune, Lao Cai City, Lao Cai Province, Vietnam], Phong-Tho [Phong Tho District, Lai Chau Province, Vietnam].

############ Material examined.

Syntype MNHN-IM-2000-32429 from “Nat-Son” (1 shell; Fig. 13E). Specimens from Ban Naweed village, Viengxay District, Houaphanh Province (Fig. 13F).

############ Distribution.

Known from several localities in Vietnam (Bavay and Dautzenberg 1912).

########### Diplommatina clausilioides

Taxon classificationAnimaliaCycloneritidaDiplommatinidae

Bavay & Dautzenberg, 1912

Diplommatina clausilioides Bavay & Dautzenberg, 1912: 42, pl. 5, figs 3, 4. Type locality: Muong-Hum [Muong Hum Commune, Bat Xat District, Lao Cai Province, Vietnam]. Do et al. 2015: 124, fig. 5b.

############ Material examined.

**Figure 14.**
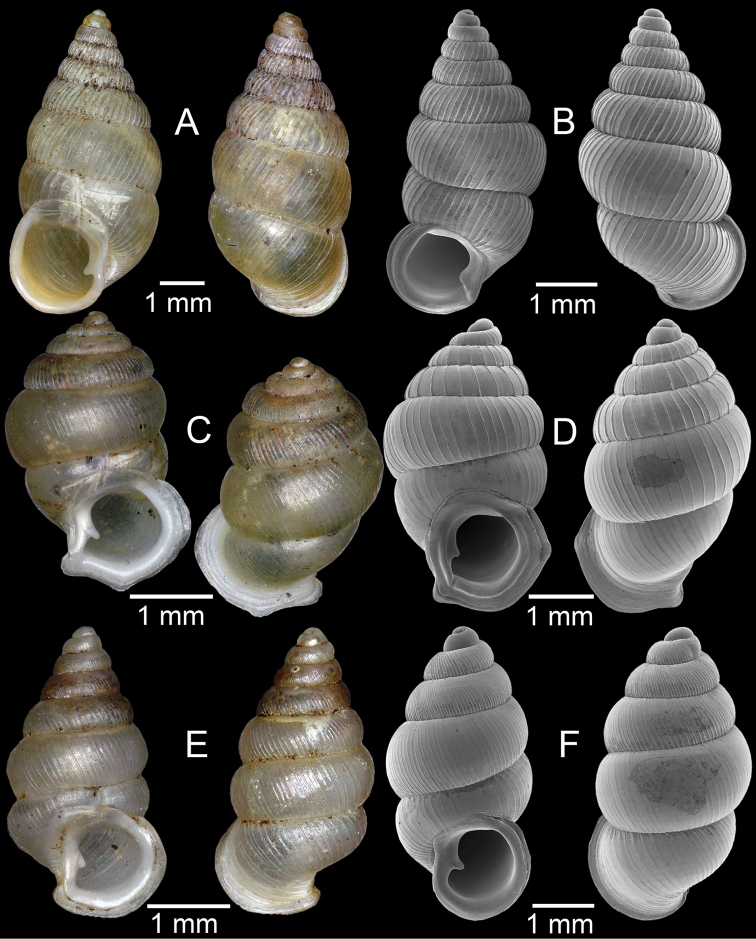
**A, B***Diplommatinaclausilioides***A** syntype MNHN-IM-2000-32430 and **B** CUMZ collection **C, D***Diplommatinamessageri***C** syntype MNHN-IM-2000-9668 and **D** CUMZ collection **E***Diplommatinalemyrei*, syntype MNHN-IM-2000-32416 **F***Diplommatina* sp., CUMZ collection. Photos: B. Páll-Gergely (**A, C, E**).

Syntype MNHN-IM-2000-32430 from “Muong-Hum” (1 shell; Fig. 14A). Specimens from Tam Phatok Cave, Ngoy District, Luang Phrabang Province (Fig. 14B).

############ Distribution.

Vietnam (Do et al. 2015).

########### Diplommatina lemyrei

Taxon classificationAnimaliaCycloneritidaDiplommatinidae

Bavay & Dautzenberg, 1904

Diplommatina lemyrei Bavay & Dautzenberg, 1904[1903]: 227, pl. 11, figs 5, 6. Type locality: That-Khé [That Khe Town, Trang Dinh District, Lang Son Province, Vietnam]. Saurin 1953: 113.

############ Material examined.

Syntype MNHN-IM-2000-32416 from “That-Khé” (1 shell; Fig. 14E).

############ Distribution.

Laos and Vietnam (Bavay and Dautzenberg 1904, Saurin 1953).

############ Remarks.

No material of this species was found, and only the type specimen was examined.

########### Diplommatina messageri

Taxon classificationAnimaliaCycloneritidaDiplommatinidae

Ancey, 1904

Diplommatina (Sinica) messageri Ancey in Bavay and Dutzenberg 1904[1903]: 224, 225, pl. 11, figs 1, 2. Type locality: Backan et That-Khé, Tonkin [Bac Kan Province and That Khe Town, Trang Dinh District, Lang Son Province, Vietnam]. Wood and Gallichan 2008: 66.
Diplommatina messageri : Saurin 1953: 113. Do et al. 2015: 126, fig. 5d.

############ Material examined.

Syntype MNHN-IM-2000-9668 from “Haut-Tonkin, Bac-Kan et That-Khé” (1 shell; Fig. 14C). Specimens from limestone cliff near Tam Tarn Kaison Cave, Viengxay District, Houaphanh Province (Fig. 14D).

############ Distribution.

Laos and several localities in Vietnam (Saurin 1953, Do et al. 2015).

############ Remarks.

For the correct authorship of the name, see Wood and Gallichan (2008: 66).

########### Diplommatina rotundata

Taxon classificationAnimaliaCycloneritidaDiplommatinidae

Saurin, 1953

Diplommatina rotundata Saurin, 1953: 114, 115, pl. 4, fig. 3a, b. Type locality: environs du village méo de Pah Hia, à 100 kilomètres au Sud de Xieng-Khouang, chef-lieu de la province du Tran Ninh, Laos [probably refers to Ban Namthong, Longchaeng District, Xaisomboun Province, Laos].

############ Distribution.

Known only from the type locality in Laos (Saurin 1953).

############ Remarks.

No material of this species was found, and the type specimen could not be traced. This species was figured in Saurin (1953: pl. 4, fig. 3a, see Fig. 17A). For the current interpretation of Pa Hia, see Páll-Gergely et al. (2016: 13).

########### Diplommatina

Taxon classificationAnimaliaCycloneritidaDiplommatinidae

sp.

############ Material examined.

Specimens from limestone cliff near Tam Tarn Kaison Cave, Viengxay District, Houaphanh Province (Fig. 14F).

############ Remarks.

These specimens differ from *Diplommatinabifissurata* in having less whorls (5 or 6), a circular aperture without anterior canal and protrusion, and with a strong columella tooth, while the latter species has 8 whorls, an anterior canal well developed resulting in a short protrusion on apertural lip, and with a small columellar tooth.

######### Family Pupinidae Pfeiffer, 1853

########## *Pollicaria* Gould, 1856

########### Pollicaria mouhoti

Taxon classificationAnimaliaCycloneritidaPupinidae

(Pfeiffer, 1863)

Hybocystis mouhoti Pfeiffer, 1863a[1862]: 276, pl. 36, fig. 13. Type locality: Lao Mountains, Camboja [Cambodia or Laos]. Pfeiffer 1863b: 227, 228, pl. 59, figs 5–8.
Pollicaria mouhoti : Kobelt 1902a: 290. Kongim et al. 2013: 31, 32, figs 2b, 3a–e, 4h, i, 6b.

############ Material examined.

**Figure 15.**
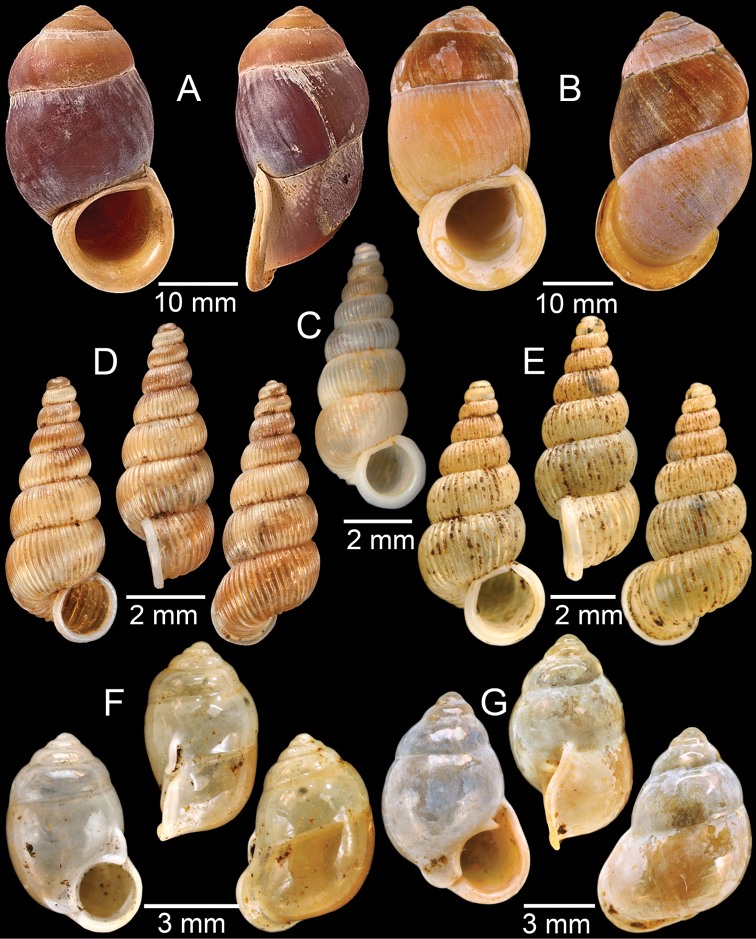
**A***Pollicariamouhoti*, lectotype NHMUK 20130071/1 **B***Pollicariamyersii*, CUMZ collection **C***Pseudopomatiaslinanprietoae*, holotype HNHM 98835 **D, E***Pseudopomatiassophiae***D** holotype NHMUK 1910.1.21.2 and **E** CUMZ collection **F***Pupinabrachysoma*, CUMZ collection **G***Pupinamouhoti*, CUMZ collection. Photo: B. Páll-Gergely (**C**).

Lectotype NHMUK 20130071/1 (Fig. 15A) and paralectotype NHMUK 20130071/2 (1 shell).

############ Distribution.

Thailand and probably in Cambodia and Laos (Kobelt 1902a, Kongim et al. 2013).

############ Remarks.

No material of this species was found, and only the type specimens were examined.

########### Pollicaria myersii

Taxon classificationAnimaliaCycloneritidaPupinidae

(Haines, 1855)

Cyclostoma myersii Haines, 1855: 157, pl. 5, figs 9–11. Type locality: Siam [Thailand].
Pollicaria myersi [sic]: Kobelt 1902a: 290.
Pollicaria myersii : Solem 1966: 13. Kongim et al. 2013: 30, figs 2a, 4f, g, 6a.

############ Material examined.

Specimens from Ban Phone Can village, Yommalath District, Khammouan Province (Figs 15B, 18G).

############ Distribution.

Laos and Thailand (Kobelt 1902a, Solem 1966, Kongim et al. 2013).

########## *Pseudopomatias* Möllendorff, 1885

########### Pseudopomatias linanprietoae

Taxon classificationAnimaliaCycloneritidaPupinidae

Páll-Gergely, 2015

Pseudopomatias linanprietoae Páll-Gergely in Páll-Gergely et al. 2015a: 35, figs 6d, 9g. Type locality: Laos, Luang Prabang Prov., Nong Kiau [Ngoy District, Luang Phrabang Province, Laos].

############ Material examined.

Holotype HNHM 98835 (Fig. 15C).

############ Distribution.

Known only from the type locality in Laos (Páll-Gergely et al. 2015).

############ Remarks.

No material of this species was found, and only the type specimens were examined.

########### Pseudopomatias sophiae

Taxon classificationAnimaliaCycloneritidaPupinidae

Páll-Gergely, 2015

Pseudopomatias sophiae Páll-Gergely in Páll-Gergely et al. 2015a: 41, figs 6b, 9f. Type locality: Tonkin, Trinh-Thuong [Bat Xat District, Lao Cai Province, Vietnam]. Do et al. 2015: 128, fig. 7b.

############ Material examined.

Holotype NHMUK 1910.1.21.2 (Fig. 15D). Specimens from Nam Ork Roo, Ban Nathong village, Namo District, Oudomxay Province (Fig. 15E).

############ Distribution.

Vietnam (Do et al. 2015, Páll-Gergely et al. 2015).

########## *Pupina* Vignard, 1829

########### Pupina brachysoma

Taxon classificationAnimaliaCycloneritidaPupinidae

Ancey, 1904

Pupina brachysoma Ancey in Bavay and Dautzenberg 1904[1903]: 230, 231, pl. 10, figs 15, 16. Type locality: Haut Tonkin [North Vietnam]. Wood and Gallichan 2008: 31, pl. 25, figs 5, vi (label).

############ Material examined.

Specimens from Nam Ork Roo, Ban Nathong village, Namo District, Oudomxay Province (Fig. 15F).

############ Distribution.

Laos and Vietnam (Wood and Gallichan 2008).

############ Remarks.

For the correct authorship of the name, see Wood and Gallichan (2008: 31).

########### Pupina mouhoti

Taxon classificationAnimaliaCycloneritidaPupinidae

Pfeiffer, 1861

Pupina mouhoti Pfeiffer, 1861a: 196. Type locality: Camboja [Cambodia]. Saurin 1953: 113.
Pupina (Tylotoechus) mouhoti : Kobelt 1902a: 317.

############ Material examined.

Ngoy Town, Ngoy District, Luang Phrabang Province (Fig. 15G).

############ Distribution.

Cambodia and Laos (Kobelt 1902a, Saurin 1953)

########### Pupina verneaui

Taxon classificationAnimaliaCycloneritidaPupinidae

Dautzenberg & Fischer, 1906

Pupina verneaui Dautzenberg & Fischer, 1906[1905]: 440, 441, pl. 10, figs 13–15. Type locality: Ha-Giang [Ha Giang Province, Vietnam]. Do et al. 2015: 126, fig. 6c.

############ Material examined.

**Figure 16.**
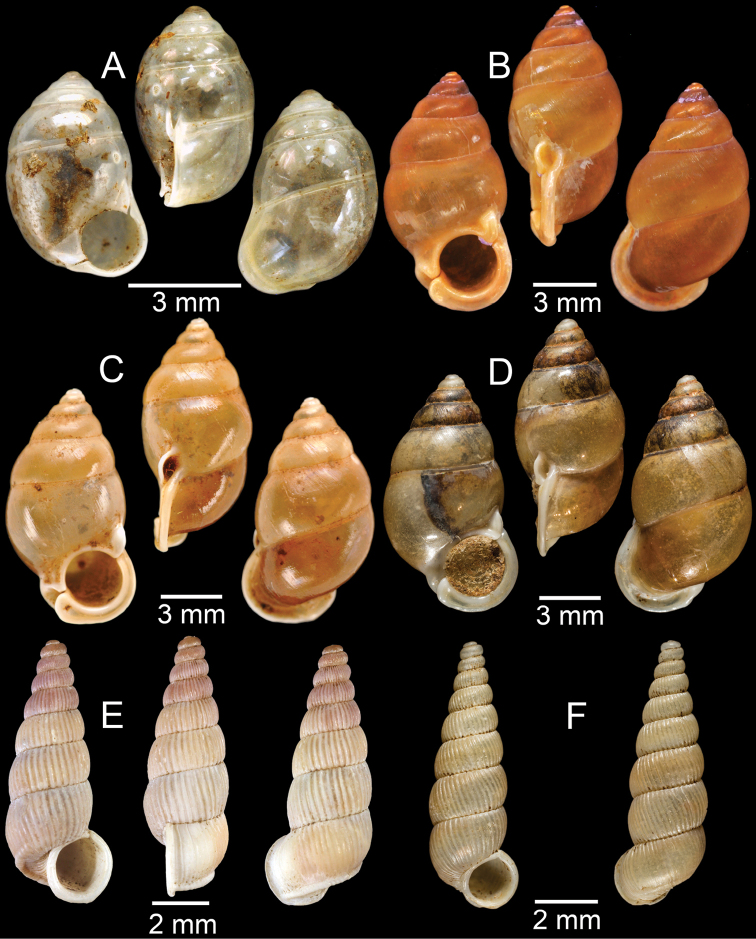
**A***Pupinaverneaui*, CUMZ collection **B, C***Pupinellafrednaggsi***B** holotype NHMUK 20170285 and **C** CUMZ collection **D***Pupinellamansuyi*, syntype MNHN-IM-2000-30756 **E***Vargapupabiheli*, holotype MNHN-IM-2012-27020 **F***Vargapupahumilis*, holotype MNHN-IM-2012-27159. Photos: B. Páll-Gergely (**E, F**).

Ban Nong Kham village, Kasy District, Vientiane Province (Fig. 16A).

############ Distribution.

Vietnam (Do et al. 2015).

########## *Pupinella* Gray, 1850

########### Pupinella frednaggsi

Taxon classificationAnimaliaCycloneritidaPupinidae

Thach & Huber, 2017

Pupinella frednaggsi Thach & Huber in Thach, 2017: 19, 20, figs 124–130. Type locality: suburb of Luang Phrabang, Central Laos [Luang Phrabang Province, Laos].

############ Material examined.

Holotype NHMUK 20170285 (Fig. 16B). Specimens from Tam Phatok Cave, Ngoy District, Luang Phrabang Province (Figs 16C, 18H).

############ Distribution.

Known only from the type locality in Laos (Thach 2017).

########### Pupinella mansuyi

Taxon classificationAnimaliaCycloneritidaPupinidae

(Dautzenberg & Fischer, 1908)

Eupupina mansuyi Dautzenberg & Fischer, 1908: 207, 208, pl. 6, figs 12–15. Type locality: Deux-Ponts; Quang-Huyen [Quang Uyen District, Cao Bang Province, Vietnam].
Pupina mansuyi : Saurin 1953: 113.
Pupinella mansuyi : Do et al. 2015: 128, fig. 7c.

############ Material examined.

Syntype MNHN-IM-2000-30756 from “Deux-Ponts” (1 shell; Fig. 16D).

############ Distribution.

Laos and Vietnam (Saurin 1953, Do et al. 2015).

############ Remarks.

No material of this species was found, and only the type specimen was examined.

########## *Vargapupa* Páll-Gergely, 2015

########### Vargapupa biheli

Taxon classificationAnimaliaCycloneritidaPupinidae

Páll-Gergely, 2015

Pseudopomatias fulvus : Saurin 1953: 113 [not Möllendorff (1901a)].
Vargapupa biheli Páll-Gergely in Páll-Gergely et al. 2015a: 42, fig. 8d. Type locality: Laos, Tran Ninh Province, Pa Hia, Pah Xieng Tong [probably refers to Ban Namthong, Longchaeng District, Xaisomboun Province, Laos].

############ Material examined.

Holotype MNHN-IM-2012-27020 (Fig. 16E).

############ Distribution.

Known only from the type locality in Laos (Páll-Gergely et al. 2015a).

############ Remarks.

No material of this species was found, and only the type specimens were examined. Páll-Gergely et al. (2015a) described this species based on Saurin’s specimens identified as “*Pseudopomatias fulvus*.” For the current interpretation of Pa Hia, see Páll-Gergely et al. (2016: 13).

########### Vargapupa humilis

Taxon classificationAnimaliaCycloneritidaPupinidae

Páll-Gergely, 2016

Vargapupa humilis Páll-Gergely, 2016: 432, 433, figs 3a–i. Type locality: Central Laos, Luang Prabang province, just northeast of Phou Khoun [Phoukhoune District, Luang Prabang Province, Laos].

############ Material examined.

Holotype MNHN-IM-2012-27159 (Fig. 16F).

############ Distribution.

Known only from the type locality in Laos (Páll-Gergely 2016).

############ Remarks.

No material of this species was found, and only the type specimens were examined.

**Figure 17.**
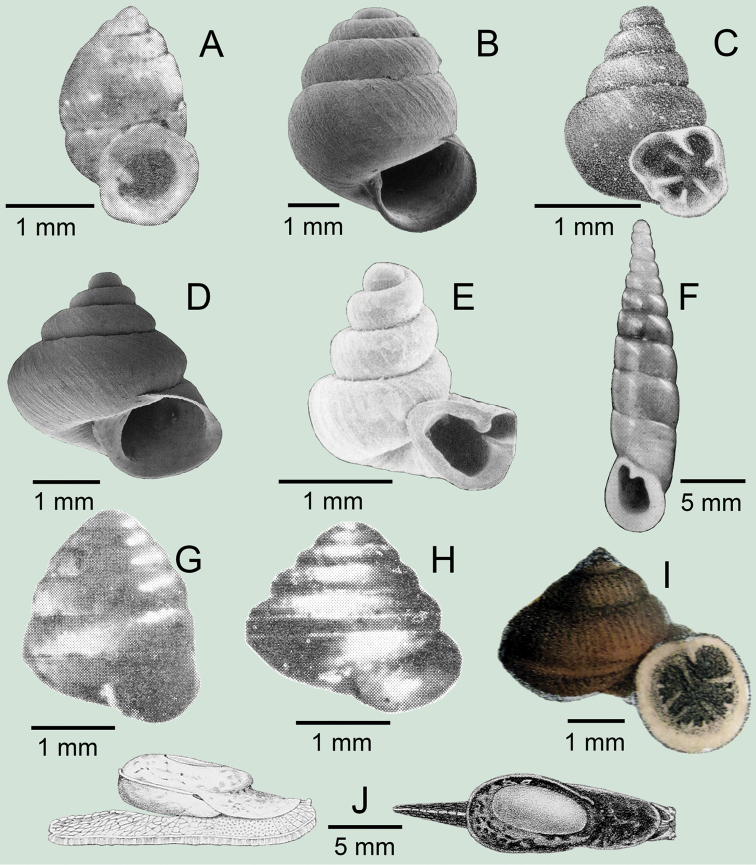
Published figures of the type specimens of **A***Diplommatinarotundata*, syntype (after Saurin (1953)) **B***Pupisomalignicola*, possible syntype (after Maassen (2000)) **C***Boysidiapaviei*, syntype (after Bavay and Dautzenberg (1912)) **D***Krobylosclerxi*, holotype RMNH 109519 (after Maassen (2008) with permission) **E***Paraboysidiawangviangensis*, holotype CUMZ-Ver 988 (after Panha et al. (2002)) **F***Oospirabolovenica*, lectotype SMF 62250 (after Zilch (1954a) with permission) **G***Kaliellamicracyna*, syntype (after Saurin (1953)) **H***Sitalatricincta*, syntype (after Saurin (1953)) **I***Gyliotrachelacrossei*, syntype (after Morlet (1887)) **J***Microparmarionandamanica*, syntype (after Collinge (1901b)).

**Figure 18.**
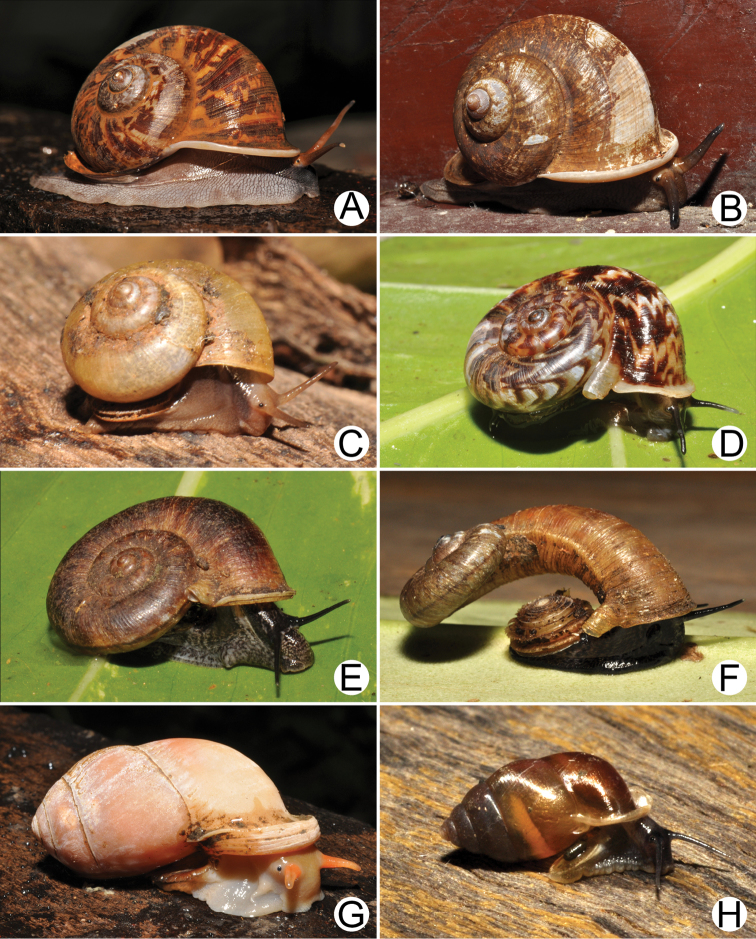
Living snails of **A***Cyclophorussiamensis***B***Cyclophorusvolvulus***C***Lagocheilusklobukowskii***D***Spiraculum* sp. **E***Rhiostomamarioni***F***Rhiostoma* sp. **G***Pollicariamyersii***H***Pupinellafrednaggsi*. All not to scale.

##### Subclass Heterobranchia

###### Infraclass Euthyneura

####### Cohort Tectipleura

######## Subcohort Panpulmonata

######### Superorder Eupulmonata

########## Order Systellommatophora [= Soleolifera]

########### Superfamily Veronicelloidea

############ Family Rathouisiidae Heude, 1885

############# *Atopos* Simroth, 1891

############## Atopos laidlawi

Taxon classificationAnimaliaSystellommatophoraRathouisiidae

Collinge, 1902

Atopos laidlawi Collinge, 1902: 90, 91, pl. 5, figs 53–55. Type locality: Ban Kong Rah, District of Gaboing [in the area of Kabang District, Yala Province, Thailand].

############### Material examined.

**Figure 19.**
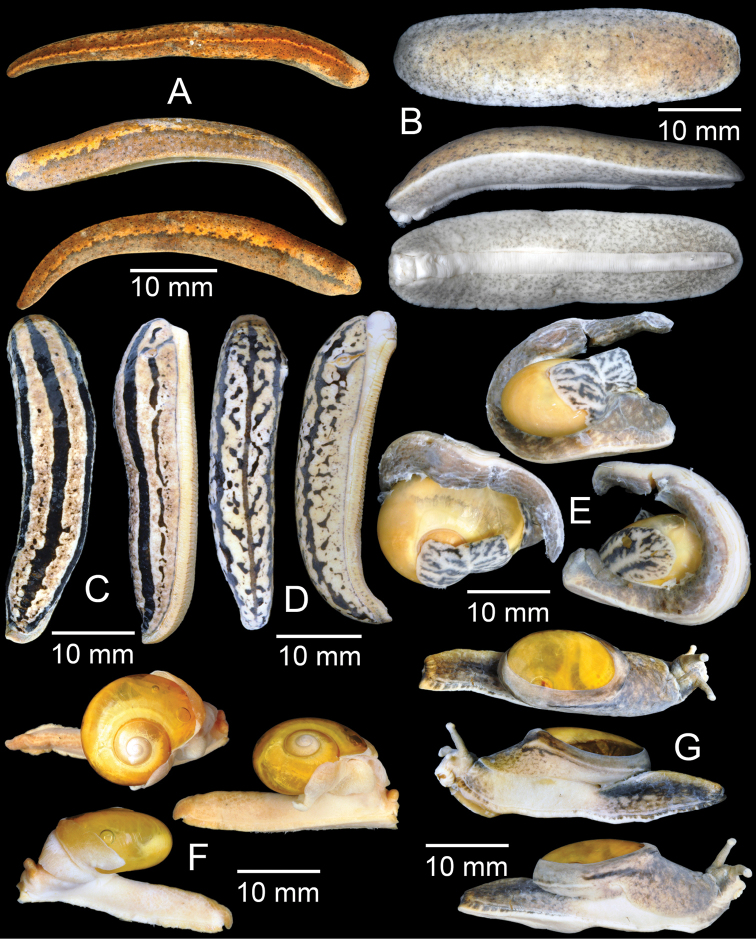
**A***Atoposlaidlawi*, CUMZ collection **B***Valigunasiamensis*, CUMZ collection **C***Meghimatiumbilineatum*, CUMZ collection **D***Meghimatiumpictum*, CUMZ collection **E***Cryptosemelus* sp., CUMZ collection **F***Durgellalibas*, CUMZ collection **G***Parmarionmartensi*, CUMZ collection.

Specimen from Ngoy Town, Ngoy District, Luang Phrabang Province (Figs 19A, 55A).

############### Distribution.

Known only from the type locality in Southern Thailand (Collinge 1902).

############ Family Veronicellidae Gray, 1840

############# *Valiguna* Grimpe & Hoffmann, 1924

############## Valiguna siamensis

Taxon classificationAnimaliaSystellommatophoraVeronicellidae

(Martens, 1867)

Vaginulus siamensis Martens, 1867: 68, pl. 5, fig. 3. Type locality: Petshaburi [Petchaburi Province, Thailand].
Semperula siamensis : Grimpe and Hoffmann 1925: 388, 390–392. Hoffmann 1925: 179–181, 256, 257. Solem 1966: 21.
Valiguna siamensis : Gomes and Thomé 2004: 595, 596.

############### Material examined.

Specimens from Phou Thevada Hotel, Paksong District, Champasak Province (Figs 19B, 55B).

############### Distribution.

Sri Lanka and Thailand (Solem 1966, Gomes and Thomé 2004).

############### Remarks.

These slugs are usually found in disturbed forests, plantations and anthropogenic habitats all over Laos and Thailand.

########## Order Stylommatophora

########### Suborder Achatinina [= “Achatinoid Clade”]

############ Superfamily Achatinoidea

############# Family Achatinidae Swainson, 1840

############## Subfamily Achatininae Swainson, 1840

############### *Lissachatina* Bequaert, 1950

################ Lissachatina fulica

Taxon classificationAnimaliaStylommatophoraAchatinidae

(Bowdich, 1822)

Achatina fulica Bowdich, 1822: pl. 13, fig. 3. Type locality: unknown. Gude 1914: 340. Maassen 2001: 78. Schileyko 2011: 8.
Achatina (Lissachatina) fulica : Bequaert 1950: 50–94.

################# Material examined.

**Figure 20.**
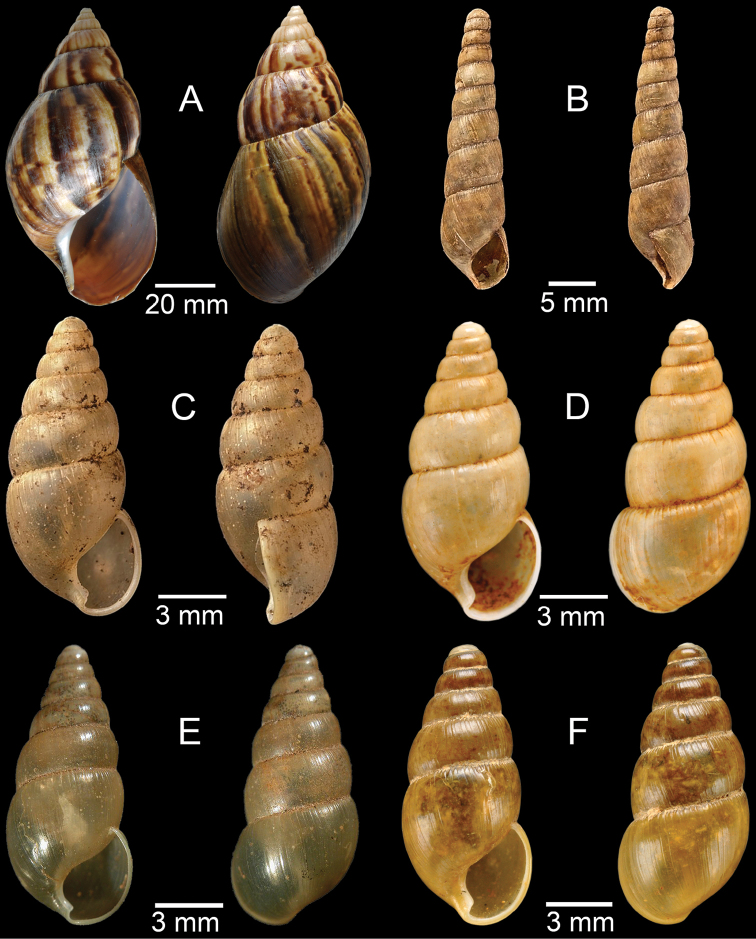
**A***Lissachatinafulica*, CUMZ collection **B***Glessulakentungensis*, syntype NHMUK 1986002 **C, D***Glessulalatestriata***C** paratype NHMUK 1926.2.3.19-20 and **D** CUMZ collection **E, F***Glessulapaviei***E** syntype MNHN-IM-2000-4668 and **F** CUMZ collection.

Specimens from Vientiane Province, Laos (Fig. 20A).

################# Distribution.

The origin of this species is probably from East Africa (Bequaert 1950). Currently it has been introduced to many tropical countries including all over Laos and Thailand.

############## Subfamily Glessulinae Godwin-Austen, 1920

############### *Glessula* Martens, 1860

################ Glessula kentungensis

Taxon classificationAnimaliaStylommatophoraAchatinidae

Godwin-Austen, 1920

Glessula kentungensis Godwin-Austen, 1920: 57, 58. Type locality: Mong Sing, Siam Boundary [Sing District, Luang Namtha Province, Laos].

################# Material examined.

Syntypes NHMUK 1986002 from “Mong Sing, Siam Boundary” (4 shells; Fig. 20B).

################# Distribution.

Known only from the type locality “Mong Sing, Siam Boundary” which is now located in Luang Namtha Province of Laos (Godwin-Austen 1920).

################# Remarks.

No material of this species was found, and only the type specimens were examined.

################ Glessula latestriata

Taxon classificationAnimaliaStylommatophoraAchatinidae

Möllendorff, 1899

Glessula latestriata Möllendorff, 1899: 166. Type locality: Kalow, Shan Staaten [Kalaw Township, Taunggyi District, Shan State, Myanmar]. Zilch 1973a: 110, pl. 5, fig. 26. Solem 1966: 93–94, pl. 2, fig. e.

################# Material examined.

Holotype SMF 145919 figured in Zilch (1973a: pl. 5, fig. 26) and paratypes SMF 227513 (2 shells), NHMUK 1926.2.3.19-20 (2 shells; Fig. 20C). Specimens from limestone hills at Ban Oudom village, Pakbeg District, Oudomxay Province (Fig. 20D).

################# Distribution.

Myanmar and Thailand (Zilch 1973a, Solem 1966).

################ Glessula paviei

Taxon classificationAnimaliaStylommatophoraAchatinidae

Morlet, 1893

Glessula paviei Morlet, 1893[1892]: 321, 322, pl. 7, figs 4, 4a, b. Type locality: Muong-Laï, dans le Laos; Laï Chan, bords de la rivière Noire, Tonkin [Muong Lai, Laos and Lai Chan on the banks of Black River, Tonkin]. Saurin 1953: 113. Schileyko 2011: 11.

################# Material examined.

Syntype MNHN-IM-2000-4668 from “Muong-Lai, dans le Laos” (1 shell; Fig. 20E). Specimens from Ban Nong Tang village, Phookood District, Xieng Khaung Province (Fig. 20F).

################# Distribution.

Laos and Vietnam (Saurin 1953, Schileyko 2011).

############## Subfamily Subulininae Fischer & Crosse, 1877

############### *Allopeas* Baker, 1935

################ Allopeas gracilis

Taxon classificationAnimaliaStylommatophoraAchatinidae

(Hutton, 1834)

Bulimus (?) gracilis (?) Hutton, 1834: 84, 85, 93. Type locality: Mirzapoor; Futtehpoor Sikra; between Agra and Neemuch [refers to the area of Uttar Pradesh and Madhya Pradesh States, India].
Lamellaxis (Allopeas) gracile [sic]: Solem 1966: 94.
Allopeas gracilis : Maassen 2001: 81, 82.
Allopeas gracile [sic]: Schileyko 2011: 9. Do and Do 2014: 452, fig. 1a. Raheem et al. 2014: 117, 118, fig. 73d–f.

################# Material examined.

**Figure 21.**
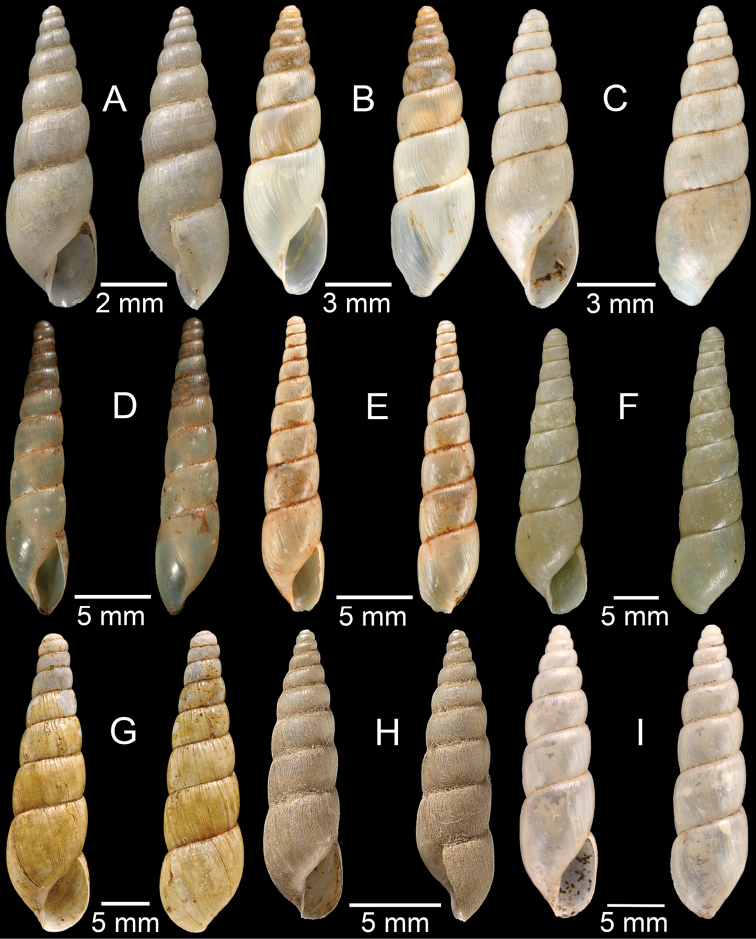
**A–C***Allopeasgracilis***A** paralectotypes NHMUK 1856.9.15.68/2-11 **B, C** CUMZ collections **D, E***Prosopeasanceyi***D** syntype of “*macilentum*” MNHN-IM-2000-4693 and **E** CUMZ collection **F, G***Prosopeasexcellens***F** syntype MNHN-IM-2000-4661 and **G** CUMZ collection **H, I***Prosopeasturricula***H** syntype NHMUK 1895.8.1.10 and **I** CUMZ collection.

Lectotype NHMUK 1856.9.15.68/1, paralectotypes 1856.9.15.68/2-11 (10 shells; Fig. 21A). Specimens from Thung Hai Hin (Plain of Jars), Phonsavanh Town, Pek District, Xieng Khaung Province (Figs 21B, C).

################# Distribution.

All over Laos, Thailand and Vietnam (Solem 1966, Schileyko 2011).

################# Remarks.

This species occurs in both natural and transformed anthropogenic habitats. This widespread and pan-tropical species has been introduced into many countries, including in greenhouses, and occurs throughout Laos and Thailand.

############### *Prosopeas* Mörch, 1876

################ Prosopeas anceyi

Taxon classificationAnimaliaStylommatophoraAchatinidae

Pilsbry, 1906

Prosopeas macilentum Ancey in Bavay and Dautzenberg 1904[1903]: 220, 221, pl. 9, figs 23, 24 [non Reeve 1849: Bulimus, pl. 79, species 586]. Type locality: Bac-Kan [Bac Kan Province, Vietnam]. Wood and Gallichan 2008: 61.
Prosopeas anceyi Pilsbry, 1906: 33, pl. 6, figs 72, 73 [new replacement name]. Schileyko 2011: 10.

################# Material examined.

Syntype of “*macilentum* Ancey, 1904” MNHN-IM-2000-4693 from “Bac-Kan, Tonkin” (1 shell; Fig. 21D). Specimens from Tam Xang Cave, Ban Nam Kha village, Kham District, Xieng Khaung Province (Fig. 21E).

################# Distribution.

Vietnam (Schileyko 2011).

################ Prosopeas excellens

Taxon classificationAnimaliaStylommatophoraAchatinidae

Bavay & Dautzenberg, 1909

Prosopeas excellens Bavay & Dautzenberg, 1909d[1908]: 247, 248. Type locality: Phong Tho [Phong Tho District, Lai Chau Province, Vietnam], Muong Bo [probably refers to the Nam Sai Commune, Sa Pa District, Lao Cai Province, Vietnam]. Bavay and Dautzenberg 1909c: 282, 283, pl. 10, figs 11, 12. Schileyko 2011: 10.

################# Material examined.

Syntype MNHN-IM-2000-4661 from “Muong-Bo et Phong-Tho” (1 shell; Fig. 21F). Specimens from Tarng Kong, Ban Phak Kard village, Pek District, Xieng Khaung Province (Figs 21G, 55C).

################# Distribution.

Vietnam (Schileyko 2011)

################ Prosopeas henrici

Taxon classificationAnimaliaStylommatophoraAchatinidae

(Ancey, 1898)

Stenogyra henrici Ancey, 1898: 134, 135, pl. 9, fig. e. Type locality: Luang-prabang [Luang Phrabang Province, Laos]. Wood and Gallichan 2008: 51.

################# Material examined.

**Figure 22.**
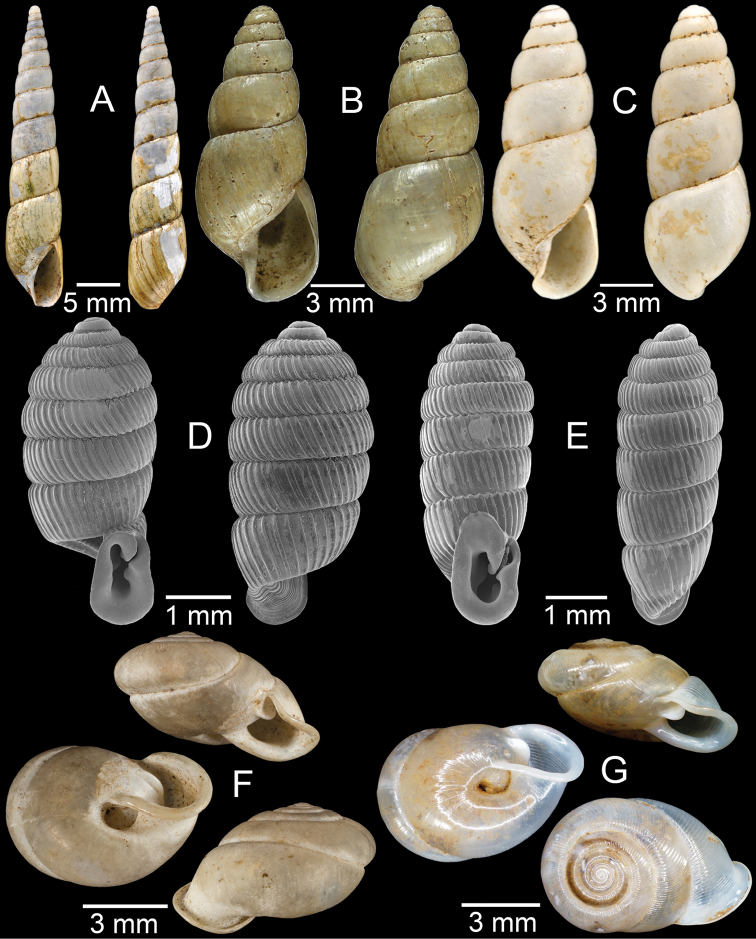
**A***Prosopeashenrici*, CUMZ collection **B, C***Prosopeasventrosulum***B** syntype MNHN-IM-2000-4666 and **C** CUMZ collection **D***Sinoenneaeuryomphala*, holotype CUMZ 7067 **E***Sinoennealizae*, specimen CUMZ 7065 **F, G***Haploptychiusblaisei***F** holotype MNHN-IM-2000-30866 and **G** specimen CUMZ 6276.

Specimens from Phou Fa Mountain, Phongsaly District, Phongsaly Province (Fig. 22A).

################# Distribution.

Known only from the type locality in Laos (Ancey 1898).

################ Prosopeas turricula

Taxon classificationAnimaliaStylommatophoraAchatinidae

(Martens, 1860)

Stenogyra turricula Martens, 1860: 9. Type locality: Siam [Thailand]. Martens 1867: 82, 83, pl. 22, fig. 7.
Paropeas turricula : Maassen 2001: 81.

################# Material examined.

Syntypes NHMUK 1895.8.1.10 from “Siam” (3 shells; Fig. 21H). Specimens from Hot Spring, Kham District, Xieng Khaung Province (Fig. 21I).

################# Distribution.

Peninsular Malaysia and Thailand (Maassen 2001).

################ Prosopeas ventrosulum

Taxon classificationAnimaliaStylommatophoraAchatinidae

Bavay & Dautzenberg, 1909

Prosopeas ventrosulum Bavay & Dautzenberg, 1909d[1908]: 248. Type locality: Phong Tho. Bavay and Dautzenberg 1909c: 283, pl. 10, figs 13, 14. Schileyko 2011: 10.

################# Material examined.

Syntype MNHN-IM-2000-4666 from “Phong-To” (1 shell; Fig. 22B). Specimens from Nam Ork Roo, Ban Nathong village, Namo District, Oudomxay Province (Fig. 22C).

################# Distribution.

Vietnam (Schileyko 2011)

############ Superfamily Streptaxoidea

############# Family Diapheridae Panha & Naggs, 2010

############## *Sinoennea* Kobelt, 1904

############### Sinoennea euryomphala

Taxon classificationAnimaliaStylommatophoraDiapheridae

Inkhavilay & Panha, 2016

Sinoennea euryomphala Inkhavilay & Panha in Inkhavilay et al. 2016b: 226–229, fig. 6d–f. Type locality: Tam Pathok Cave, Ngoi District, Luang Phrabang Province, Laos.

################ Material examined.

Holotype CUMZ 7067 (Fig. 22D).

################ Distribution.

Known only from the type locality in Laos (Inkhavilay et al. 2016b).

############### Sinoennea lizae

Taxon classificationAnimaliaStylommatophoraDiapheridae

Maassen, 2008

Sinoennea lizae Maassen, 2008: 235, figs 1–4. Type locality: Tam Khama, Ban Phou Lek, Vieng Phouka District, Luang Namtha Province, Laos. Inkhavilay et al. 2016b: 226, fig. 6a–c.

################ Material examined.

Holotype RMNH 109522 figured in Maassen (2008: figs 1–2). Specimens CUMZ 7065 from Vieng Sawang village, Vieng Phouka District, Luang Namtha Province (Fig. 22E).

################ Distribution.

Laos (Inkhavilay et al. 2016b).

############# Family Streptaxidae Gray, 1860

############## *Haploptychius* Möllendorff, 1905

############### Haploptychius blaisei

Taxon classificationAnimaliaStylommatophoraStreptaxidae

(Dautzenberg & Fischer, 1905)

Streptaxis blaisei Dautzenberg & Fischer, 1905: 86, 87, pl. 3, figs 1–4. Type locality: Ile Krieu, Tonkin [Krieu Island, Ha Long Provincial, Quang Ninh Province, Vietnam].
Haploptychius blaisei : Richardson 1988: 212. Schileyko 2011: 24. Inkhavilay et al. 2016a: 36, figs 4d–f.

################ Material examined.

Holotype MNHN-IM-2000-30866 (Fig. 22F). Specimens CUMZ 6276, 6257 from Tam Phatok, Ngoi District, Luang Phrabang Province (Fig. 22G).

################ Distribution.

Laos and Vietnam (Schileyko 2011, Inkhavilay et al. 2016a)

############### Haploptychius pellucens

Taxon classificationAnimaliaStylommatophoraStreptaxidae

(Pfeiffer, 1863)

Streptaxis pellucens Pfeiffer, 1863a[1862]: 273, pl. 36, fig. 6. Type locality: Lao Mountains, Camboja [Cambodia or Laos]. Pfeiffer 1871: 29, 30, pl. 115, figs 11, 12. Saurin 1953: 113.
Haploptychius pellucens : Inkhavilay et al. 2016a: 27–33, figs 2a, 3a–c, 7a, b, 8a–d, 9a–f, 10g.

################ Material examined.

**Figure 23.**
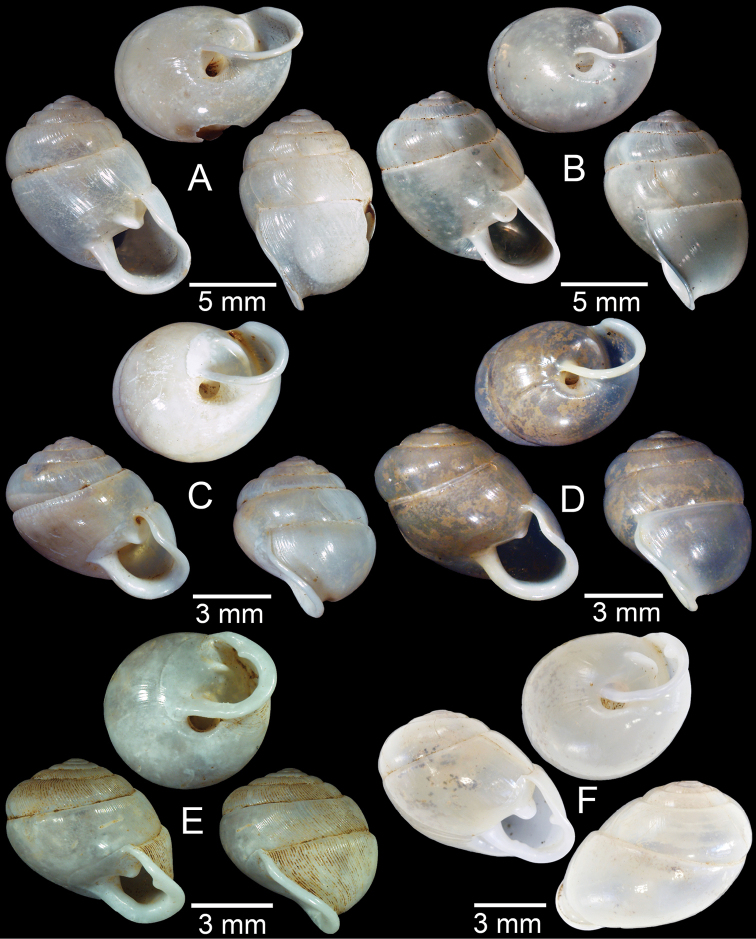
**A, B***Haploptychiuspellucens***A** lectotype NHMUK 20160249.1 and **B** specimen CUMZ 6264 **C, D***Haploptychiusporrectus***C** lectotype NHMUK 20140750.1 and **D** specimen CUMZ 6273 **E***Indoartemondiodonta*, holotype CUMZ 6289 **F***Indoartemontridens*, holotype SMF 108507/1.

Lectotype NHMUK 20160249.1 (Fig. 23A), paralectotype NHMUK 20160249.2 (2 shells). Specimens CUMZ 6264 from Ban Homexay village road to Laos-Thailand border (40 km from Ngeun Town), Ngeun District, Xayaboury Province (Fig. 23B).

################ Distribution.

Cambodia and several localities in Laos (Saurin 1953, Inkhavilay et al. 2016a).

############### Haploptychius porrectus

Taxon classificationAnimaliaStylommatophoraStreptaxidae

(Pfeiffer, 1863)

Streptaxis porrecta Pfeiffer, 1863a[1862]: 273. Type locality: Lao Mountains, Camboja [Cambodia or Laos].
Haploptychius porrectus : Inkhavilay et al. 2016a: 34, 35, figs 2b, 3d–f, 7c, d, 9g–m, 10h.

################ Material examined.

Lectotype NHMUK 20140750.1 (Fig. 23C), paralectotype NHMUK 20140750.2 (1 shell). Specimens CUMZ 6273 from Ban Nong Tang village, Phookood District, Xieng Khaung Province (Fig. 23D).

################ Distribution.

Known from several localities in Laos (Inkhavilay et al. 2016a)

############## *Indoartemon* Forcart, 1946

############### Indoartemon diodonta

Taxon classificationAnimaliaStylommatophoraStreptaxidae

Inkhavilay & Panha, 2016

Indoartemon diodonta Inkhavilay & Panha in Inkhavilay et al. 2016a: 46–49, fig. 6d–f. Type locality: Tam Xang, Thakhek District, Khammouan Province, Laos.

################ Material examined.

Holotype CUMZ 6289 (Fig. 23E).

################ Distribution.

Known only from the type locality in Laos (Inkhavilay et al. 2016a).

############### Indoartemon tridens

Taxon classificationAnimaliaStylommatophoraStreptaxidae

(Möllendorff, 1898)

Streptaxis tridens Möllendorff, 1898: 67. Type locality: Boloven [Boloven Plateau, Paksong District, Champasak Province, Laos].
Indoartemon tridens : Schileyko 2011: 23. Inkhavilay et al. 2016a: 44–46, fig. 6c.

################ Material examined.

Holotype SMF 108507 (Fig. 23F).

################ Distribution.

Known only from the type locality in Laos (Inkhavilay et al. 2016a), and possibly in Vietnam (Schileyko 2011).

################ Remarks.

No material of this species was found, and only the type specimen was examined.

############## *Perrottetia* Kobelt, 1905

############### Perrottetia aquilonaria

Taxon classificationAnimaliaStylommatophoraStreptaxidae

Siriboon & Panha, 2013

Perrottetia aquilonaria Siriboon & Panha in Siriboon et al. 2013: 50–52, figs 3d–h, 4d–f, 5h–m, 6b. Type locality: Wat Tam Pha Plong, Chiangdao District, Chiangmai Province, Thailand. Inkhavilay et al. 2016a: 40, fig. 5e.

################ Material examined.

**Figure 24.**
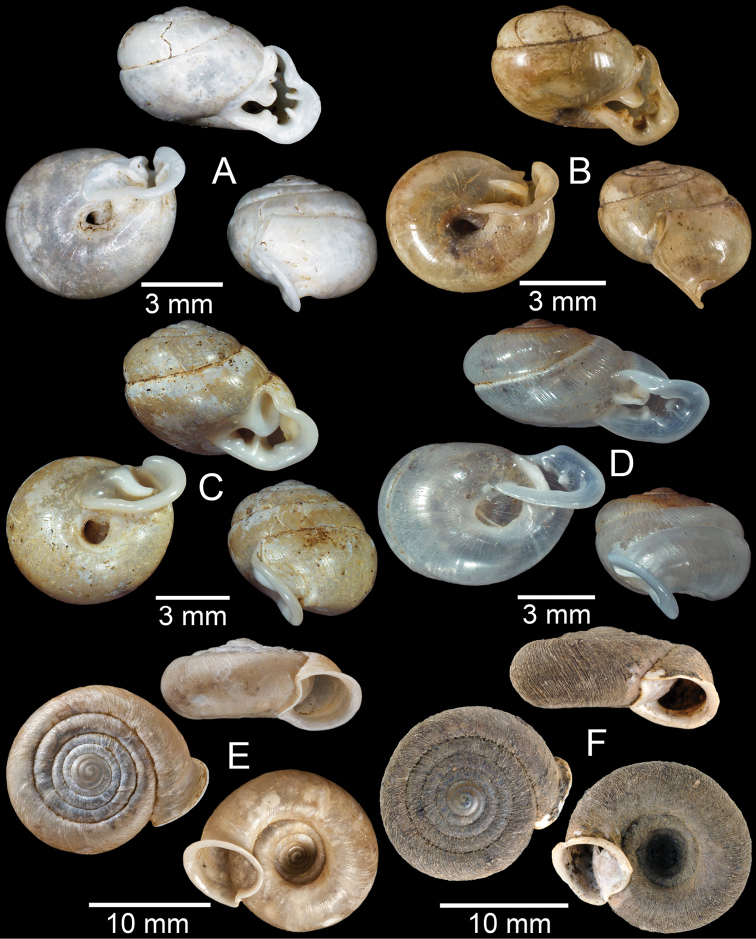
**A***Perrottetiaaquilonaria*, specimen CUMZ 6278 **B***Perrottetiadugasti*, syntype MNHN-IM-2000-30867 **C***Perrottetiamegadentata*, holotype CUMZ 6286 **D***Perrottetiaunidentata*, holotype CUMZ 6281 **E***Gudeodiscusmessageriraheemi*, holotype NHMUK 20110370.1 **F***Gudeodiscus* sp., CUMZ collection.

Holotype CUMZ 5003 figured in Siriboon et al. (2013: fig. 3d). Specimens CUMZ 6278 from Ban Namone village, Xayaboury District, Xayaboury Province (Fig. 24A).

################ Distribution.

Laos and Thailand (Siriboon et al. 2013, Inkhavilay et al. 2016a).

################ Remarks.

Both species names “*aquilonaria*” and “*aquilonaris*” were presented in the original description (Siriboon et al. 2013). However, the species name “*aquilonaria*” was selected as the correct original spelling by the original authors as the first revisers in Inkhavilay et al. 2016a (ICZN 1999: Art. 24.2.4.).

############### Perrottetia dugasti

Taxon classificationAnimaliaStylommatophoraStreptaxidae

(Morlet, 1892)

Streptaxis dugasti Morlet, 1892b: 82. Type locality: Laï-Chau, sur les bords de la Rivière Noire, Tonkin [on the banks of the Black River, Lai Chau Province, Vietnam]. Morlet 1893: 315, 316, pl. 7, figs 5, 5a, b.
Perrottetia dugasti : Schileyko 2011: 23. Inkhavilay et al. 2016a: 38, fig. 5a.

################ Material examined.

Lectotype MNHN-IM-2000-30867 (Fig. 24B).

################ Distribution.

Laos and Vietnam (Schileyko 2011).

################ Remarks.

No material of this species was found, and only the type specimen was examined.

############### Perrottetia megadentata

Taxon classificationAnimaliaStylommatophoraStreptaxidae

Inkhavilay & Panha, 2016

Perrottetia megadentata Inkhavilay & Panha in Inkhavilay et al. 2016a: 42–44, fig. 6a, b. Type locality: Limestone outcrop at Ban Phone Can, Yommalat District, Khammouan Province, Laos.

################ Material examined.

Holotype CUMZ 6286 (Fig. 24C).

################ Distribution.

Known only from the type locality in Laos (Inkhavilay et al. 2016a).

############### Perrottetia unidentata

Taxon classificationAnimaliaStylommatophoraStreptaxidae

Inkhavilay & Panha, 2016

Perrottetia unidentata Inkhavilay & Panha in Inkhavilay et al. 2016a: 40–42, figs 5f–i, 7e, f, 10a–f, i. Type locality: limestone outcrop at Ban Nawit, Viengxay District, Houaphanh Province, Laos.

################ Material examined.

Holotype CUMZ 6281 (Fig. 24D).

################ Distribution.

Known only from the type locality in Laos (Inkhavilay et al. 2016a).

########## Suborder Helicina [= “Non-Achatinoid Clade”]

########### Superfamily Plectopyloidea

############ Family Plectopylidae Möllendorff, 1898

############# *Gudeodiscus* Páll-Gergely, 2013

############## Gudeodiscus messageri raheemi

Taxon classificationAnimaliaStylommatophoraPlectopylidae

Páll-Gergely & Hunyadi, 2015

Gudeodiscus (Gudeodiscus) messageriraheemi Páll-Gergely & Hunyadi in Páll-Gergely et al. 2015b: 38–41, figs 5d, e, 10a, 12r–v, 20, 28e, 29f, g, 31b, 35d–f. Type locality: Thanh Hoa Province, Cam Thuy District, Fish Stream, Vietnam. Páll-Gergely et al. 2016: 3–6, figs 1c, 2b, 5g–h, 6, 9d–g.

############### Material examined.

Holotype NHMUK 20110370.1 (Fig. 24E).

############### Distribution.

Laos and Vietnam (Páll-Gergely et al. 2015b, 2016).

############### Remarks.

No material of this species was found, and only the type specimens were examined.

############## Gudeodiscus

Taxon classificationAnimaliaStylommatophoraPlectopylidae

sp.

############### Material examined.

Specimens from Ban Naweed village, Viengxay District, Houaphanh Province (Figs 24F, 55D).

############### Remarks.

This population is very close to *Gudeodiscusmessageri*, but slightly differs in having thicken periostracum. The ribbed protoconch indicates it is a member of the genus *Gudeodiscus* (Páll-Gergely pers. comm.).

############# *Hunyadiscus* Páll-Gergely, 2016

############## Hunyadiscus saurini

Taxon classificationAnimaliaStylommatophoraPlectopylidae

Páll-Gergely, 2016

Plectopylis laomontana : Saurin 1953: 113 [not Pfeiffer (1863a)].
Hunyadiscus saurini Páll-Gergely in Páll-Gergely et al. 2016: 11–13, figs 3a, 4c, 5a, b, 11a. Type locality: Laos, Pa Hia (Ancienne Province Tran Ninh) [probably refers to Ban Namthong, Longchaeng District, Xaisomboun Province, Laos].

############### Material examined.

**Figure 25.**
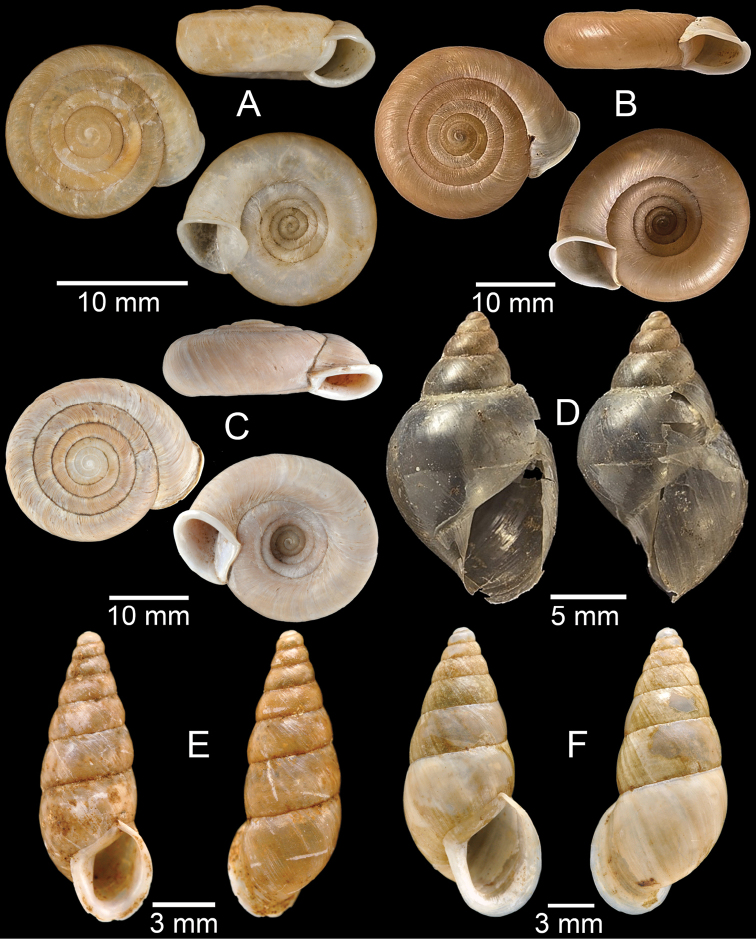
**A***Hunyadiscussaurini*, holotype MNHN-IM-2000-24947 **B, C***Naggsialaomontana***B** syntype NHMUK 2013004 and **C** CUMZ collection **D***Amimopinasubangulata*, lectotype NHMUK 1986166 **E***Apoecuscorti*, CUMZ collection **F***Apoecusmacrostoma*, CUMZ collection.

Holotype MNHN-IM-2000-24947 (Fig. 25A).

############### Distribution.

Laos (Saurin 1953, Páll-Gergely et al. 2016).

############### Remarks.

No material of this species was found, and only the type specimens were examined. Páll-Gergely et al. (2016) described this genus and species based on Saurin’s specimens identified as “*Plectopylis laomontana*”. For the current interpretation of Pa Hia, see Páll-Gergely et al. (2016: 13).

############# *Naggsia* Páll-Gergely & Muratov, 2016

############## Naggsia laomontana

Taxon classificationAnimaliaStylommatophoraPlectopylidae

(Pfeiffer, 1863)

Helix laomontana Pfeiffer, 1863a[1862]: 272, pl. 36, figs 9, 10. Type locality: Lao Mountains, Camboja [Cambodia or Laos]. Pfeiffer 1863b: 216, pl. 57, figs 7–9.
Naggsia laomontana : Páll-Gergely et al. 2016: 14–23, figs 1a, b, 2a, 4d, 5e, f, 7, 8, 9a–c, 10.

############### Material examined.

Syntypes NHMUK 2013004 from “Lao Mountains, Camboja” (3 shells; Fig. 25B). Specimens from Khaungsi waterfall, Luang Phrabang District, Luang Phrabang Province (Fig. 25C).

############### Distribution.

Known from several localities from Luang Phrabang Province, Laos (Páll-Gergely et al. 2016).

########## Infraorder Succineoidei [= Elasmognatha]

########### Superfamily Succineoidea

############ Family Succineidae Beck, 1837

############# *Succinea* Draparnaud, 1801

############## Succenia

Taxon classificationAnimaliaStylommatophoraSuccineidae

sp.

############### Material examined.

Specimen from pomelo plantation near Ngoi District, Luang Phrabang Province (Fig. 55E).

############### Remarks.

A small specimen was found on pomelo (*Citrusmaxima* Merr) leaves.

########## Infraorder Pupilloidei [= Orthurethra]

########### Superfamily Pupilloidea

############ Family Cerastidae Wenz, 1923

############# *Amimopina* Solem, 1964

############## Amimopina subangulata

Taxon classificationAnimaliaStylommatophoraCerastidae

(Pfeiffer, 1863)

Bulimus subangulatus Pfeiffer, 1863a[1862]: 274, 275. Type locality: Lao Mountains, Camboja [Cambodia or Laos].
Amimopina subangulatus [sic]: Sutcharit et al. 2010: 255, 256, figs 1e–g, i, 2c, 3e–h.

############### Material examined.

Lectotype NHMUK 1986166 (Fig. 25D).

############### Distribution.

Thailand and possibly in Cambodia or Laos (Pfeiffer 1863a, Sutcharit et al. 2010).

############### Remarks.

No material of this species was found, and only the type specimen was examined.

############ Family Enidae Woodward, 1903

############# Apoecus

Taxon classificationAnimaliaStylommatophoraEnidae

Kobelt, 1902

############## Remarks.

All enid species from Southeast Asia have recently been assigned to the genus *Coccoderma* Möllendorff, 1901 (Schileyko 2011, Köhler et al. 2017). However, this generic name is preoccupied by *Coccoderma* Zittel, 1887 (a fossil fish) and thus is not available. Here we provisionally assigned the Laotian species to the genus *Apoecus* Kobelt, 1902 (type species *Bulimuscolonus* Möllendorff, 1895 from New Guinea) which has the closest distribution range to the taxa studied (Köhler et al. 2017). However, additional anatomical and molecular studies are required to confirm the taxonomic position.

############# Apoecus corti

Taxon classificationAnimaliaStylommatophoraEnidae

(Bavay & Dautzenberg, 1909)

Helix (Buliminopsis ?) corti Bavay & Dautzenberg, 1909d[1908]: 245. Type locality: Ban-Lao [Ban Lao in Muong Bum Commune, Thuan Chau District, Son La Province, Vietnam]. Bavay and Dautzenberg 1909b: 204, 205, pl. 8, figs 23, 24.
Coccoderma (?) corti: Schileyko 2011: 5.

############## Material examined.

Specimen from Ban Nong Tang village, Phookood District, Xieng Khaung Province (Fig. 25E).

############## Distribution.

Laos and Vietnam (Schileyko 2011).

############# Apoecus macrostoma

Taxon classificationAnimaliaStylommatophoraEnidae

(Bavay & Dautzenberg, 1912)

Buliminus macrostoma Bavay & Dautzenberg, 1912: 25, 26, pl. 4, figs 11–13. Type locality: Muong-Hum [Muong Hum Commune, Bat Xat District, Lao Cai Province, Vietnam].
Ena macrostoma : Saurin 1953: 113.
Coccoderma macrostoma : Schileyko 2011: 6.

############## Material examined.

Specimen from Phou Thaleang Bio-Diversity Conservation Area, Boun Neua District, Phongsaly Province (Fig. 25F).

############## Distribution.

Laos and Vietnam (Saurin 1953, Schileyko 2011).

############ Family Valloniidae Morse, 1864

############# *Pupisoma* Stoliczka, 1873

############## Pupisoma lignicola

Taxon classificationAnimaliaStylommatophoraValloniidae

(Stoliczka, 1871)

Pupa lignicola Stoliczka, 1871: 171, 172, pl. 7, fig. 3. Type locality: Moulmein, provincia Tenasserim [Mawlamyine Township, Mawlamyine District, Mon State]. Maassen 2000: 142, fig. 8.
Pupisoma lignicola : Gude 1914: 34, 35. Pilsbry 1920: 23, 24, pl. 2, figs 7, 10. Vermeulen and Raven 1998: 274, fig. 1.

############### Distribution.

Widely distributed from India, Indonesia, Laos, Malaysia and Myanmar (Vermeulen and Raven 1998).

############### Remarks.

No material of this species was found and the possible syntype specimen was figured in Maassen (2000: fig. 8; see Fig. 17B).

############ Family Vertiginidae Fitzinger, 1833

############# *Angustopila* Jochum, Slapnik & Páll-Gergely, 2014

############## Angustopila singuladentis

Taxon classificationAnimaliaStylommatophoraVertiginidae

Inkhavilay & Panha, 2016

Angustopila singuladentis Inkhavilay & Panha in Inkhavilay et al. 2016b: 224–226, figs 5a–c. Type locality: Tam Xang Lod Cave, Viengxay District, Houaphanh Province, Laos.

############### Material examined.

**Figure 26.**
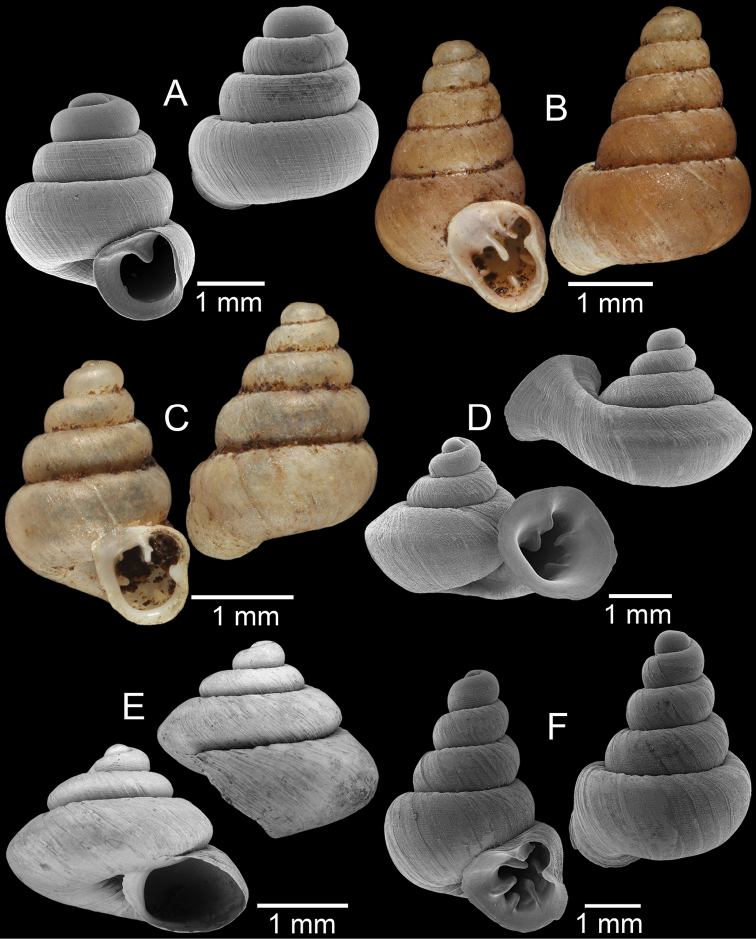
**A***Angustopilasinguladentis*, holotype CUMZ 7036 **B***Boysidianovemdentata*, syntype MNHN-IM-2000-33881 **C***Boysidiapahpetensis*, syntype MNHN-IM-2000-33880 **D***Gyliotrachelaplesiolopa*, holotype CUMZ 7061 **E***Krobyloslaosensis*, holotype MNHN-IM-2000-31746 **F***Paraboysidiaanguloobtusa*, holotype CUMZ 7057. Photo: B. Páll-Gergely (**E**).

Holotype CUMZ 7036 (Fig. 26A).

############### Distribution.

Known only from the type locality in Laos (Inkhavilay et al. 2016b).

############# *Boysidia* Ancey, 1881

############## Boysidia novemdentata

Taxon classificationAnimaliaStylommatophoraVertiginidae

Saurin, 1953

Boysidia novemdentata Saurin, 1953: 115, 116, fig. 1, and pl. 4, fig. 4a–c. Type locality: environs du village méo de Pah Hia, à 100 kilomètres au Sud de Xieng-Khouang, chef-lieu de la province du Tran Ninh, Laos [probably refers to Ban Namthong, Longchaeng District, Xaisomboun Province, Laos].

############### Material examined.

Syntype MNHN-IM-2000-33881 from “Pah Hia” (1 shell; Fig. 26B).

############### Distribution.

Known only from the type locality in Laos (Saurin 1953).

############### Remarks.

No material of this species was found, and only the type specimen was examined. For the current interpretation of Pa Hia, see Páll-Gergely et al. (2016: 13).

############## Boysidia pahpetensis

Taxon classificationAnimaliaStylommatophoraVertiginidae

Saurin, 1953

Boysidia pahpetensis Saurin, 1953: 116, fig. 2, and pl. 4, fig. 5a–c. Type locality: environs du village méo de Pah Hia, à 100 kilomètres au Sud de Xieng-Khouang, chef-lieu de la province du Tran Ninh, Laos [probably refers to Ban Namthong, Longchaeng District, Xaisomboun Province, Laos].

############### Material examined.

Syntype MNHN-IM-2000-33880 from “Pah Hia” (1 shell; Fig. 26C).

############### Distribution.

Known only from the type locality in Laos (Saurin 1953).

############### Remarks.

No material of this species was found, and only the type specimen was examined. For the current interpretation of Pa Hia, see Páll-Gergely et al. (2016: 13).

############## Boysidia paviei

Taxon classificationAnimaliaStylommatophoraVertiginidae

Bavay & Dautzenberg, 1912

Boysidia paviei Bavay & Dautzenberg, 1912: 20, 21, pl. 3, figs 4–6. Type locality: Pac-Kha [Pa Kha in Long Luong Commune, Van Ho District, Son La Province, Vietnam]; Long-Ping [Lung Phinh Commune, Bac Ha District, Lao Cai Province, Vietnam]. Saurin 1953: 113.
Boysidia (Paraboysidia) paviei : Schileyko 2011: 2.

############### Distribution.

Laos and Vietnam (Saurin 1953, Schileyko 2011).

############### Remarks.

No material of this species was found and the type specimen could not be traced. This species was figured in Bavay and Dautzenberg (1912: pl. 3, fig. 4; see Fig. 17C).

############# *Gyliotrachela* Tomlin, 1930

############## Gyliotrachela plesiolopa

Taxon classificationAnimaliaStylommatophoraVertiginidae

Inkhavilay & Panha, 2016

Gyliotrachela plesiolopa Inkhavilay & Panha in Inkhavilay et al. 2016b: 222, figs 3d–f, 4d. Type locality: limestone outcrop at Naweed village, Viengxay District, Houaphanh Province, Laos.

############### Material examined.

Holotype CUMZ 7061 (Fig. 26D).

############### Distribution.

Known only from the type locality in Laos (Inkhavilay et al. 2016b).

############# *Krobylos* Panha & Burch, 1999

############## Krobylos clerxi

Taxon classificationAnimaliaStylommatophoraVertiginidae

Maassen, 2008

Krobylos clerxi Maassen, 2008: 239, figs 7–10. Type locality: Tam Khama, Ban Phou Lek, Vieng Phouka District, Luang Namtha Province, Laos.

############### Material examined.

Holotype RMNH 109519.

############### Distribution.

Known only from the type locality in Laos (Maassen 2008).

############### Remarks.

No material of this species was found. This species was figured in Maassen (2008: fig. 7, see Fig. 17D).

############## Krobylos laosensis

Taxon classificationAnimaliaStylommatophoraVertiginidae

(Saurin, 1953)

Pyramidula laosensis Saurin, 1953: 119, pl. 4, fig. 11a–c. Type locality: environs du village méo de Pah Hia, à 100 kilomètres au Sud de Xieng-Khouang, chef-lieu de la province du Tran Ninh, Laos [probably refers to Ban Namthong, Longchaeng District, Xaisomboun Province, Laos].
Krobylos laosensis : Páll-Gergely et al. 2015c: 31 (abstract), 55.

############### Material examined.

Holotype MNHN-IM-2000-31746 (Fig. 26E).

############### Distribution.

Known only from the type locality in Laos (Saurin 1953).

############### Remarks.

No material of this species was found, and only the type specimen was examined. Páll-Gergely et al. (2015c: 31, 55) noted that this species shows a protruding last whorl, narrow umbilicus and simple apertural lip, and suggested that this species should be placed into the genus *Krobylos*. For the current interpretation of Pa Hia, see Páll-Gergely et al. (2016: 13).

############# *Paraboysidia* Pilsbry, 1917

############## Paraboysidia anguloobtusa

Taxon classificationAnimaliaStylommatophoraVertiginidae

Inkhavilay & Panha, 2016

Paraboysidia anguloobtusus Inkhavilay & Panha in Inkhavilay et al. 2016b: 215–217, figs 2d–f, 4b. Type locality: limestone wall outside of Tam Kao Rao Cave, Vieng Phouka District, Luang Namtha Province, Laos.

############### Material examined.

Holotype CUMZ 7057 (Fig. 26F).

############### Distribution.

Known only from the type locality in Laos (Inkhavilay et al. 2016b).

############### Remarks.

The gender agreement of specific epithet was modified (ICZN 1999: Art. 34.2).

############## Paraboysidia gittenbergeri

Taxon classificationAnimaliaStylommatophoraVertiginidae

Maassen, 2008

Paraboysidia gittenbergeri Maassen, 2008: 237–239, figs 5, 6. Type locality: Tam Khama, Ban Phou Lek, Vieng Phouka District, Luang Namtha Province, Laos. Inkhavilay et al. 2016b: 215, figs 2a–c, 4a.

############### Material examined.

**Figure 27.**
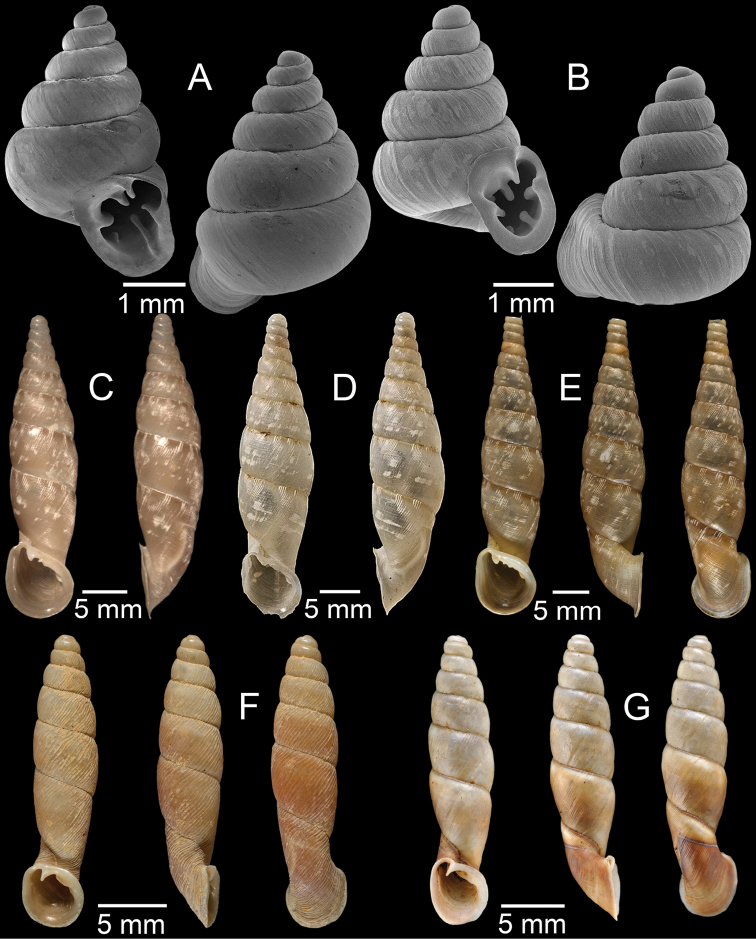
**A***Paraboysidiagittenbergeri*, specimen CUMZ 7055 **B***Paraboysidiaparalella*, holotype CUMZ 7059 **C***Garnieriamouhotimoellendorffi*, holotype SMF 32039 **D, E***Garnieriamouhotimouhoti***D** paralectotypes of “*mouhoti*” NHMUK 20010206/2-3 and **E** syntype of “*massiei*” MNHN-IM-2000-2509 **F, G***Neniaucheniaamoena***F** holotype MNHN-IM-2000-2410 and **G** CUMZ collection.

Holotype RMNH 109521, paratype RMNH 109523 (1 shell) figured in Maassen (2008: figs 5, 6). Specimen CUMZ 7055 from Viengsawang village, Vieng Phouka District, Luang Namtha Province (Fig. 27A).

############### Distribution.

Known from the type locality in Laos (Inkhavilay et al. 2016b). The recent collection was from a limestone outcrop near the type locality.

############## Paraboysidia paralella

Taxon classificationAnimaliaStylommatophoraVertiginidae

Inkhavilay & Panha, 2016

Paraboysidia paralella Inkhavilay & Panha in Inkhavilay et al. 2016b: 220, figs 3a–c, 4c. Type locality: limestone wall near the entrance of Tam Kao Rao Cave, Vieng Phouka District, Luang Namtha Province, Laos.

############### Material examined.

Holotype CUMZ 7059 (Fig. 27B).

############### Distribution.

Known only from the type locality in Laos (Inkhavilay et al. 2016b).

############## Paraboysidia wangviangensis

Taxon classificationAnimaliaStylommatophoraVertiginidae

Panha & Tongkerd, 2002

Paraboysidia wangviangensis Panha & Tongkerd in Panha et al. 2002: 123–128, figs 2–3. Type locality: Tam Chang Cave, Wangviang, Laos [limestones in Vangvieng District, Vientiane Province, Laos].

############### Material examined.

Holotype CUMZ, Ver 988 and paratypes CUMZ, Ver 089 (12 shells).

############### Distribution.

Known only from the type locality in Laos (Panha et al. 2002).

############### Remarks.

No material of this species was found. This species was figured in Panha et al. (2002: fig. 2a, see Fig. 17E).

########## Infraorder Clausilioidei

########### Superfamily Clausilioidea

############ Family Clausiliidae Gray, 1855

############# Subfamily Garnieriinae Boettger, 1926

############## *Garnieria* Bourguignat, 1877

############### Garnieria mouhoti moellendorffi

Taxon classificationAnimaliaStylommatophoraClausiliidae

Nordsieck, 2002

Garnieria mouhoti moellendorffi Nordsieck, 2002: 8, 9, fig. 2. Type locality: Laos, Luang Prabang [Luang Phrabang Province, Laos]. Nordsieck 2007: 37.

################ Material examined.

Holotype SMF 32039 (Fig. 27C) and paratypes SMF 32041 (2 shells), SMF 32042 (3 shells).

################ Distribution.

Laos (Nordsieck 2002, 2007).

################ Remarks.

No material of this species was found, and only the type specimens were examined.

############### Garnieria mouhoti mouhoti

Taxon classificationAnimaliaStylommatophoraClausiliidae

(Pfeiffer, 1863)

Clausilia mouhoti Pfeiffer, 1863a[1862]: 275, pl. 36, fig. 5. Type locality: Lao Mountains, Camboja [Cambodia or Laos].
Clausilia (Phaedusa?) massiei Morlet, 1892b: 83. Type locality: Luang Prabang, dans le Laos [Luang Phrabang Province, Laos]. Morlet 1893[1892]: 318, 319, pl. 7, figs 3, 3a, b.
Garnieria massiei : Nordsieck 2002: 6.
Garnieria mouhoti : Nordsieck 2002: 6, fig. 1. Nordsieck 2007: 37, 73, 183, pl. 6, fig. 2. Páll-Gergely and Szekeres 2017: 509–512, figs 3a, c, 4a, b, e–g, 5.

################ Material examined.

Lectotype of “*mouhoti* Pfeiffer, 1863” NHMUK 20010206/1 and paralectotypes NHMUK 20010206/2-3 (2 shells; Fig. 27D). Syntype of “*massiei* Morlet, 1892” MNHN-IM-2000-2509 from “Luang-Prabang” (1 shell; Fig. 27E).

################ Distribution.

Laos (Nordsieck 2002, 2007).

################ Remarks.

No material of this species was found, and only the type specimens were examined. Nordsieck (2002) and Páll-Gergely and Szekeres (2017) recognised “*Clausilia massiei* Morlet, 1892” as a junior synonym of this species.

############### Garnieria saurini

Taxon classificationAnimaliaStylommatophoraClausiliidae

Nordsieck, 2002

Garnieria saurini Nordsieck, 2002: 9, 10, fig. 3. Type locality: Laos, Pah Xieng Tong, Pah Hia [probably refers to Ban Namthong, Longchaeng District, Xaisomboun Province, Laos]. Nordsieck 2007: 37. Páll-Gergely and Szekeres 2017: 512.

################ Material examined.

**Figure 28.**
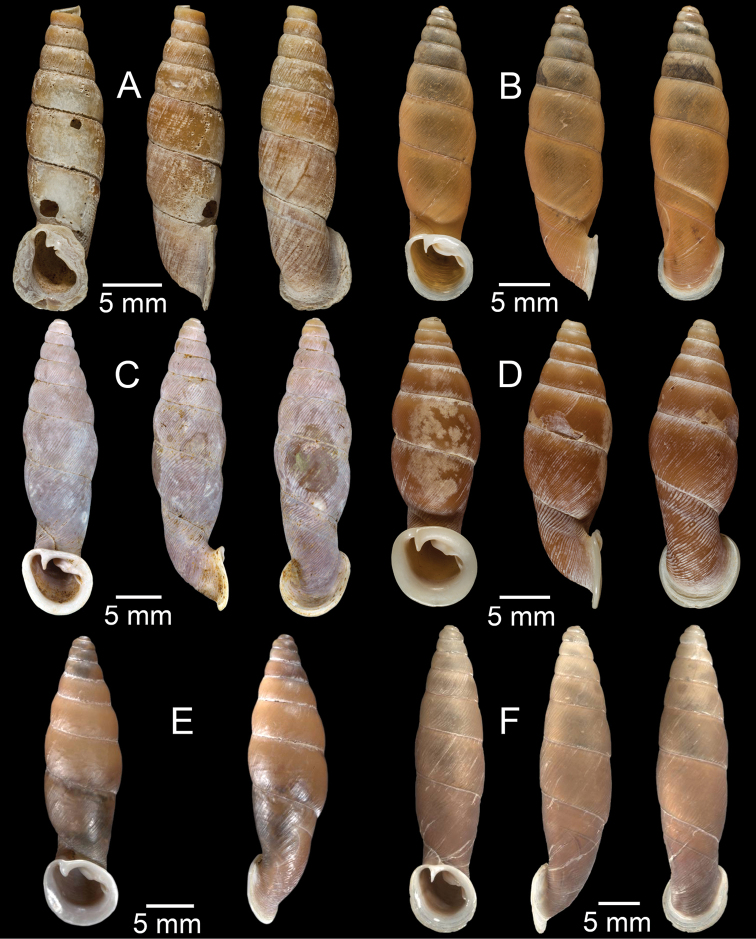
**A***Garnieriasaurini*, holotype MNHN-IM-2000-2672 **B, C***Neniaucheniadautzenbergidautzenbergi***B** syntype MNHN-IM-2000-2432 and **C** CUMZ collection **D***Neniaucheniadautzenbergidecollata*, holotype MNHN-IM-2000-2433 **E***Neniaucheniarugifera*, lectotype SMF 32015 **F***Neniaucheniatonkinensis*, holotype SMF 331370.

Holotype MNHN-IM-2000-2672 (Fig. 28A).

################ Distribution.

Laos (Nordsieck 2002, 2007).

################ Remarks.

No material of this species was found, and only the type specimen was examined. For the current interpretation of Pa Hia, see Páll-Gergely et al. (2016: 13).

############## *Neniauchenia* Nordsieck, 2002

############### Neniauchenia amoena

Taxon classificationAnimaliaStylommatophoraClausiliidae

(Nordsieck, 2002)

Tropidauchenia (Neniauchenia) amoena Nordsieck, 2002: 10, 11, fig. 4. Type locality: Laos, Phou Tiou [Phou Tiou hill near Ban Nam San Noi village, Viengkham District, Vientiane Province, Laos].
Neniauchenia amoena : Nordsieck 2007: 37.
Grandinenia amoena : Páll-Gergely and Szekeres 2017: 513.

################ Material examined.

Holotype MNHN-IM-2000-2410 (Fig. 27F). Specimens from Ban Na Phong village, Pakkading District, Bolikhamxay Province (Fig. 27G).

################ Distribution.

Known from several localities in Laos (Nordsieck 2002, Páll-Gergely and Szekeres 2017).

############### Neniauchenia dautzenbergi dautzenbergi

Taxon classificationAnimaliaStylommatophoraClausiliidae

(Morlet, 1893)

Clausilia dautzenbergi Morlet, 1893[1892]: 320, 321, pl. 7, figs 2, 2a, b. Type locality: Kham-Keute, dans le Laos [Khamkeut District, Bolikhamxay Province, Laos].
Neniauchenia dautzenbergi : Nordsieck 2007: 37.
Grandinenia dautzenbergi : Páll-Gergely and Szekeres 2017: 513–515, fig. 6a.

################ Material examined.

Syntypes MNHN-IM-2000-2432 from “Kham-Keute” (2 shells; Fig. 28B). Specimen from Tam Narng Lod Cave, Ban Na Can village, Yommalath District, Khammouan Province (Fig. 28C).

################ Distribution.

Known from several localities in Laos (Páll-Gergely and Szekeres 2017).

############### Neniauchenia dautzenbergi decollata

Taxon classificationAnimaliaStylommatophoraClausiliidae

(Nordsieck, 2002)

Tropidauchenia (Neniauchenia) dautzenbergidecollata Nordsieck, 2002: 11, 16, fig. 5. Type locality: Laos, B. (= Ban) Na Ka Yak (Nhoum = Ngum?) [probably refers to Ban Me Nhoum village, Khamkeut District, Bolikhamxay Province, Laos].
Neniauchenia dautzenbergi decollata : Nordsieck 2007: 37.

################ Material examined.

Holotype MNHN-IM-2000-2433 (Fig. 28D).

################ Distribution.

Known only from the type locality in Laos (Nordsieck 2002).

################ Remarks.

No material of this species was found, and only the type specimens were examined. Páll-Gergely and Szekeres (2017: 515) questioned the status of this subspecies as its slight difference could also be seen in the specimens from Thakhek District.

############### Neniauchenia rugifera

Taxon classificationAnimaliaStylommatophoraClausiliidae

(Möllendorff, 1898)

Clausilia (Garnieria) rugifera Möllendorff, 1898: 76, 77. Type locality: Boloven [Boloven Plateau, Paksong District, Champasak Province, Laos].
Neniauchenia rugifera : Nordsieck 2007: 37, 183, pl. 6, fig. 1. Schileyko 2011: 20.

################ Material examined.

Lectotype SMF 32015 (Fig. 28E) and paratypes SMF 32016 (2 shells).

################ Distribution.

Laos and possibly in Vietnam (Nordsieck 2007, Schileyko 2011).

################ Remarks.

No material of this species was found, and only the type specimens were examined.

############### Neniauchenia tonkinensis

Taxon classificationAnimaliaStylommatophoraClausiliidae

Nordsieck, 2010

Neniauchenia tonkinensis Nordsieck, 2010: 46, 47, fig. 1. Type locality: Cuc Phuong N. P., Nho Quan District, Ninh Binh, Vietnam [error]. Schileyko 2011: 20.
Grandinenia tonkinensis : Páll-Gergely and Szekeres 2017: 515, fig. 6b. Type locality: Phong Nha–Ke Bang National Park of Quang Binh Province [correct type locality].

################ Material examined.

Holotype SMF 331370 (Fig. 28F).

################ Distribution.

Laos and Vietnam (Nordsieck 2010, Páll-Gergely and Szekeres 2017).

################ Remarks.

No material of this species was found, and only the type specimen was examined.

############## *Progarnieria* Nordsieck, 2012

############### Progarnieria huleschheliae

Taxon classificationAnimaliaStylommatophoraClausiliidae

(Grego & Szekeres, 2011)

Garnieria huleschheliae Grego & Szekeres, 2011: 14, 15, text figure and pl. 4, fig. 14. Type locality: northern region, Laos.
Progarnieria huleschheliae : Nordsieck 2012: 57, fig. 4, pl. 1, fig. 3.

################ Material examined.

**Figure 29.**
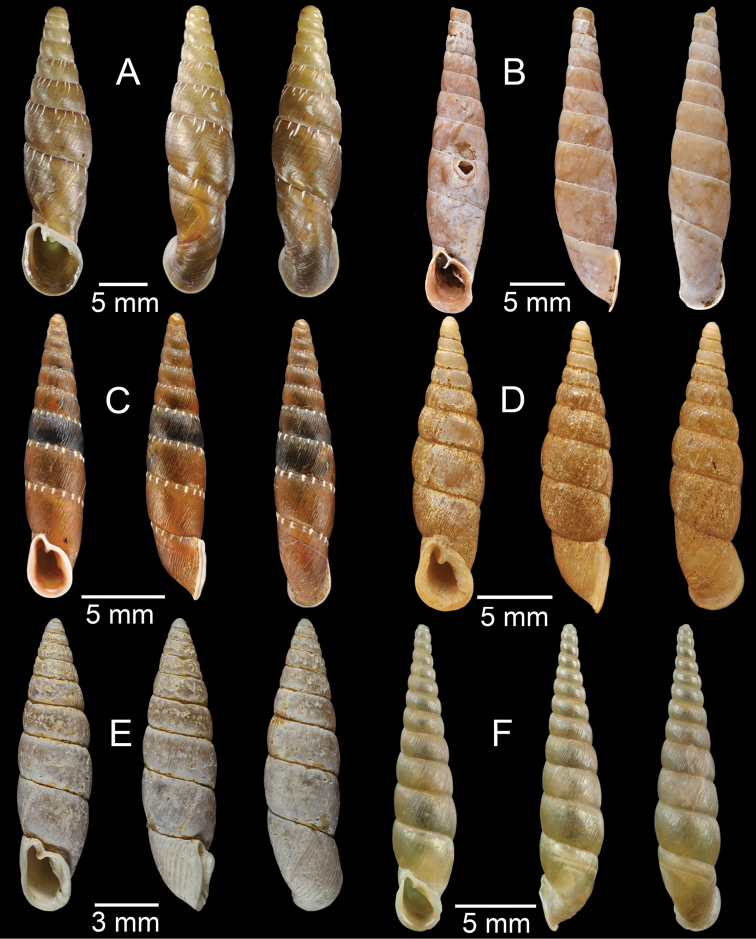
**A***Progarnieriahuleschheliae*, holotype SMF 334937 **B***Lindholmiellaahuiri*, paratype NHMUK 20100241 **C***Oospiraabstrusaginkae*, CUMZ collection **D***Oospiragregoi*, holotype NHMUK 20170227 **E***Oospiratetraptyx*, holotype MNHN-IM-2000-2219 **F***Phaedusamicropaviei*, holotype SMF 335898.

Holotype SMF 334937 (Fig. 29A).

################ Distribution.

Known only from the type locality in Laos (Grego and Szekeres 2011).

################ Remarks.

No material of this species was found, and only the type specimens were examined.

############# Subfamily Phaedusinae Wagner, 1922

############## *Lindholmiella* Ehrmann, 1927

############### Lindholmiella ahuiri

Taxon classificationAnimaliaStylommatophoraClausiliidae

Grego & Szekeres, 2011

Lindholmiella ahuiri Grego & Szekeres, 2011: 9, 10, text figure and pl. 2, fig. 7. Type locality: Vieng Xai, Houaphanh Province, Laos.

################ Material examined.

Paratypes NHMUK 20100241 (2 shells; Fig. 29B).

################ Distribution.

Known only from the type locality in Laos (Grego and Szekeres 2011).

################ Remarks.

No material of this species was found, and only the type specimens were examined.

############## *Oospira* Blanford, 1872

############### Oospira abstrusa ginkae

Taxon classificationAnimaliaStylommatophoraClausiliidae

Grego & Szekeres, 2014

Oospira abstrusa ginkae Grego & Szekeres in Grego et al. 2014: 752, 753, fig. 4. Type locality: entrance of the Pa Thom Cave, Tay Trang (Na-U), Dien Bien District, Dien Bien Province, Vietnam. Páll-Gergely and Szekeres 2017: 515, 516, fig. 7a–e.

################ Material examined.

Specimens from Phou Thaleang Bio-Diversity Conservation Area, Boun Neua District, Phongsaly Province (Fig. 29C).

################ Distribution.

Laos and Vietnam (Grego et al. 2014, Páll-Gergely and Szekeres 2017).

############### Oospira bolovenica

Taxon classificationAnimaliaStylommatophoraClausiliidae

(Möllendorff, 1898)

Clausilia (Hemiphaedusa) bolovenica Möllendorff, 1898: 76. Type locality: Boloven [Boloven Plateau, Paksong District, Champasak Province, Laos].
Clausilia (Hemiphaedusa) bolovenica Mut. gracilis Möllendorff, 1898: 76. Type locality: Boloven [Boloven Plateau, Paksong District, Champasak Province, Laos].
Hemiphaedusa (Hemiphaedusa) bolovenica : Zilch 1954a: 8, pl. 2, fig. 22. Schileyko 2011: 12.
Hemiphaedusa (Hemiphaedusa) bolovenicagracilis : Zilch 1954a: 8, pl. 2, fig. 23.
Oospira (Oospira) bolovenica : Nordsieck 2007: 23.

################ Material examined.

Lectotype of “*bolovenica* Möllendorff, 1898” SMF 62250 and paralectotypes SMF 62252 (6 shells), SMF 84922 (1 shell). Holotype of “*bolovenica* Mut. *gracilis* Möllendorff, 1898” SMF 62251.

################ Distribution.

Laos and possibly in Vietnam (Schileyko 2011).

################ Remarks.

No material of this species was found, and only the type specimens were examined. The original description did not include an illustration. Later, Zilch designated and illustrated the lectotype of the species (Zilch 1954a: pl. 2, fig. 22; see Fig. 17F).

############### Oospira gregoi

Taxon classificationAnimaliaStylommatophoraClausiliidae

Szekeres & Thach, 2017

Oospira (?) gregoi Szekeres & Thach in Thach, 2017: 29, 30, figs 350–354. Type locality: Attapeu Province, southeast of Laos, close to Vietnam border.

################ Material examined.

Holotype NHMUK 20170227 (Fig. 29D).

################ Distribution.

Known only from the type locality in Laos (Thach 2017).

################ Remarks.

No material of this species was found, and only the type specimens were examined.

############### Oospira tetraptyx

Taxon classificationAnimaliaStylommatophoraClausiliidae

Nordsieck, 2003

Oospira (Oospira) tetraptyx Nordsieck, 2003: 130, 131, pl. 2, fig. 10. Type locality: Xieng Khouang, Muong Phan [probably refers to Kham District, Xieng Khaung Province, Laos]. Nordsieck 2007: 24.

################ Material examined.

Holotype MNHN-IM-2000-2219 (Fig. 29E).

################ Distribution.

Known only from the type locality in Laos (Nordsieck 2003).

################ Remarks.

No material of this species was found, and only the type specimens were examined.

############## *Phaedusa* H. Adams & A. Adams, 1855

############### Phaedusa micropaviei

Taxon classificationAnimaliaStylommatophoraClausiliidae

Nordsieck, 2011

Phaedusa (Phaedusa) micropaviei Nordsieck, 2011: 159, fig. 11. Type locality: Moc Chau towards Son La road, Son La, Vietnam. Páll-Gergely and Szekeres 2017: 509, 520, fig. 1 (figure caption).

################ Material examined.

Holotype SMF 335898 (Fig. 29F) and paratypes SMF 335696 (1 shell), SMF 335899 (6 shells).

################ Distribution.

Laos and Vietnam (Nordsieck 2011, Páll-Gergely and Szekeres 2017).

################ Remarks.

No material of this species was found, and only the type specimens were examined.

############### Phaedusa paviei

Taxon classificationAnimaliaStylommatophoraClausiliidae

(Morlet, 1893)

Clausilia paviei Morlet, 1893[1892]: 319, 320, pl. 7, figs 1, 1a–c. Type locality: Muong-Laï, dans le Laos [probably refers to the area of Muong Cha District, Dien Bien Province, Vietnam]. Saurin 1953: 113.
Phaedusa paviei : Nordsieck 2011: 151. Schileyko 2011: 19, 20.

################ Material examined.

**Figure 30.**
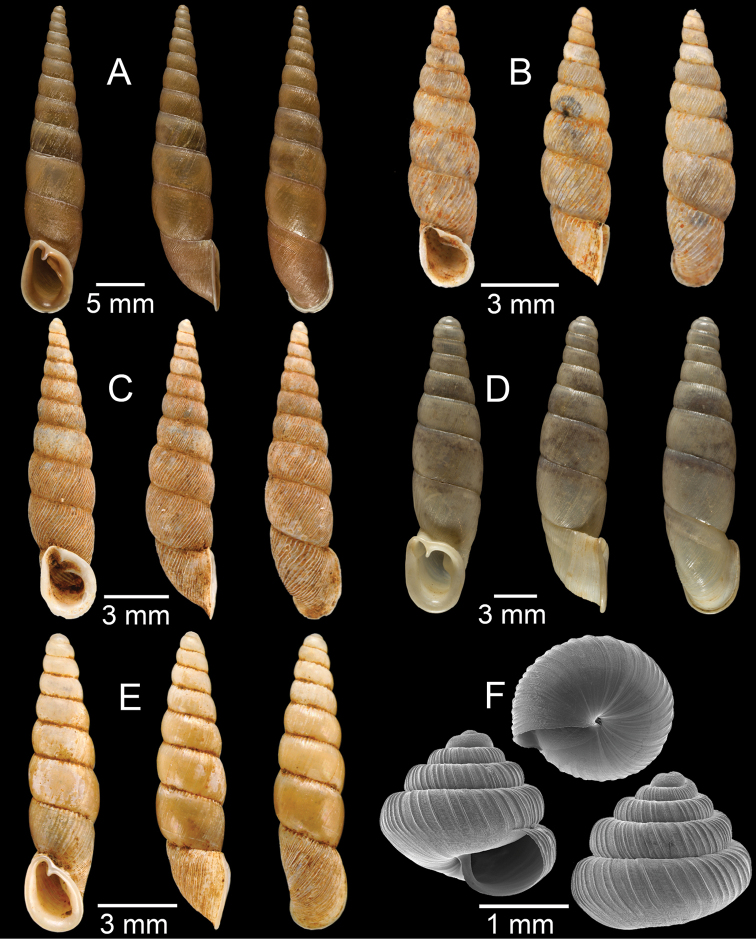
**A***Phaedusapaviei*, syntype MNHN-IM-2000-2655 **B, C***Phaedusapygmaea***B** paratype NHMUK 20100238 and **C** CUMZ collection **D, E***Synprosphymamoirati***D** syntype MNHN-IM-2000-2642 and **E** CUMZ collection **F***Kaliellaeurhabdota*, CUMZ collection.

Syntype MNHN-IM-2000-2655 from “Muong-Lai” (1 shell; Fig. 30A).

################ Distribution.

Laos and Vietnam (Saurin 1953, Nordsieck 2011).

################ Remarks.

No material of this species was found, and only the type specimen was examined.

############### Phaedusa pygmaea

Taxon classificationAnimaliaStylommatophoraClausiliidae

Grego & Szekeres, 2011

Phaedusa pygmaea Grego & Szekeres, 2011: 10, 11, text figure and pl. 2, fig. 8. Type locality: Hat Sao (Nong Khiaw), Luang Phrabang Province, Laos.

################ Material examined.

Paratype NHMUK 20100238 (1 shell; Fig. 30B). Specimens from Tam Phatok Cave, Ngoy District, Luang Phrabang Province (Fig. 30C).

################ Distribution.

Known only from the type locality in Laos (Grego and Szekeres 2011).

############## *Synprosphyma* Wagner, 1920

############### Synprosphyma moirati

Taxon classificationAnimaliaStylommatophoraClausiliidae

(Bavay & Dautzenberg, 1909)

Clausilia moirati Bavay & Dautzenberg, 1909a: 100–102, pl. 2, figs 10–12. Type locality: Pac Kha [Pa Kha Commune, Bac Ha District, Lao Cai Province, Vietnam], Muong Bo [probably refers to the Nam Sai Commune, Sa Pa District, Lao Cai Province, Vietnam] et Binh-Lu [Binh Lieu District, Quang Ninh Province, Vietnam].
Synprosphyma (Synprosphyma) moirati : Nordsieck 2007: 22.
Hemiphaedusa (Hemiphaedusa) moirati : Schileyko 2011: 12.

################ Material examined.

Syntype MNHN-IM-2000-2642 from “Pac-Kha, Muong-Bo et Binh-Lu” (1 shell; Fig. 30D). Specimens from Tam Phatok Cave, Ngoy District, Luang Phrabang Province (Fig. 30E).

################ Distribution.

Vietnam (Schileyko 2011).

########## Infraorder Arionoidei

########### Superfamily Arionoidea

############ Family Philomycidae Gray, 1847

############# *Meghimatium* van Hasselt, 1823

############## Meghimatium bilineatum

Taxon classificationAnimaliaStylommatophoraPhilomycidae

(Benson, 1842)

Incilaria bilineata Benson in Cantor, 1842: 486. Type locality: Chusan [China].
Meghimatium bilineata [sic]: Wiktor et al. 2000: 10–12, figs 11–13.

############### Material examined.

Specimens from Phou Thaleang Bio-Diversity Conservation Area, Boun Neua District, Phongsaly Province (Figs 19C, 55F).

############### Distribution.

Southern China (Wiktor et al. 2000).

############## Meghimatium pictum

Taxon classificationAnimaliaStylommatophoraPhilomycidae

(Stoliczka, 1873)

Philomycus pictus Stoliczka, 1873: 30, 31, pl. 3, figs 9–14. Type locality: Penang hill, Penang Island [Malaysia].
Meghimatium cf. pictum : Wiktor et al. 2000: 12, 13, figs 14–17.

############### Material examined.

Specimens from Phou Thaleang Bio-Diversity Conservation Area, Boun Neua District, Phongsaly Province (Figs 19D, 55G).

############### Distribution.

China and Malaysia (Stoliczka 1873, Wiktor et al. 2000).

########## Infraorder Limacoidei [= “Limacoid Clade”]

########### Superfamily Trochomorphoidea

############ Family Chronidae Thiele, 1931

############# *Kaliella* Blanford, 1863

############## Kaliella eurhabdota

Taxon classificationAnimaliaStylommatophoraChronidae

Saurin, 1953

Kaliella eurhabdota Saurin, 1953: 118, pl. 4, fig. 9a–d. Type locality: environs du village méo de Pah Hia, à 100 kilomètres au Sud de Xieng-Khouang, chef-lieu de la province du Tran Ninh, Laos [probably refers to Ban Namthong, Longchaeng District, Xaisomboun Province, Laos].

############### Material examined.

Specimens from km 159-177 road from Hoauisai, Ban Nam Thoung Village, Hoauixai District, Bokeo Province (Fig. 30F).

############### Distribution.

Known only from the type locality in Laos (Saurin 1953).

############### Remarks.

For the current interpretation of Pa Hia, see Páll-Gergely et al. (2016: 13).

############## Kaliella micracyna

Taxon classificationAnimaliaStylommatophoraChronidae

Saurin, 1953

Kaliella micracyna Saurin, 1953: 117, pl. 4, fig. 10a, b. Type locality: environs du village méo de Pah Hia, à 100 kilomètres au Sud de Xieng-Khouang, chef-lieu de la province du Tran Ninh, Laos [probably refers to Ban Namthong, Longchaeng District, Xaisomboun Province, Laos].

############### Distribution.

Known only from the type locality in Laos (Saurin 1953).

############### Remarks.

No material of this species was found, and the type specimen could not be traced. This species was figured in Saurin (1953: pl. 4, fig. 10a, see Fig. 17G). For the current interpretation of Pa Hia, see Páll-Gergely et al. (2016: 13).

############## Kaliella muongomensis

Taxon classificationAnimaliaStylommatophoraChronidae

Saurin, 1953

Kaliella muongomensis Saurin, 1953: 117, pl. 4, figs 6a, b, 7. Type locality: Muong Om, voisine de Pah Hia, à 100 kilomètres au Sud de Xieng-Khouang, chef-lieu de la province du Tran Ninh, Laos [probably refers to Ban Namthong, Longchaeng District, Xaisomboun Province, Laos].

############### Material examined.

**Figure 31.**
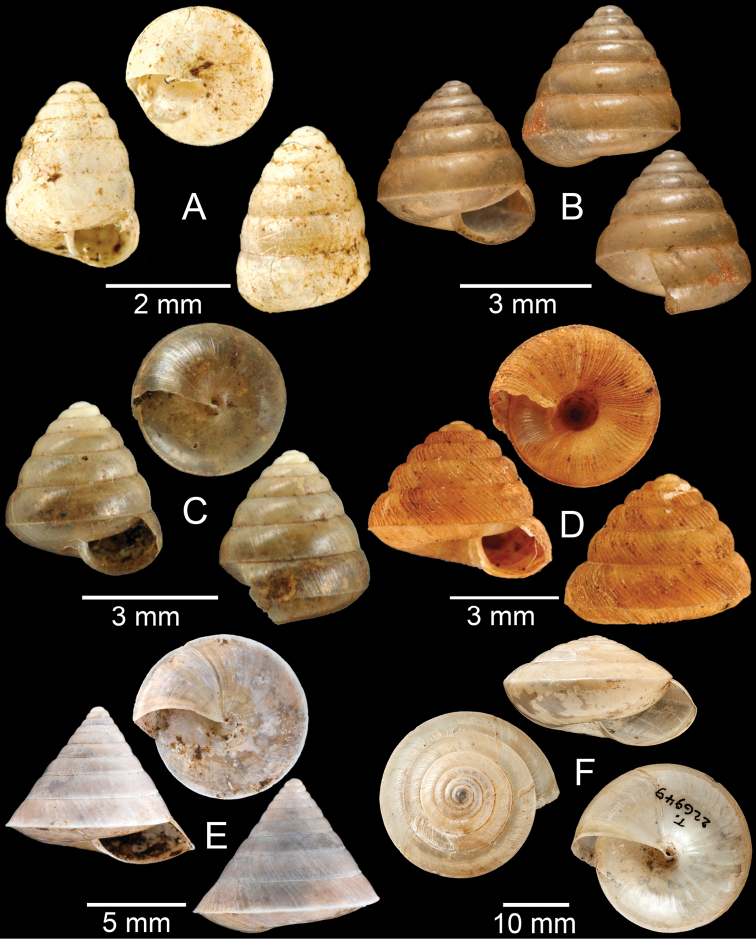
**A***Kaliellamuongomensis*, CUMZ collection **B, C***Kaliellaordinaria***B** syntype MNHN-IM-2000-9660 and **C** CUMZ collection **D***Kaliellaornatissima*, CUMZ collection **E***Kaliellatongkingensis*, CUMZ collection **F***Quantulatenera*, holotype SMF 226949.

Specimens from Par-Houak limestone, Ban Vieng Swarng village, Vieng Phouka District, Luang Namtha Province (Fig. 31A).

############### Distribution.

Known only from the type locality in Laos (Saurin 1953).

############### Remarks.

For the current interpretation of Pa Hia, see Páll-Gergely et al. (2016: 13).

############## Kaliella ordinaria

Taxon classificationAnimaliaStylommatophoraChronidae

Ancey, 1904

Kaliella ordinaria Ancey in Bavay and Dautzenberg 1904[1903]: 210, 211, pl. 8, figs 18, 19. Type locality: Van Bu, Tonkin Occidental [Van Ban District, Lao Cai Province, Vietnam]. Saurin 1953: 113. Wood and Gallichan 2008: 72. Schileyko 2011: 28.

############### Material examined.

Syntype MNHN-IM-2000-9660 from “Van Bu, Tonkin Occidental; Haut Tonkin” (1 shell; Fig. 31B). Specimens from Ban Nong Kham village, Kasy District, Vientiane Province (Fig. 31C).

############### Distribution.

Laos and Vietnam (Saurin 1953, Schileyko 2011).

############### Remarks.

For the correct authorship of the name, see Wood and Gallichan (2008: 72).

############## Kaliella ornatissima

Taxon classificationAnimaliaStylommatophoraChronidae

Bavay & Dautzenberg, 1912

Kaliella ornatissima Bavay & Dautzenberg, 1912: 14, 15, pl. 2, figs 13–16. Type locality: Trinh-Tuong [Trinh Tuong Commune, Bat Xat District, Lao Cai Province, Vietnam] et Binh-Lu, Tonkin [Binh Lieu District, Quang Ninh Province, Vietnam]. Saurin 1953: 113. Schileylo 2011: 28.

############### Material examined.

Specimens from Par-Houak limestone, Ban Vieng Swarng village, Vieng Phouka District, Luang Namtha Province (Fig. 31D).

############### Distribution.

Laos and Vietnam (Saurin 1953, Schileyko 2011).

############## Kaliella tongkingensis

Taxon classificationAnimaliaStylommatophoraChronidae

Möllendorff, 1901

Kaliella tongkingensis Möllendorff, 1901a: 70. Type locality: Than-moi, Mansongebirge [Mou Son Mountain, Lang Son Province, Vietnam]. Bavay and Dautzenberg 1904[1903]: 209, pl. 8, figs 14–17. Schileyko 2011: 29.
Nanina (Kaliella) tongkingensis : Kobelt 1905: 1194, 1195, pl. 296, figs 13, 14.

############### Material examined.

Specimens from limestone outcrops near Ngoi Town, Ngoy District, Luang Phrabang Province (Fig. 31E).

############### Distribution.

Vietnam (Schileyko 2011).

############ Family Dyakiidae Gude & Woodward, 1921

############# *Quantula* Baker, 1941

############## Quantula tenera

Taxon classificationAnimaliaStylommatophoraDyakiidae

(Möllendorff, 1901)

Xestina tenera Möllendorff, 1901b: 46. Type locality: Ballach, Süd-Annam [Southern Vietnam]. Dautzenberg and Fischer 1906[1905]: 350.
Nanina (Xestina) tenera : Kobelt 1902b: 1077, pl. 273, figs 10–12. Fischer and Dautzenberg 1904: 394.
Quantula tenera tenera : Schileyko 2011: 37.

############### Material examined.

**Figure 32.**
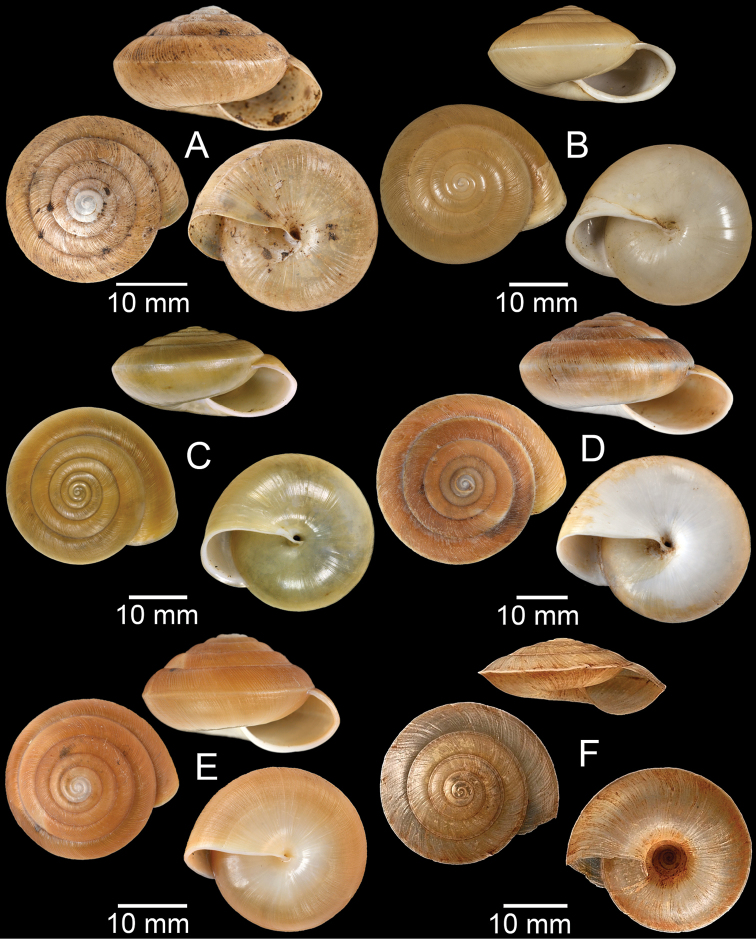
**A***Quantulatenera*, CUMZ collection **B–D***Quantulaweinkauffiana***B** syntype MNHN-IM-2000-27780 and **C, D** CUMZ collection **E***Quantula* sp., CUMZ collection **F***Trochomorphabenigna*, syntype NHMUK ex. Cuming collection.

Holotype SMF 226949 (Fig. 31F). Specimen from Ban Pak-kard Village, Pek District, Xieng Khaung Province (Fig. 32A).

############### Distribution.

Vietnam (Fischer and Dautzenberg 1904, Schileyko 2011).

############## Quantula weinkauffiana

Taxon classificationAnimaliaStylommatophoraDyakiidae

(Crosse & Fischer, 1863)

Helix weinkauffiana Crosse & Fischer, 1863b: 350, 351. Type locality: Cochinchine [Southern Vietnam]. Crosse and Fischer 1864: 326, pl. 12, fig. 7.
Nanina (Xestina) weinkauffiana : Kobelt 1900: 984, 985, pl. 255, figs 6–8.
Ariophanta (Cryptozona) weinkauffiana : Schileyko 2011: 29, 30.

############### Material examined.

Syntypes MNHN-IM-2000-27780 from “Cochinchine” (3 shells; Fig. 32B). Specimens from Ban Sisawarng village, Xayphouthong District, Savannakhet Province (Figs 32C, 55H). Specimens from Ban Phone Pai village, Bachiang District, Champasak Province (Fig. 32D).

############### Distribution.

Cambodia, Laos, Thailand and Vietnam (Schileyko 2011).

############## Quantula

Taxon classificationAnimaliaStylommatophoraDyakiidae

sp.

############### Material examined.

Specimens from limestone outcrop at Tam Nang Rod Cave, Na-dan village, Yommalath District, Khammouan Province, Laos (Fig. 32E).

############### Remarks.

These specimens differ from *Quantulaweinkauffiana* in having a smaller shell, upper shell surface with prominent nodules arranged on transverse ridges, and these ridges diminish below the periphery. In contrast, *Q.weinkauffiana* has a larger shell, an upper shell surface with smooth transverse ridges that terminate at the peripheral keel, and a smooth surface below the periphery.

############ Family Trochomorphidae Möllendorff, 1890

############# *Trochomorpha* Albers, 1850

############## Trochomorpha benigna

Taxon classificationAnimaliaStylommatophoraTrochomorphidae

(Pfeiffer, 1863)

Helix benigna Pfeiffer, 1863a[1862]: 269, pl. 36, figs 11, 12. Type locality: Lao Mountains, Camboja [Cambodia or Laos].
Trochomorpha benigna : Fischer and Dautzenberg 1904: 398.

############### Material examined.

Syntypes NHMUK ex. Cuming collection from “Lao Mountains, Camboja” (3 shells; Fig. 32F).

############### Distribution.

Laos (Fischer and Dautzenberg 1904).

############### Remarks.

No material of this species was found, and only the type specimens were examined.

############## Trochomorpha paviei

Taxon classificationAnimaliaStylommatophoraTrochomorphidae

(Morlet, 1885)

Helix paviei Morlet, 1885[1884]: 386, 387, pl. 11, figs 1, 1a. Type locality: dans les forêts, entre Kampot et Phnom-Penh, particulièrement près des rapides de Kamchay (rivière de Kampot), sur les bois pourris et les petite plantes [In forests, between Kampot and Phnom Penh, especially near the rapids Kamchay (Kampot River), on rotten wood and small plants].
Sivella paviei : Schileyko 2011: 35.

############### Material examined.

**Figure 33.**
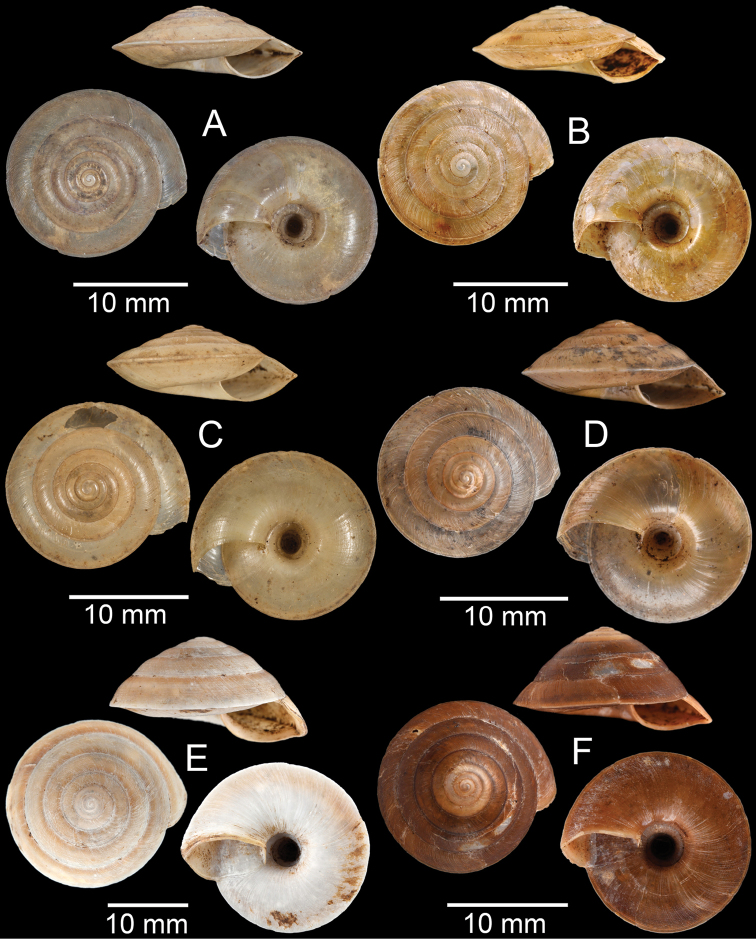
**A, B***Trochomorphapaviei***A** syntype MNHN-IM-2000-27885 and **B** CUMZ collection **C, D***Trochomorphasaigonensis***C** syntype MNHN-IM-2000-27875 and **D** CUMZ collection **E***Trochomorpha* (?) sp. 1, CUMZ collection **F***Trochomorpha* (?) sp. 2, CUMZ collection.

Syntype MNHN-IM-2000-27885 from “Kampoi et Phnom-Penh” (1 shell; Fig. 33A). Specimens from Phou Thaleang Bio-Diversity Conservation Area, Boun Neua District, Phongsaly Province (Figs 33B, 56A).

############### Distribution.

Cambodia, Laos and Vietnam (Schileyko 2011).

############## Trochomorpha saigonensis

Taxon classificationAnimaliaStylommatophoraTrochomorphidae

(Crosse, 1867)

Helix saigonensis Crosse, 1867: 208, 209, pl. 6, fig. 3. Type locality: in provincia Saigonensi et in insula Poulo-Condor dicta, Cochinchinae gallicae [Ho Chi Minh City and Con Dao Islands, Ba Ria–Vung Tau Province, Vietnam].
Geotrochus saigonensis : Schileyko 2011: 36.

############### Material examined.

Syntype MNHN-IM-2000-27875 “Poulo Condor” (1 shell; Fig. 33C). Specimens from limestone hills at Ban Oudom village, Pakbeg Ditrict, Oudomxay Province (Fig. 33D).

############### Distribution.

Cambodia and Vietnam (Schileyko 2011).

############## Trochomorpha

Taxon classificationAnimaliaStylommatophoraTrochomorphidae

(?) sp. 1

############### Material examined.

Specimens from Tam Nang Rod Cave, Na-dan village, Yommalath District, Khammouan Province (Fig. 33E).

############### Remarks.

These specimens differ from all other known species in Indochina by having a trochiform with a dome-shaped shell with the upper periphery dome-shaped and the lower periphery flattened; the shell is thickened and relatively large; last whorl with a sharp peripheral keel; upper periphery with irregular growth lines and brownish subsutural band; aperture angulated, lip simple and slightly thickened; umbilicus widely open and deep.

The generic assignment of this species in *Trochomorpha* s.l. is still provisional, and additional anatomical studies are necessary to confirm the systematic position of this species.

############## Trochomorpha

Taxon classificationAnimaliaStylommatophoraTrochomorphidae

(?) sp. 2

############### Material examined.

Specimens from Tam Xang Cave, Thakhek District, Khammouan Province (Fig. 33F).

############### Remarks.

These specimens obviously differ from *Trochomorphapaviei* by having a trochiform shell with the upper periphery dome-shaped and the lower periphery flattened; shell thickened, with monochrome dark brown colour; shell surface with thin transverse ridges; apertural lip slightly thickened; umbilicus open and deep. In contrast, *Trochomorphapaviei* has a trochiform shell with the upper periphery depressed, dome-shaped and the lower periphery convex; shell thin, translucent with monochrome brownish colour; apertural lip simple; umbilicus widely open and deep.

The generic assignment of this species in *Trochomorpha* s.l. is still provisional, and additional anatomical studies are necessary to confirm the systematic position of this species.

############## Trochomorpha

Taxon classificationAnimaliaStylommatophoraTrochomorphidae

sp. 3.

############### Material examined.

**Figure 34.**
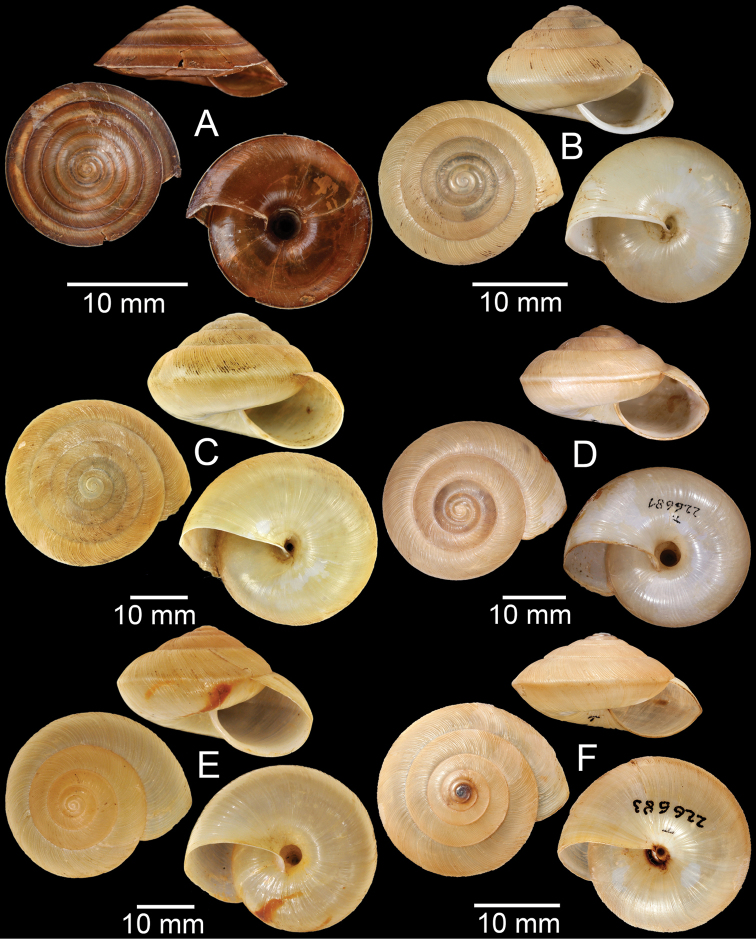
**A***Trochomorpha* sp. 3, CUMZ collection **B***Ariophantacrossei*, syntype MNHN-IM-2000-1869 **C***Ariophantadanae*, syntype NHMUK 20090243 **D, E***Ariophantalaotica***D** syntype SMF 226681 and **E** specimen NHMUK 1902.7.19.38 **F***Ariophantaprionotropis*, syntype SMF 226683. Photo: B. Páll-Gergely (**D**).

Specimens from Par-Houak limestone, Ban Vieng Swang village, Vieng Phoukha District, Luang Namtha Province (Fig. 34A).

############### Remarks.

These specimens are similar to *Trochomorphabicolor* Martens, 1864, but the distinct characters are an elevated conical shell with flattening below the periphery, and a narrower and deep umbilicus.

The generic assignment of this species in *Trochomorpha* s.l. is still provisional, and additional anatomical studies are necessary to confirm the systematic position of this species.

########### Superfamily Helicarionoidea

############ Family Ariophantidae Godwin-Austen, 1888

############# *Ariophanta* Des Moulins, 1829

############## Ariophanta crossei

Taxon classificationAnimaliaStylommatophoraAriophantidae

(Pfeiffer, 1862)

Helix crossei Pfeiffer, 1862: 39, pl. 5, figs 2, 3. Type locality: Siam [Thailand].
Nanina (Xestina) crossei : Kobelt 1900: 983, 984, pl. 255, fig. 5, pl. 256, figs 3, 4.
Nanina (Hemiplecta) crossei : Fischer and Dautzenberg 1904: 393.
Ariophanta (Cryptozona) crossei : Schileyko 2011: 29.

############### Material examined.

Syntype MNHN-IM-2000-1869 from “Siam” (1 shell; Fig. 34B).

############### Distribution.

Cambodia, Laos, Thailand and Vietnam (Schileyko 2011).

############### Remarks.

No material of this species was found, and only the type specimen was examined.

############## Ariophanta danae

Taxon classificationAnimaliaStylommatophoraAriophantidae

(Pfeiffer, 1863)

Helix danae Pfeiffer, 1863a[1862]: 268. Type locality: Lao Mountains, Camboja [Cambodia or Laos].
Nanina (Xestina) danae : Kobelt 1902b: 1076, pl. 273, figs 4–6.
Ariophanta (Cryptozona) danae : Schileyko 2011: 29.

############### Material examined.

Syntype NHMUK 20090243 from “Lao Mountains, Camboja” (1 shell; Fig. 34C).

############### Distribution.

Laos and possibly Vietnam (Schileyko 2011).

############### Remarks.

No material of this species was found, and only the type specimen was examined.

############## Ariophanta laotica

Taxon classificationAnimaliaStylommatophoraAriophantidae

(Möllendorff, 1899)

Bensonia (Oxytes) laotica Möllendorff, 1899: 165. Type locality: Oberer Mekong im Lande der Laos [Upper Mekong in Laos].
Nanina (Oxytes) laotica : Kobelt 1902b: 1091. Kobelt 1904: pl. 276, figs 4, 5.

############### Material examined.

Syntype SMF 226681 from “Laos” (1 shell; Fig. 34D). Specimen NHMUK 1902.7.19.38 from “Laos, Shan State” (1 shell; Fig. 34E).

############### Distribution.

Known only form the type locality in Laos (Kobelt 1902b).

############### Remarks.

No material of this species was found, and only the type specimen was examined.

############## Ariophanta prionotropis

Taxon classificationAnimaliaStylommatophoraAriophantidae

(Möllendorff, 1898)

Bensonia prionotropis Möllendorff, 1898: 69, 70. Type locality: Boloven [Boloven Plateau, Paksong District, Champasak Province, Laos].
Ariophanta (?) (Cryptozona) prionotropis: Schileyko 2011: 29.

############### Material examined.

Syntype SMF 226683 from “Boloven” (1 shell; Fig. 34F).

############### Distribution.

Laos and probably in Vietnam (Schileyko 2011).

############### Remarks.

No material of this species was found, and only the type specimen was examined.

############# *Cryptosemelus* Collinge, 1902

############## Cryptosemelus

Taxon classificationAnimaliaStylommatophoraAriophantidae

sp.

############### Material examined.

Specimens from plantation near Ngoy Town, Ngoy District, Luang Phrabang Province (Figs 19E, 57B).

############### Remarks.

The genus was first described from Peninsula Malaysia. These semi-slugs were founded on pomelo (*Citrusmaxima* Merr) leaves. This is probably the second species of the genus. However, the genitalia characters of the type species are still unknown (Schileyko 2003a).

############# *Cryptozona* Mörch, 1872

############## Cryptozona siamensis

Taxon classificationAnimaliaStylommatophoraAriophantidae

(Pfeiffer, 1856)

Helix siamensis Pfeiffer, 1856a: 32. Type locality: Siam [Thailand]. Pfeiffer 1856b: 76, 77, pl. 21, figs 7–9.
Nanina (Hemiplecta) siamensis : Kobelt 1905: 1136, pl. 285, figs 10, 11.
Hemiplecta (Hemiplecta) siamensis : Solem 1966: 27.
Cryptozona siamensis : Hemmen and Hemmen 2001: 43.

############### Material examined.

**Figure 35.**
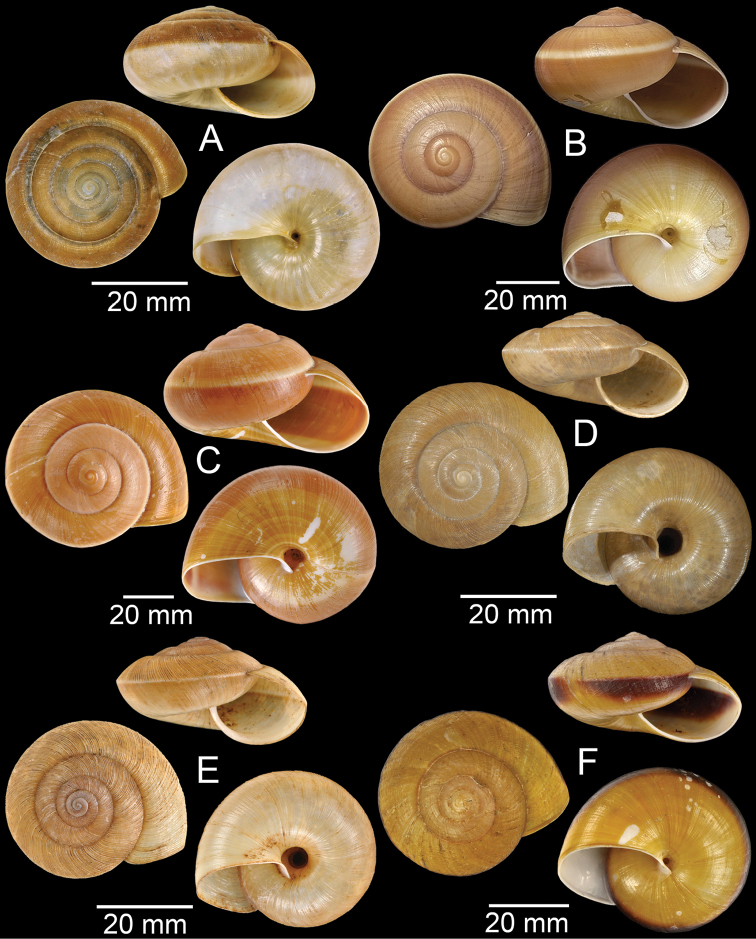
**A***Cryptozonasiamensis*, CUMZ collection **B, C***Hemiplectadistincta***B** possible syntype NHMUK ex. Cuming collection and **C** CUMZ collection **D, E***Hemiplectaesculenta***D** holotype RMNH 99424 and **E** CUMZ collection **F***Hemiplectafunerea*, syntype NHMUK 1896.1.25.4. Photo: W.J.M. Maassen (**B**).

Specimens from Tad Pha Soam waterfall, Paksong District, Champasak Province (Figs 35A, 56B).

############### Distribution.

Cambodia, Laos and Thailand (Solem 1966).

############# *Hemiplecta* Albers, 1850

############## Hemiplecta distincta

Taxon classificationAnimaliaStylommatophoraAriophantidae

(Pfeiffer, 1850)

Helix distincta Pfeiffer, 1850: 69, 70. Type locality: insulis Moluccis [Molucca Islands]. Pfeiffer 1853b: 346, pl. 134, figs 1, 2.
Nanina distincta : Martens 1867: 69, 70, pl. 6, fig. 8.
Rhysota distincta : Saurin 1953: 113.
Hemiplecta (Koratia) distincta : Solem 1966.
Koratia distincta : Schileyko 2011: 30.

############### Material examined.

Possible syntypes NHMUK ex. Cuming collection from “Siam and Camboja” (3 shells; Fig. 35B). Specimens from Ban Xaynapho village, Pathoumphone District, Champasak Province (Figs 35C, 56C).

############### Distribution.

Cambodia, Laos, Thailand and Vietnam (Saurin 1953, Solem 1966, Schileyko 2011).

############## Hemiplecta esculenta

Taxon classificationAnimaliaStylommatophoraAriophantidae

Maassen, 2006

Hemiplecta esculenta Maassen, 2006a: 17, 18, figs 10–12. Type locality: limestone area near village Hang, NW-point Pu Luong National Park, Thanh Hoa Province, Vietnam.

############### Material examined.

Holotype RMNH 99424 (Fig. 35D). Specimens from Ban Nong Tang village, Phookood District, Xieng Khaung Province (Fig. 35E).

############### Distribution.

Vietnam (Maassen 2006a)

############## Hemiplecta funerea

Taxon classificationAnimaliaStylommatophoraAriophantidae

(Smith, 1896)

Nanina distincta var. funerea Smith, 1896: 128. Type locality: Vanbu, Tonkin [Van Ban District, Lao Cai Province, Vietnam]. Schileyko 2011: 30.
Nanina (Rhysota) distinctavar.funerea : Fischer and Dautzenberg 1904: 393.

############### Material examined.

**Figure 36.**
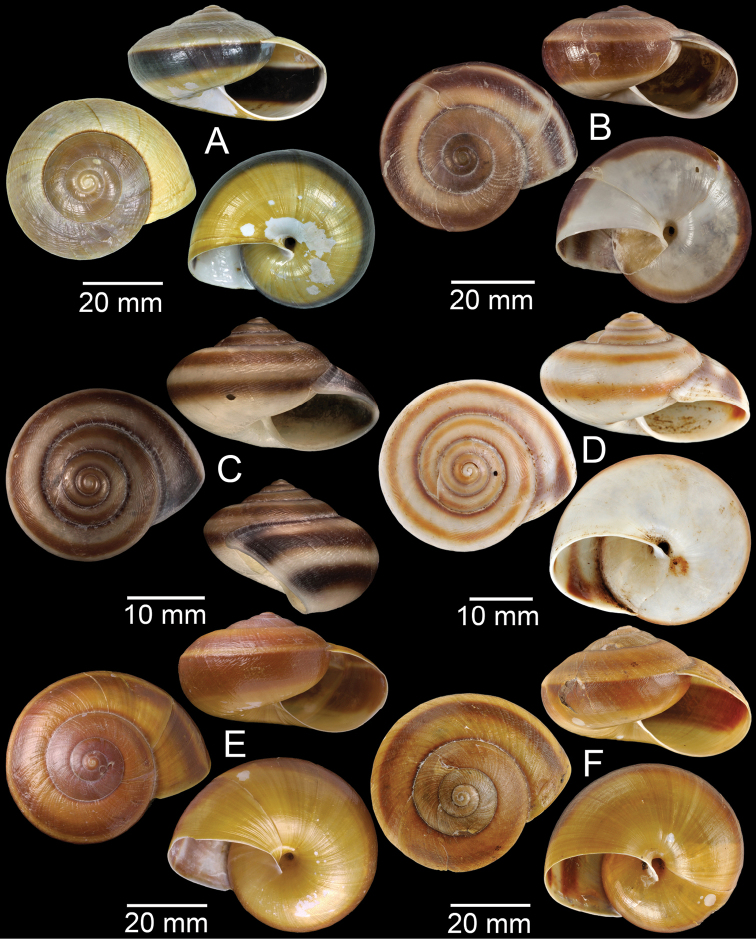
**A***Hemiplectafunerea*, CUMZ collection **B***Hemiplectahuberi*, holotype MNHN-IM-2000-33196 **C, D***Hemiplectalanxangnica* Inkhavilay and Panha nom. nov. **C** paratype MNHN-IM-2000-33215 and **D** CUMZ collection **E, F***Hemiplectapluto***E** syntype NHMUK ex Cuming collection and **F** CUMZ collection.

Syntype NHMUK 1896.1.25.4 of “var. *funerea*” from “Vanbu, Tonkin” (1 shell; Fig. 35F). Specimens from Nam Noua Bridge, Viengxay District, Houaphanh Province (Fig. 36A).

############### Distribution.

Vietnam (Schileyko 2011).

############## Hemiplecta huberi

Taxon classificationAnimaliaStylommatophoraAriophantidae

Thach, 2017

Hemiplecta huberi Thach, 2017: 33, figs 389–391. Type locality: Thakhek, Khammouane Province, Central Laos.

############### Material examined.

Holotype MNHN-IM-2000-33196 (Fig. 36B).

############### Distribution.

Known only from the type locality in Laos (Thach 2017).

############### Remarks.

No material of this species was found, and only the type specimens were examined.

############## Hemiplecta lanxangnica

Taxon classificationAnimaliaStylommatophoraAriophantidae

Inkhavilay & Panha nom. nov.

Helminthoglypta huberi Thach, 2017: 54, figs 747–749 [non Hemiplectahuberi Thach, 2017: 33, figs 389–391]. Type locality: Thakhek, Khammouane Province, Central Laos.

############### Etymology.

The species name “*lanxangnica*” is derived from “Lan Xang”, the name of the historical empire during 13^th^ to 18^th^ centuries, which represents the current Laotian area.

############### Material examined.

Holotype RMNH 5006710, paratype MNHN-IM-2000-33215 (1 shell; Fig. 36C). Specimens from Tam Xang Cave, Thakhek District, Khammouan Province (Fig. 36D).

############### Distribution.

Known only from the type locality in Laos (Thach 2017).

############### Remarks.

The genus *Helminthoglypta* Ancey, 1887 is mainly distributed in the California and northwest of Mexico (Schileyko 2004: 1722). In addition, the Xanthonychidae, to which this genus belongs, is mainly distributed in the New World: Central America, North America and north of South America (Schileyko 2004) and has never been recorded in the Oriental region. Placing this species in the *Helminthoglypta* seemed inappropriate. The medium size (width 27–34 mm and height 18–23 mm), rimate umbilicus, thin wrinkle shell surface and simple apertural lip make this species more closely resemble the genus *Hemiplecta*. However, examination of the genitalia anatomy is required to confirm their systematic position.

By relocating *Helminthoglyptahuberi* Thach, 2017 to the genus *Hemiplecta*, it becomes a secondary homonym of *Hemiplectahuberi* Thach, 2017. According to the ICZN guideline (ICZN 1999: Arts 24.2.2, 57.3.1 and 60.3), the species name of a junior homonym has to be replaced, and so we propose *Hemiplectalanxangnica* Inkhavilay & Panha nomen novum as the new replacement name.

############## Hemiplecta pluto

Taxon classificationAnimaliaStylommatophoraAriophantidae

(Pfeiffer, 1863)

Helix pluto Pfeiffer, 1863a[1862]: 268, 269. Type locality: Lao Mountains, Camboja [Cambodia or Laos]. Pfeiffer 1863b: 210, pl. 55, figs 8, 9.
Nanina (Hemiplecta) pluto : Kobelt 1900: 987, pl. 256, figs 1, 2.
Hemiplecta pluto : Schileyko 2011: 30.

############### Material examined.

Syntypes NHMUK ex Cuming collection (2 shells; Fig. 36E). Specimens from Ban Phone Can village, Yommalath District, Khammouan Province (Figs 36F, 56D).

############### Distribution.

Cambodia, Laos, Thailand and Vietnam (Kobelt 1900, Schileyko 2011).

############# *Macrochlamys* Benson, 1832

############## Macrochlamys callojuncta

Taxon classificationAnimaliaStylommatophoraAriophantidae

Ancey, 1898

Macrochlamys callojuncta Ancey, 1898: 129. Type locality: Luang-prabang, Laos [Luang Phrabang Province, Laos]. Wood and Gallichan 2008: 32.

############### Material examined.

**Figure 37.**
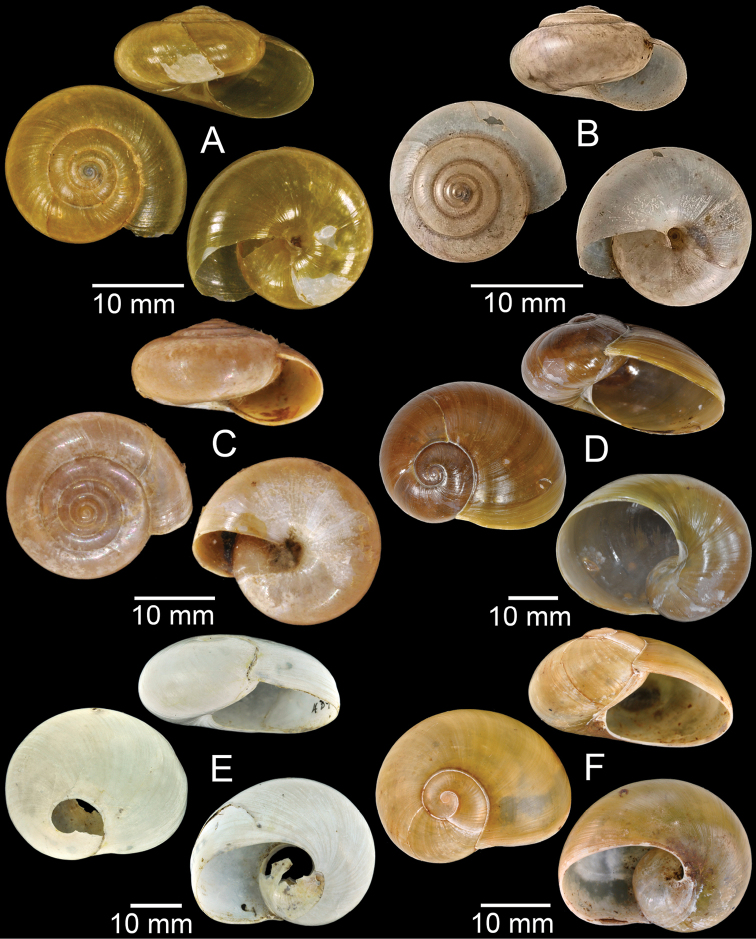
**A***Macrochlamyscallojuncta*, CUMZ collection **B***Macrochlamys* (?) *mitis*, syntype NHMUK ex. Cuming collection **C***Macrochlamys* (?) *tecta*, syntype NHMUK 1854.7.24.351 **D***Megausteniamalefica*, CUMZ collection **E, F***Megausteniasiamensis***E** syntype AMNH 43912 and **F** CUMZ collection.

Specimens from Ban Na Bia (Ban 019) village, Ngoy District, Luang Phrabang Province (Figs 37A, 56E).

############### Distribution.

Known only from the type locality in Laos (Ancey 1898).

############## Macrochlamys (?) mitis

Taxon classificationAnimaliaStylommatophoraAriophantidae

(Pfeiffer, 1863)

Helix mitis Pfeiffer, 1863a[1862]: 268. Type locality: Lao Mountains, Camboja [Cambodia or Laos]. Pfeiffer 1868a: 141.
Ariophanta (Kaliella) mitis : Fischer 1891: 21.
Hyalinia mitis : Fischer and Dautzenberg 1904: 396.

############### Material examined.

Syntypes NHMUK ex. Cuming collection from “Lao Mountains, Camboja” (2 shells; Fig. 37B).

############### Distribution.

Cambodia and Laos (Fischer 1891).

############### Remarks.

No material of this species was found, and only the type specimens were examined. The generic placement of this species is provisional. We placed this species into *Macrochlamys* s.l. because it has a depressed conic shell, thin and translucent shell, smooth and shining shell surface, with 4 to 7 whorls and the last whorl rounded, and a simple apertural lip. However, this species differs slightly from *Macrochlamys* s.l. in having varices and a widely opened umbilicus (see Godwin-Austen (1883: 76–84) and Blanford and Godwin-Austen (1908: 77–79) for further comparison).

############## Macrochlamys (?) tecta

Taxon classificationAnimaliaStylommatophoraAriophantidae

(Souleyet, 1852)

Vitrina tecta Souleyet in Eydoux & Souleyet, 1852: 499, 500, pl. 28, figs 15–17. Type locality: environs de Touranne, en Cochinchine [Da Nang Province, Vietnam].
Megaustenia (?) tecta: Schileyko 2011: 32.

############### Material examined.

Syntypes NHMUK 1854.7.24.351 (2 shells; Fig. 37C).

############### Distribution.

Laos and Vietnam (Schileyko 2011).

############### Remarks.

No material of this species was found, and only the type specimens were examined. Recently, Schileyko (2011) placed this species within the semi-slug genus *Megaustenia*. However, the syntypes have 5 or 6 slowly increasing whorls and a narrowly opened umbilicus, so it is more appropriate to relocate it to *Macrochlamys* s.l. This generic placement is provisional and waiting for future genitalia information. Note that Souleyet (1852: pl. 28, fig. 15) illustrated a live specimen with large mantle lobes, and without a caudal foss and caudal horn on the posterior end of the body, which is distinct from the typical Indian *Macrochlamys* s.l. (see Godwin-Austen (1883: 76–84) and Blanford and Godwin-Austen (1908: 77–79) for further comparison).

############# *Megaustenia* Cockerell, 1912

############## Megaustenia malefica

Taxon classificationAnimaliaStylommatophoraAriophantidae

(Mabille, 1887)

Helicarion maleficus Mabille, 1887a: 2. Type locality: Tonkin [Northern Vietnam]. Mabille 1887b: 74, 75, pl. 1, figs 10–12. Fischer and Dautzenberg 1904: 392. Schileyko 2011: 32.
Cryptosoma maleficum : Kobelt 1905: 1196, 1197, pl. 297, figs 4–6.

############### Material examined.

Specimens from limestone hill at Ban Nathan village, Viengxay District, Houaphanh Province (Figs 37D, 56F).

############### Distribution.

Laos and Vietnam (Fischer and Dautzenberg 1904, Schileyko 2011).

############## Megaustenia siamensis

Taxon classificationAnimaliaStylommatophoraAriophantidae

(Haines, 1855)

Vitrina siamensis Haines, 1855: 158. Type locality: Siam [Thailand].
Cryptosoma siamensis : Kobelt 1905: 1197, 1198, pl. 297, figs 7–9.
Megaustenia siamensis : Solem 1966: 78–84, figs 17–19.
Megaustenia siamense [sic]: Schileyko 2011: 32.

############### Material examined.

Syntype AMNH 43912 from “Siam” (1 shell; Fig. 37E). Specimens from Ban Namone village, Xayaboury District, Xayaboury Province (Figs 37F, 56G).

############### Distribution.

Cambodia, Myanmar, Thailand and Vietnam (Solem 1966, Schileyko 2011).

############# *Microcystina* Mörch, 1872

############## Microcystina annamitica

Taxon classificationAnimaliaStylommatophoraAriophantidae

(Möllendorff, 1898)

Lamprocystis annamitica Möllendorff, 1898: 68. Type locality: Boloven [Boloven Plateau, Paksong District, Champasak Province, Laos].
Microcystina (?) annamitica: Schileyko 2011: 33.

############### Distribution.

Laos and Vietnam (Schileyko 2011).

############### Remarks.

No material of this species was found.

############## Microcystina messageri

Taxon classificationAnimaliaStylommatophoraAriophantidae

Ancey, 1904

Microcystina messageri Ancey in Bavay & Dautzenberg, 1904[1903]: 207, 208, pl. 8, figs 8–10. Type locality: Bac-Kan [Bac Kan Province, Vietnam]. Saurin 1953: 113. Wood and Gallichan 2008: 66. Schileyko 2011: 33.

############### Material examined.

**Figure 38.**
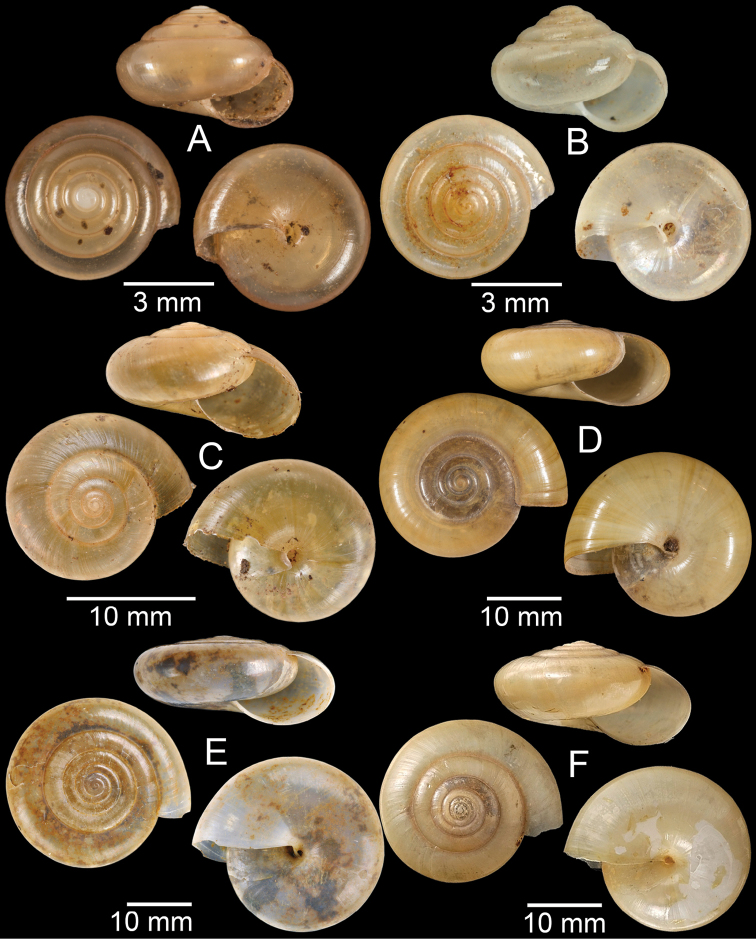
**A, B***Microcystinamessageri***A** syntype MNHN-IM-2000-9658 and **B** CUMZ collection **C***Sarikabenoiti*, CUMZ collection **D, E***Sarikadespecta***D** syntype MNHN-IM-2000-27882 and **E** CUMZ collection **F***Sarikahainesi*, syntype NMHUK ex. Cuming collection.

Syntype MNHN-IM-2000-9658 from “Bac-Kan” (1 shell; Fig. 38A). Specimens from Ban Nong Kham village, Kasy District, Vientiane Province (Fig. 38B).

############### Distribution.

Laos and Vietnam (Saurin 1953, Schileyko 2011).

############### Remarks.

For the correct authorship of the name, see Wood and Gallichan (2008: 66).

############## Microcystina mirmido

Taxon classificationAnimaliaStylommatophoraAriophantidae

(Dautzenberg, 1893)

Microcystis mirmido Dautzenberg, 1893: 162, 163, pl. 8, fig. 1. Type locality: environs ďHaïphong [the area of Hai Phong Province, Vietnam]. Saurin 1953: 113.
Microcystina mirmido : Schileyko 2011: 33.

############### Distribution.

Laos and Vietnam (Saurin 1953, Schileyko 2011)

############### Remarks.

No material of this species was found.

############# *Otesia* Adams, 1856

############## Otesia mecongana

Taxon classificationAnimaliaStylommatophoraAriophantidae

Möllendorff, 1899

Otesia mecongana Möllendorff, 1899: 165. Type locality: Oberer Mekong im Lande der Laos [upper Mekong in Laos].

############### Distribution.

Known only from the type locality in Laos (Möllendorff 1899).

############### Remarks.

No material of this species was found.

############# *Parmarion* Fischer, 1855

############## Parmarion martensi

Taxon classificationAnimaliaStylommatophoraAriophantidae

Simroth, 1893

Parmarion martensi Simroth, 1893: 107, 108, pl. 7, fig. 8, pl. 8, figs 20–22. Type locality: Cambodja [Cambodia]. Maassen 2001: 108.

############### Material examined.

Specimens from limestones in Ngoy Town, Ngoy District, Luang Phrabang Province (Figs 19G, 57D).

############### Distribution.

Cambodia, Malaysia and Singapore (Maassen 2001).

############# *Sarika* Godwin-Austen, 1907

############## Sarika benoiti

Taxon classificationAnimaliaStylommatophoraAriophantidae

(Crosse & Fischer, 1863)

Zonites benoiti Crosse & Fischer, 1863b: 346, pl. 14, fig. 4. Type locality: in loco Fuyen-Moth dicto, Cochinchine [Phu Yen Province, Vietnam].
Macrochlamys benoiti : Saurin 1953: 113. Schileyko 2011: 30, 31.

############### Material examined.

Specimens from Phou Fa Mountain, Phongsaly District, Phongsaly Province (Fig. 38C).

############### Distribution.

Cambodia, Laos, Thailand and Vietnam (Saurin 1953, Schileyko 2011).

############## Sarika despecta

Taxon classificationAnimaliaStylommatophoraAriophantidae

(Mabille, 1887)

Nanina despecta Mabille, 1887a: 2. Type locality: Tonkin [North Vietnam]. Mabille 1887b: 79, 80, pl. 1, figs 13–14.
Macrochlamys despecta : Schileyko 2011: 31.

############### Material examined.

Syntypes MNHN-IM-2000-27880 from “Nha Trang” (1 shell), MNHN-IM-2000-27881 from “Nha Trang” (6 shells) and MNHN-IM-2000-27882 from “Nha Trang” (5 shells; Fig. 38D). Specimens from Ngoy Town, Ngoy District, Luang Phrabang Province (Figs 38E, 56H).

############### Distribution.

Vietnam (Schileyko 2011).

############## Sarika hainesi

Taxon classificationAnimaliaStylommatophoraAriophantidae

(Pfeiffer, 1856)

Helix hainesi Pfeiffer, 1856a: 32. Type locality: Siam [Thailand]. Pfeiffer 1856b: 75, 76, pl. 21, figs 1–3.
Macrochlamys hainesi : Saurin 1953: 113.
Sarika aff. hainesii [sic]: Solem 1966: 38, 39, fig. 5a.

############### Material examined.

Syntypes NMHUK ex. Cuming collection from “Siam” (3 shells; Fig. 38F).

############### Distribution.

Laos and Thailand (Saurin 1953, Solem 1966).

############### Remarks.

No material of this species was found, and only the type specimens were examined.

############## Sarika resplendens

Taxon classificationAnimaliaStylommatophoraAriophantidae

(Philippi, 1846)

Helix resplendens Philippi, 1846: 192. Type locality: Prope Mergui Indiae orientalis [Myeik (Mergui) Archipelago, Myeik District, Tanintharyi Region, Myanmar].
Macrochlamys resplendens : Collinge 1903: 209.
Nanina (Austenia) resplendens : Kobelt 1905: 1183, 1184, pl. 294, figs 1, 2.
Sarika resplendens : Blanford and Godwin-Austen 1908: 277, 278, fig. 84. Schileyko 2011: 34.

############### Material examined.

**Figure 39.**
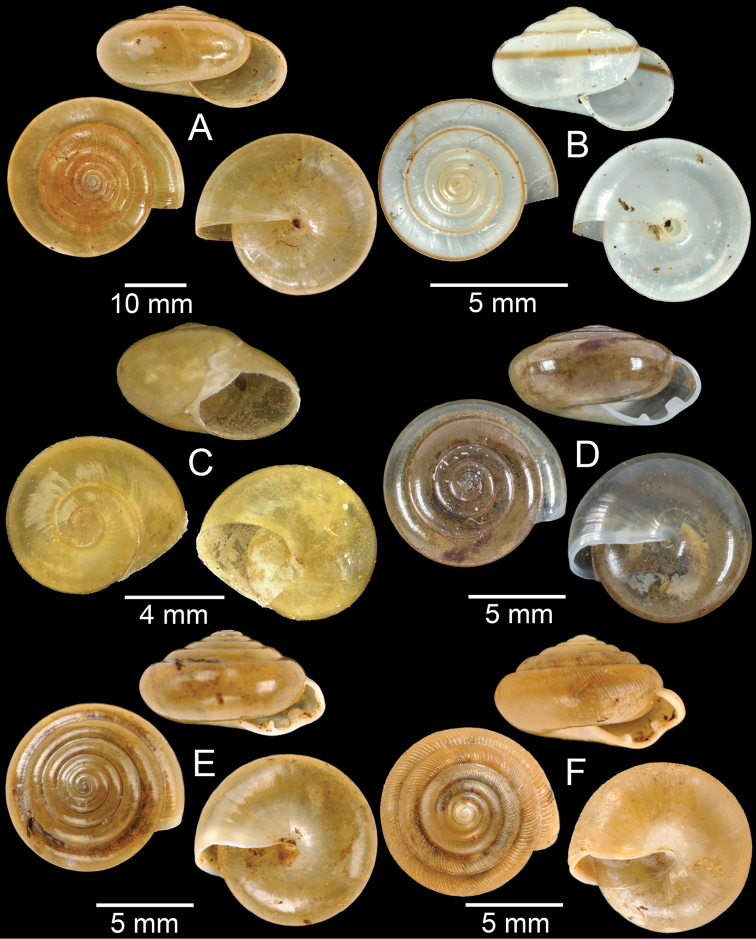
**A***Sarikaresplendens*, CUMZ collection **B***Chalepotaxisinfantilis*, CUMZ collection **C***Durgellarhaphiellus*, syntype ZMB/Moll-5033 **D, E***Sesarabouyei***D** syntype MNHN-IM-2000-27879 and **E** CUMZ collection **F***Sesarapenoti*, CUMZ collection.

Specimens from Ban Namone village, Xayaboury District, Xayaboury Province (Figs 39A, 57A).

############### Distribution.

mainland Southeast Asia and eastern India (Collinge 1903, Schileyko 2011).

############ Family Helicarionidae Bourguignat, 1877

############# *Chalepotaxis* Ancey, 1887

############## Chalepotaxis infantilis

Taxon classificationAnimaliaStylommatophoraHelicarionidae

(Gredler, 1881)

Helix similaris var. infantilis Gredler, 1881: 111. Type locality: Provinz Hunan, Distrikt Yün-tscheu-fu, China [Yueyanglou District, Hunan Province, China].
Nanina (?) infantilis: Gredler 1884: 143, pl. 3, figs 2, 7–10.
Xesta unilineata Dautzenberg, 1893: 161, pl. 7, fig. 4. Type locality: environs ďHaïphong [the area of Hai Phong Province, Vietnam]. Schileyko 2011: 41.
Chalepotaxis infantilis : Saurin 1953: 113. Zilch 1968: 157. Schileyko 2011: 41. Páll-Gergely et al. 2017a: 114–116, figs 1–4.
Bradybaena similaris infantilis : Richardson 1983: 37.

############### Material examined.

Paratype SMF 193148 from “Hunan, China” (1 shell). Specimens from Nam Ork Roo, Ban Nathong village, Namo District, Oudomxay Province (Fig. 39B).

############### Distribution.

China, Laos and Vietnam (Saurin 1953, Schileyko 2011, Páll-Gergely et al. 2017a).

############# *Durgella* Blanford, 1863

############## Durgella libas

Taxon classificationAnimaliaStylommatophoraHelicarionidae

Solem, 1966

Durgella libas Solem, 1966: 50–56, figs 7–9, 13b. Type locality: Wang Dao, North Thailand [Chiang Dao District, Chiang Mai Province, Thailand].

############### Material examined.

Specimens from Khaungsi waterfall, Luang Phrabang District, Luang Phrabang Province (Figs 19F, 57C).

############### Distribution.

Known only from the type locality in Northern Thailand (Solem 1966).

############## Durgella rhaphiellus

Taxon classificationAnimaliaStylommatophoraHelicarionidae

(Martens, 1867)

Helicarion rhaphiellus Martens, 1867: 69, pl. 12, fig. 9. Type locality: Siam [Thailand]. Saurin 1953: 113.

############### Material examined.

Syntype ZMB/Moll–5033 from “Siam” (1 shell + one decayed; Fig. 39C).

############### Distribution.

Laos and Thailand (Martens 1867, Saurin 1953).

############### Remarks.

No material of this species was found, and only the type specimen was examined.

############# *Sesara* Albers, 1860

############## Sesara bouyei

Taxon classificationAnimaliaStylommatophoraHelicarionidae

(Crosse & Fischer, 1863)

Helix bouyei Crosse & Fischer, 1863a: 269, 270, pl. 9, fig. 7. Type locality: insula Poulo-Condor [Con Dao Islands, Ba Ria–Vung Tau Province, Vietnam].
Sesara bouyei : Schileyko 2011: 34.

############### Material examined.

Syntype MNHN-IM-2000-27879 from “Poulo Condor” (1 shell; Fig. 39D). Specimens from Hot Spring, Kham District, Xieng Khaung Province (Fig. 39E).

############### Distribution.

Vietnam (Schileyko 2011).

############## Sesara penoti

Taxon classificationAnimaliaStylommatophoraHelicarionidae

Ancey, 1898

Sesara penoti Ancey, 1898: 129, 130, pl. 9, fig. c. Type locality. Luang-prabang [Luang Phrabang Province, Laos]. Saurin 1953: 113. Wood and Gallichan 2008: 74, pl. 16, figs 2, ii (label).

############### Material examined.

Syntype NMW 1955.158.24179 from “Luang-prabang, Laos” (1 shell). Specimens from Nam Ork Roo, Ban Nathong village, Namo District, Oudomxay Province (Fig. 39F).

############### Distribution.

Laos (Ancey 1898, Saurin 1953).

############# *Sitala* Adams, 1865

############## Sitala tricincta

Taxon classificationAnimaliaStylommatophoraHelicarionidae

Saurin, 1953

Sitala tricincta Saurin, 1953: 118, pl. 4, fig. 8a, b. Type locality: environs du village méo de Pah Hia, à 100 kilomètres au Sud de Xieng-Khouang, chef-lieu de la province du Tran Ninh, Laos [probably refers to Ban Namthong, Longchaeng District, Xaisomboun Province, Laos].

############### Distribution.

Known only from the type locality in Laos (Saurin 1953).

############### Remarks.

No material of this species was found, and the type specimen could not be traced. This species was figured in Saurin (1953: pl. 4, fig. 8a; see Fig. 17H). For the current interpretation of Pa Hia, see Páll-Gergely et al. (2016: 13).

########## Infraorder Helicoidei [= “Helicoid Clade”]

########### Superfamily Helicoidea

############ Family Camaenidae Pilsbry, 1895

############# Subfamily Bradybaeninae Pilsbry, 1934

############## *Aegista* Albers, 1850

############### Aegista coudeini

Taxon classificationAnimaliaStylommatophoraCamaenidae

(Bavay & Dautzenberg, 1900)

Helix (Ganesella) coudeini Bavay & Dautzenberg, 1900b: 113. Type locality: Bac-Kan [Bac Kan Province, Vietnam]. Bavay and Dautzenberg 1900a: 443, 444, pl. 9, figs 13–15.
Ganesella coudeini : Richardson 1985: 134. Schileyko 2011: 48.
Ganesella (Ganesella) coudeini : Solem 1966: 101.

################ Material examined.

**Figure 40.**
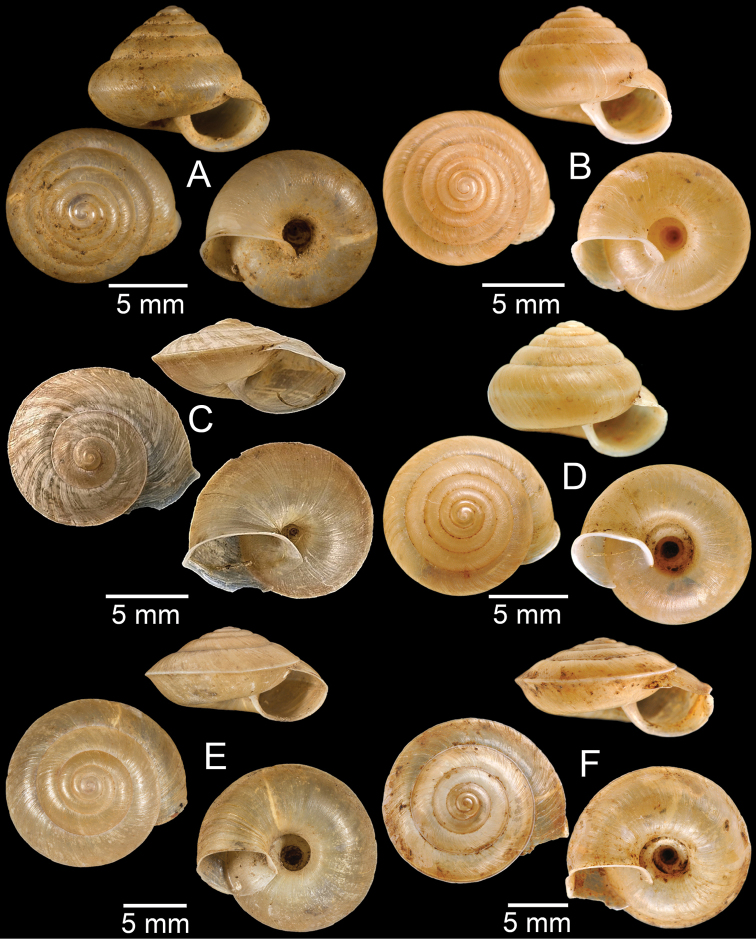
**A, B***Aegistacoudeini***A** syntype MNHN-IM-2000-1867 and **B** CUMZ collection **C***Aegistaemma*, syntype NHMUK ex. Cuming collection **D***Aegistagitaena*, CUMZ collection **E, F***Aegistapseudotrochula***E** syntype MNHN-IM-2000-31775 and **F** CUMZ collection.

Syntype MNHN-IM-2000-1867 from “Bac-Kan, Tonkin” (1 shell; Fig. 40A). Specimens from Nam Noua bridge, Viengxay District, Houaphanh Province (Fig. 40B).

################ Distribution.

Thailand and Vietnam (Solem 1966, Schileyko 2011).

############### Aegista emma

Taxon classificationAnimaliaStylommatophoraCamaenidae

(Pfeiffer, 1863)

Helix emma Pfeiffer, 1863a[1862]: 273. Type locality: Lao Mountains, Camboja [Cambodia or Laos]. Pfeiffer 1863b: 209, pl. 55, figs 4–7. Pilsbry 1888: 53, pl. 11, figs 59–61.
Aegista (Plectotropis) emma : Richardson 1983: 11.

################ Material examined.

Syntype NHMUK 20170016 from “Lao Mountains, Camboja” (1 shell; Fig. 40C). Specimens from Khaungsi waterfall, Luang Phrabang District, Luang Phrabang Province (Fig. 57E).

################ Distribution.

Laos and possibly in Cambodia (Pilsbry 1888).

############### Aegista gitaena

Taxon classificationAnimaliaStylommatophoraCamaenidae

(Bavay & Dautzenberg, 1909)

Helix (Plectotropis) gitaena Bavay & Dautzenberg, 1909d[1908]: 240. Type locality: Nat-Son [Nat Son Commune, Kim Boi District, Hoa Binh Province, Vietnam]. Bavay and Dautzenberg 1909b: 189, 190, pl. 7, figs 9–11.
Aegista (Plectotropis) gitaena : Richardson 1983: 11.
Plectotropis gitaena : Schileyko 2011: 39.

################ Material examined.

Specimens from Ban Namone village, Xayaboury District, Xayaboury Province (Fig. 40D).

################ Distribution.

Vietnam (Schileyko 2011).

############### Aegista pseudotrochula

Taxon classificationAnimaliaStylommatophoraCamaenidae

(Bavay & Dautzenberg, 1909)

Helix (Plectotropis) pseudotrochula Bavay & Dautzenberg, 1909d[1908]: 239. Type locality: Muong-Kong [Muong Khuong District, Lao Cai Province, Vietnam], Muong-Hum [Muong Hum Commune, Bat Xat District, Lao Cai Province, Vietnam], Pac-Kha [Pa Kha in Long Luong Commune, Van Ho District, Son La Province, Vietnam], Phong-Tho [Phong Tho District, Lai Chau Province, Vietnam], Trinh-Tuong [Trinh Tuong Commune, Bat Xat District, Lao Cai Province, Vietnam]. Bavay and Dautzenberg 1909b: 188, 189, pl. 7, figs 6–8.
Aegista (Plectotropis) pseudotrochula : Richardson 1983: 15.
Plectotropis pseudotrochula : Schileyko 2011: 39.

################ Material examined.

Syntype MNHN-IM-2000-31775 (1 shell; Fig. 40E). Specimens from limestone hills at Ban Oudom village, Pakbeg Ditrict, Oudomxay Province (Fig. 40F).

################ Distribution.

Vietnam (Schileyko 2011).

############### Aegista subinflexa major

Taxon classificationAnimaliaStylommatophoraCamaenidae

(Bavay & Dautzenberg, 1909)

Helix (Plectotropis) subinflexavar.major Bavay & Dautzenberg, 1909b: 188, pl. 7, fig. 4. Type locality: Phong-Tho [Phong Tho District, Lai Chau Province, Vietnam].
Aegista (Plectotropis) subinflexamajor : Richardson 1983: 17.
Plectotropis subinflexa var. major : Schileyko 2011: 39.

################ Material examined.

**Figure 41.**
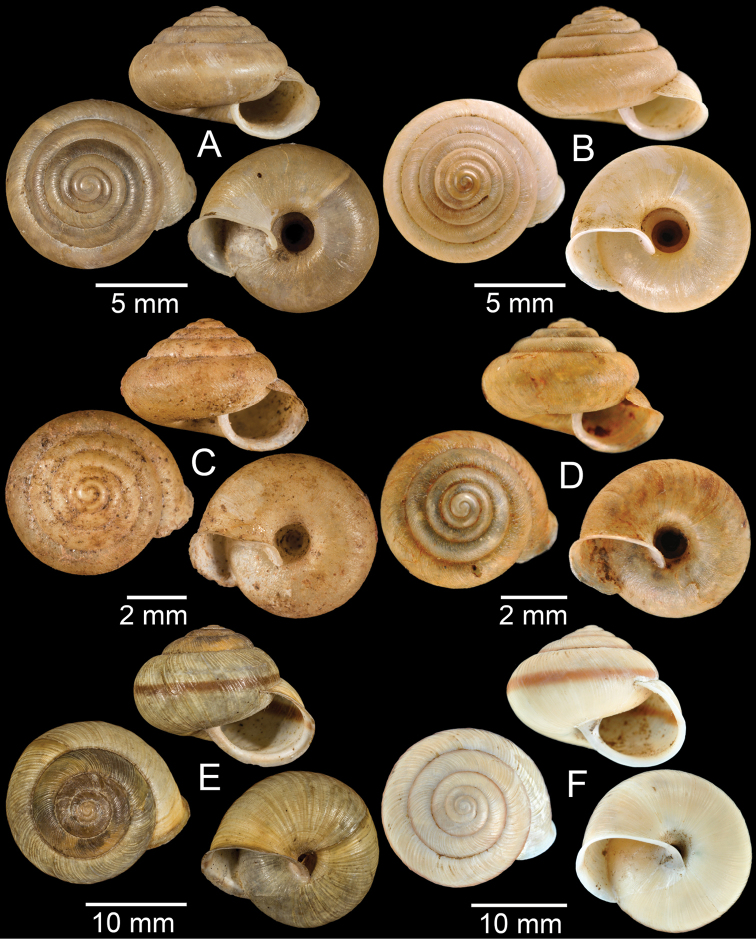
**A, B***Aegistasubinflexamajor***A** syntype MNHN-IM-2000-31777 and **B** CUMZ collection **C, D***Aegistasubinflexaminor***C** syntype MNHN-IM-2000-31778 and **D** CUMZ collection **E, F***Bradybaenabocageana***E** syntype MNHN-IM-2000-1844 and **F** CUMZ collection.

Syntype of “var. *major* Bavay & Dautzenberg, 1909” MNHN-IM-2000-31777 from “Phong-Tho” (1 shell; Fig. 41A). Specimens from Ban Namone village, Xayaboury District, Xayaboury Province (Fig. 41B).

################ Distribution.

Vietnam (Schileyko 2011).

############### Aegista subinflexa minor

Taxon classificationAnimaliaStylommatophoraCamaenidae

(Bavay & Dautzenberg, 1909)

Helix (Plectotropis) subinflexavar.minor Bavay & Dautzenberg, 1909b: 188, pl. 7, fig. 5. Type locality: Long-Ping, près de Pac-Kha; Phong-Tho, Muong-Hum [Lung Phinh Commune, Bac Ha District, Lao Cai Province; Muong Hum Commune, Bat Xat District, Lao Cai Province, Vietnam].
Aegista (Plectotropis) subinflexaminor : Richardson 1983: 17.
Plectotropis subinflexa var. minor : Schileyko 2011: 39.

################ Material examined.

Syntype of “var. *minor* Bavay & Dautzenberg, 1909” MNHN-IM-2000-31778 from “Phong-Tho, Muong-Hum” (1 shell; Fig. 41C). Specimens from Tam Xang Cave, Ban Nam Kha village, Kham District, Xieng Khaung Province (Fig. 41D).

################ Distribution.

Vietnam (Schileyko 2011).

############## *Bradybaena* Beck, 1837

############### Bradybaena bocageana

Taxon classificationAnimaliaStylommatophoraCamaenidae

(Crosse, 1864)

Helix bocageana Crosse, 1864: 284, 285. Type locality: China. Crosse 1866: 58, 59, pl. 1, fig. 4.
Bradybaena (Karaftohelix) weyrichibocageana : Richardson 1983: 45.
Karaftohelix bocageana bocageana : Sysoev and Schileyko 2009: 180, fig. 102a.

################ Material examined.

Syntype MNHN-IM-2000-1844 from “China” (1 shell; Fig. 41E). Specimens from Hot Spring, Ban Nam Hom village, Kham District, Xieng Khaung Province (Fig. 41F).

################ Distribution.

China and Russia (Sysoev and Schileyko 2009).

############### Bradybaena jourdyi

Taxon classificationAnimaliaStylommatophoraCamaenidae

(Morlet, 1886)

Helix jourdyi Morlet, 1886c: 75, 76. Type locality: Tonkin. Les environs de Lang-son, Dang-son et Chu [Chu River, Lang Son Province; Dang Son Commune, Do Luong District, Nghe An Province, Vietnam]. Morlet 1887[1886]: 258, 269, 270, pl. 12, figs 3, 3a, b.
Bradybaena jourdyi : Richardson 1983: 30.
Bradybaena (?) jourdyi: Schileyko 2011: 40.

################ Material examined.

**Figure 42.**
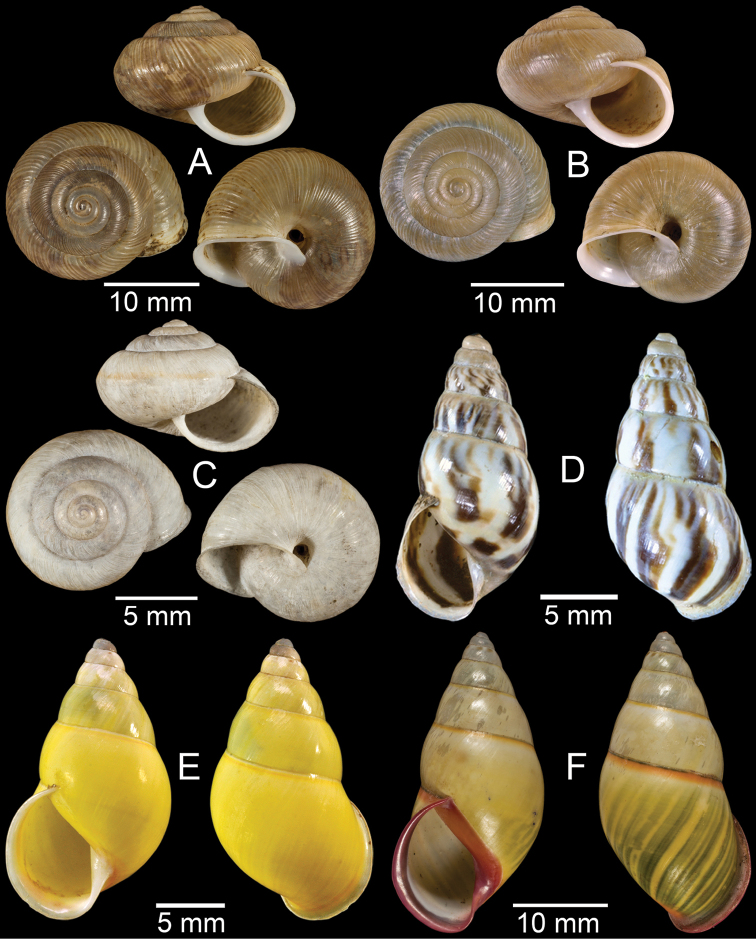
**A, B***Bradybaenajourdyi***A** syntype MNHN-IM-2000-1944 and **B** CUMZ collection **C***Bradybaenasimilaris*, syntype MNHN-IM-2000-31776 **D***Amphidromusareolatus*, specimen CUMZ 7022 **E***Amphidromusflavus*, specimen CUMZ 7029 **F***Amphidromusfuscolabris*, holotype of “*thakhekensis*” MNHN-IM-2000-33216.

Syntype MNHN-IM-2000-1944 from “Lang-Son, Dang-Son et Chu” (1 shell; Fig. 42A). Specimens from Tam Pew Cave, Kham District, Xieng Khaung Province (Fig. 42B).

################ Distribution.

Vietnam (Schileyko 2011).

############### Bradybaena similaris

Taxon classificationAnimaliaStylommatophoraCamaenidae

(Fèrussac, 1821)

Helix similaris Fèrussac, 1821: 43, no. 262, pl. 25b, figs 1, 4. Type locality: Timor.
Eulota similaris : Gude 1914: 200–202.
Bradybaena similaris : Richardson 1983: 36–38. Schileyko 2011: 40.

################ Material examined.

Syntypes MNHN-IM-2000-31776 “Timor ? Nouvelle Hollande” (3 shells; Fig. 42C).

################ Distribution.

This species has been introduced widely around the world (Schileyko 2011).

################ Remarks.

This species occurs in disturbed lowland to highland in Luang Phrabang, Phongsali and Xayaboury Provinces of Laos.

############## *Plectotropis* Martens, 1860

############### Plectotropis bonnieri

Taxon classificationAnimaliaStylommatophoraCamaenidae

(Fischer, 1898)

Helix (Plectotropis ?) bonnieri Fischer, 1898: 319, 320, pl. 17, figs 12–16. Type locality: Rochers calcaires Dèo-Ma-Phuc [Deo Ma Phuc limestone hills located east of Cao Bang District, Cao Bang Province, Vietnam].
Plectotropis bonnieri : Saurin 1953: 113. Schileyko 2011: 38.

################ Distribution.

Laos and Vietnam (Saurin 1953, Schileyko 2011).

################ Remarks.

No material of this species was found.

############### Plectotropis repanda

Taxon classificationAnimaliaStylommatophoraCamaenidae

(Pfeiffer, 1861)

Helix repanda Pfeiffer, 1861a: 195. Type locality: Camboja [Cambodia].
Plectotropis (?) repanda: Schileyko 2011: 39.

################ Distribution.

Cambodia, Laos, Thailand and Vietnam (Schileyko 2011).

################ Remarks.

No material of this species was found.

############# Subfamily Camaeninae Pilsbry, 1895

############## *Amphidromus* Albers, 1850

############### Amphidromus areolatus

Taxon classificationAnimaliaStylommatophoraCamaenidae

(Pfeiffer, 1861)

Bulimus areolatus Pfeiffer, 1861a: 194. Type locality: Siam [Thailand]. Pfeiffer 1861b: 172, 173, pl. 46, figs 11, 12.
Amphidromus (Syndromus) areolatus : Laidlaw and Solem 1961: 564, 600, 601. Inkhavilay et al. 2017: 20–24, figs 7g–i, 9a, 10a–c, 11a–b.
Amphidromus areolatus : Sutcharit et al. 2015: 58, fig. 3j, k.

################ Material examined.

Lectotype NHMUK 19601430 and paralectotype NHMUK 19601431 (1 shell) figured in Sutcharit et al. (2015: fig. 3j, k). Specimen CUMZ 7022 from Tad Fek waterfall, Sammakeexay District, Attapeu Province (Figs 42D, 57F).

################ Distribution.

Laos and Thailand (Laidlaw and Solem 1961, Inkhavilay et al. 2017).

############### Amphidromus comes

Taxon classificationAnimaliaStylommatophoraCamaenidae

(Pfeiffer, 1861)

Bulimus comes Pfeiffer, 1861a: 193, 194. Type locality: Camboja [Cambodia]. Pfeiffer 1866: 311, 312, pl. 75, figs 10, 11.
Amphidromus (Amphidromus) comes : Laidlaw and Solem 1961: 531, 532, 610.
Amphidromus comes : Schileyko 2011: 50. Sutcharit et al. 2015: 64, fig. 5g, h.

################ Material examined.

Lectotype NHMUK 19601434 and paralectotypes NHMUK 19601435 (2 shells) figured in Sutcharit et al. (2015: fig. 5g, h).

################ Distribution.

Cambodia, Laos, Thailand and Vietnam (Laidlaw and Solem 1961, Schileyko 2011).

################ Remarks.

No material of this species was found, and only the type specimens were examined.

############### Amphidromus flavus

Taxon classificationAnimaliaStylommatophoraCamaenidae

(Pfeiffer, 1861)

Bulimus flavus Pfeiffer, 1861a: 194. Type locality: Siam [Thailand]. Pfeiffer 1861b: 171, 172, pl. 46, figs 7, 8.
Amphidromus (Syndromus) flavus : Laidlaw and Solem 1961: 563, 564, 619. Inkhavilay et al. 2017: 24, figs 9b, 10e–k, 11c–d, 12a–c.
Syndromus flavus : Schileyko 2011: 51.
Amphidromus flavus : Sutcharit et al. 2015: 70, fig. 7i, j.

################ Material examined.

Lectotype NHMUK 19601436 and paralectotype NHMUK 19601437 (1 shell) figured in Sutcharit et al. (2015: fig. 7i, j). Specimens CUMZ 7029 from Tam Pou Kham, Vangvieng District, Vientiane (Fig. 42E).

################ Distribution.

Laos, Malaysia, Thailand and Vietnam (Laidlaw and Solem 1961, Inkhavilay et al. 2017).

############### Amphidromus fuscolabris

Taxon classificationAnimaliaStylommatophoraCamaenidae

Möllendorff, 1898

Amphidromus zebrinus fuscolabris Möllendorff, 1898: 75. Type locality: Boloven [Boloven Plateau, Paksong District, Champasak Province, Laos]. Schileyko 2011: 52.
Amphidromus (Syndromus) zebrinusfuscolabris : Zilch 1953b: 134, pl. 23, fig. 22. Laidlaw and Solem 1961: 564, 621.
Amphidromus (Syndromus) fuscolabris : Inkhavilay et al. 2017: 32, figs 9e–f, 12g–i, 13i–m, 14c–d.
Amphidromus thakhekensis Thach & Huber in Thach, 2017: 48, figs 553–556. Type locality: Thakhek, Khammouane Province, South-Central Laos. New synonym.

################ Material examined.

**Figure 43.**
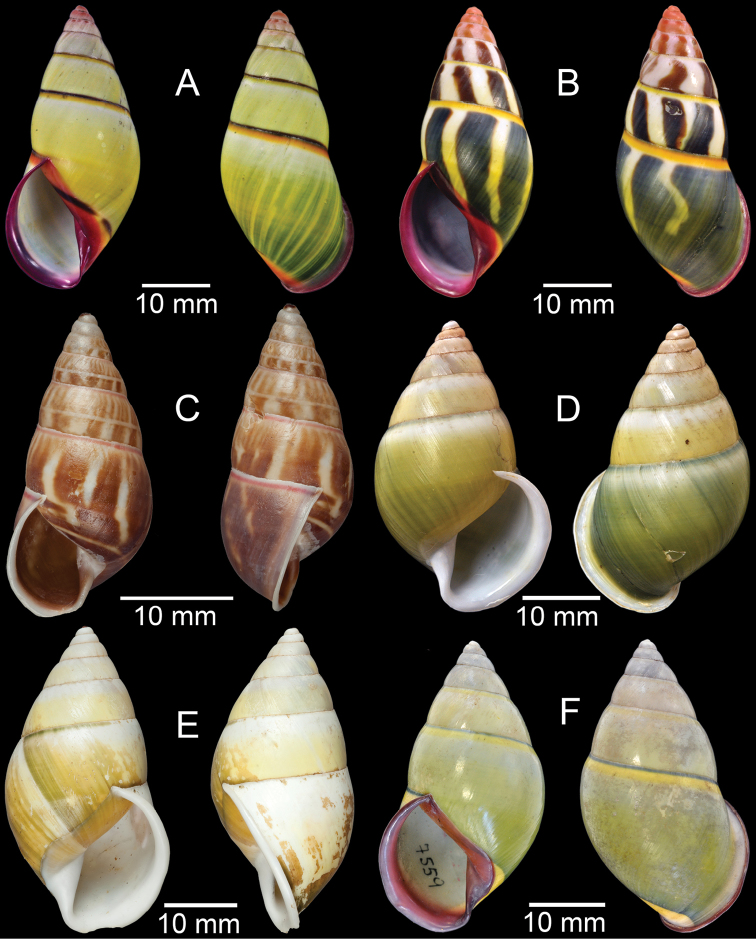
**A, B***Amphidromusfuscolabris***A** specimen CUMZ 7040 and **B** specimen CUMZ 7042 **C***Amphidromusgerberi*, holotype FMNH 381987 **D, E***Amphidromusgivenchyi***D** CUMZ collection and **E** holotype of “*richgoldbergi*” FMNH 381986 **F***Amphidromushaematostoma*, lectotype of “var. *viridis*” SMF 7559.

Holotype of “*fuscolabris* Möllendorff, 1898” SMF 7641 figured in Inkhavilay et al. (2017: fig. 13i) and holotype of “*thakhekensis* Thach & Huber, 2017” MNHN-IM-2000-33216 (Fig. 42F). Specimens CUMZ 7040, 7042 from Ban Phone village, Lamam District, Sekong Province (Figs 43A, B, 57G, H).

################ Distribution.

Known from several localities in Laos and probably in Vietnam (Schileyko 2011, Inkhavilay et al. 2017, Thach 2017).

################ Remarks.

The holotype of *Amphidromusthakhekensis* Thach & Huber, 2017 is identical to the un-banded colour form of *A.fuscolabris*, which probably reflects intra-population variation rather than separate biological species entities. Therefore, we recognised this name as a junior synonym of *A.fuscolabris*.

############### Amphidromus gerberi

Taxon classificationAnimaliaStylommatophoraCamaenidae

Thach & Huber, 2017

Amphidromus gerberi Thach & Huber in Thach, 2017: 39, 40, figs 649–652, 654, 655. Type locality: Don Khong Island on Mekong River, Si Phan Don, South Laos [Khong District, Champasak Province, Laos].

################ Material examined.

Holotype FMNH 381987 (Fig. 43C).

################ Distribution.

Known only from the type locality in Laos (Thach 2017).

################ Remarks.

No material of this species was found, and only the type specimens were examined.

############### Amphidromus givenchyi

Taxon classificationAnimaliaStylommatophoraCamaenidae

Geret, 1912

Amphidromus givenchyi Geret, 1912: 55, 56, pl. 2, figs 21, 22. Type locality: unknown. Laidlaw and Solem 1961: 526, 621.
Amphidromus (Amphidromus) givenchyi : Sutcharit and Panha 2006: 26–28, figs 4n–q, 18, 19. Inkhavilay et al. 2017: 14–15, figs 2c, 3c, 4i.
Amphidromus richgoldbergi Thach & Huber in Thach, 2017: 45, figs 505–508. Type locality: Vang Vieng, Ventiane Province, Central Laos [Vangvieng District, Vientiane Province, Laos]. New synonym.

################ Material examined.

Holotype of “*richgoldbergi* Thach & Huber, 2017” FMNH 381986 (Fig. 43E). Specimens CUMZ 7015 (Fig. 43D) from Tad Lor waterfall, Salavan District, Salavan Province.

################ Distribution.

Laos and Thailand (Sutcharit and Panha 2006, Inkhavilay et al. 2017).

################ Remarks.

*Amphidromusrichgoldbergi* Thach & Huber, 2017 has a dextral shell, yellowish-green periostracum and a brownish spot on the apex (Thach 2017). These characters are the intraspecific variations of *A.givenchyi* (Sutcharit and Panha 2006, Inkhavilay et al. 2017) rather than indicative of different biological species entities. Therefore, we recognised this name as a junior synonym of *A.givenchyi*.

############### Amphidromus haematostoma

Taxon classificationAnimaliaStylommatophoraCamaenidae

Möllendorff, 1898

Amphidromus haematostoma Möllendorff, 1898: 74, 75. Type locality: Boloven [Boloven Plateau, Champasak, Laos]. Schileyko 2011: 51.
Amphidromus haematostoma var. viridis Möllendorff, 1898: 75. Type locality: Boloven [Boloven Plateau, Champasak, Laos]. Laidlaw and Solem 1961: 670. Schileyko 2011: 51.
Amphidromus haematostoma var. varians Möllendorff, 1898: 75. Type locality: Boloven [Boloven Plateau, Champasak, Laos]. Laidlaw and Solem 1961: 668. Schileyko 2011: 51.
Amphidromus (Syndromus) haematostoma : Zilch 1953b: 132, pl. 22, figs 4, 5. Inkhavilay et al. 2017: 34, 35, fig. 13o–r.
Amphidromus haematostomus [sic]: Laidlaw and Solem 1961: 527, 625.
Amphidromus attapeuensis Thach & Huber in Thach, 2017: 37, 38, figs 573–578. Type locality: Attapeu Province, southeast of Laos, close to Vietnam border. New synonym.

################ Material examined.

**Figure 44.**
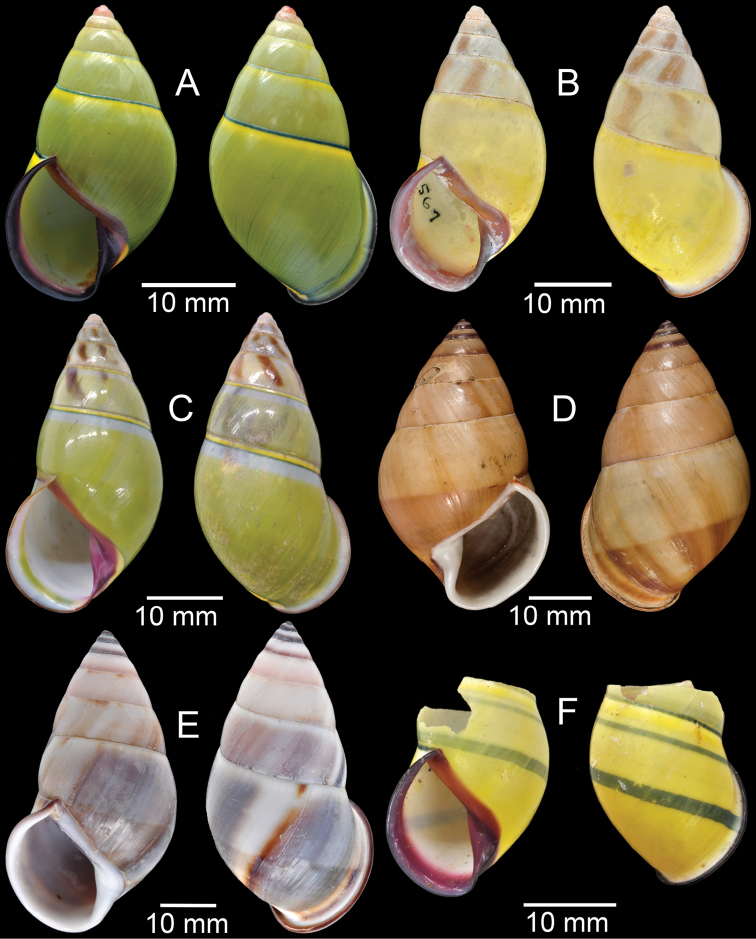
**A–C***Amphidromushaematostoma***A** CUMZ collection **B** lectotype of “var. *varians*” SMF 7561 and **C** holotype of “*attapeuensis*” NHMUK 20170278 **D, E***Amphidromusinversusannamiticus***D** syntype MNHN-IM-2000-1820 and **E** CUMZ collection **F***Amphidromuskhammouanensis*, holotype NHMUK 20170276.

Lectotype of “var. viridis Möllendorff, 1898” SMF 7559 (Fig. 43F), lectotype of “var. varians Möllendorff, 1898” SMF 7561 (Fig. 44B), and holotype of “*attapeuensis* Thach & Huber, 2017” NHMUK 20170278 (Fig. 44C). Specimens from Xe Pian village, Paksong District, Champasak Province (Fig. 44A).

################ Distribution.

Laos, Thailand and probably in Vietnam (Schileyko 2011, Inkhavilay et al. 2017, Thach 2017).

################ Remarks.

The holotype of *Amphidromusattapeuensis* Thach & Huber, 2017 has a greenish shell, purple lip and parietal callus, and brownish streaks on the apical whorls (Thach 2017). These characters are identical to that of the banded colour form of *A.haematostoma* (see Inkhavilay et al. 2017). Therefore, we recognised this name as a junior synonym of *A.haematostoma*.

############### Amphidromus inversus annamiticus

Taxon classificationAnimaliaStylommatophoraCamaenidae

(Crosse & Fischer, 1863)

Bulimus annamiticus Crosse & Fischer, 1863b: 357–359. Type locality: in vicinio urbis Saigon et pagi Fuyen-Moth dicti [Ho Chi Min Province and Phu Yen Province, Vietnam]. Crosse and Fischer 1864: 329, pl. 12, fig. 8.
Amphidromus inversus annamiticus : Laidlaw and Solem 1961: 561, 600. Schileyko 2011: 50.
Amphidromus (Amphidromus) inversusannamiticus : Sutcharit and Panha 2006: 9–14, figs 3e–h, 7d–f, 9.

################ Material examined.

Syntypes MNHN-IM-2000-1820 from “Saigon et Fuyen-Moth” (2 shells; Fig. 44D). Specimens from Don Sadam, Khong District, Champasak Province (Fig. 44E).

################ Distribution.

Cambodia, Thailand and Vietnam (Laidlaw and Solem 1961, Schileyko 2011).

############### Amphidromus khammouanensis

Taxon classificationAnimaliaStylommatophoraCamaenidae

Thach & Huber, 2017

Amphidromus khammouanensis Thach & Huber in Thach, 2017: 41, figs 501–503. Type locality: Thakhek city, Khammouane Province, Central Laos.

################ Material examined.

Holotype NHMUK 20170276 (Fig. 44F).

################ Distribution.

Known only from the type locality in Laos (Thach 2017).

################ Remarks.

No material of this species was found, and only the type specimens were examined.

############### Amphidromus laosianus

Taxon classificationAnimaliaStylommatophoraCamaenidae

(Bavay, 1898)

Amphidromus laosianus Bavay, 1898: 15, 16, pl. 2, figs 1, 1a. Type locality: Khône, sur les bords du Mékong [on the banks of the Mekong River, Khone District, Champasak Province, Laos]. Laidlaw and Solem 1961: 526, 634.
Amphidromus laosianus var. albocaerulescens Bavay, 1898: 16, pl. 2, figs 2, 2a. Type locality: Khône, sur les bords du Mékong [on the banks of the Mekong River, Khone District, Champasak Province, Laos]. Laidlaw and Solem 1961: 526, 598.
Amphidromus (Amphidromus) laosianus : Inkhavilay et al. 2017: 10, fig. 4g.

################ Material examined.

**Figure 45.**
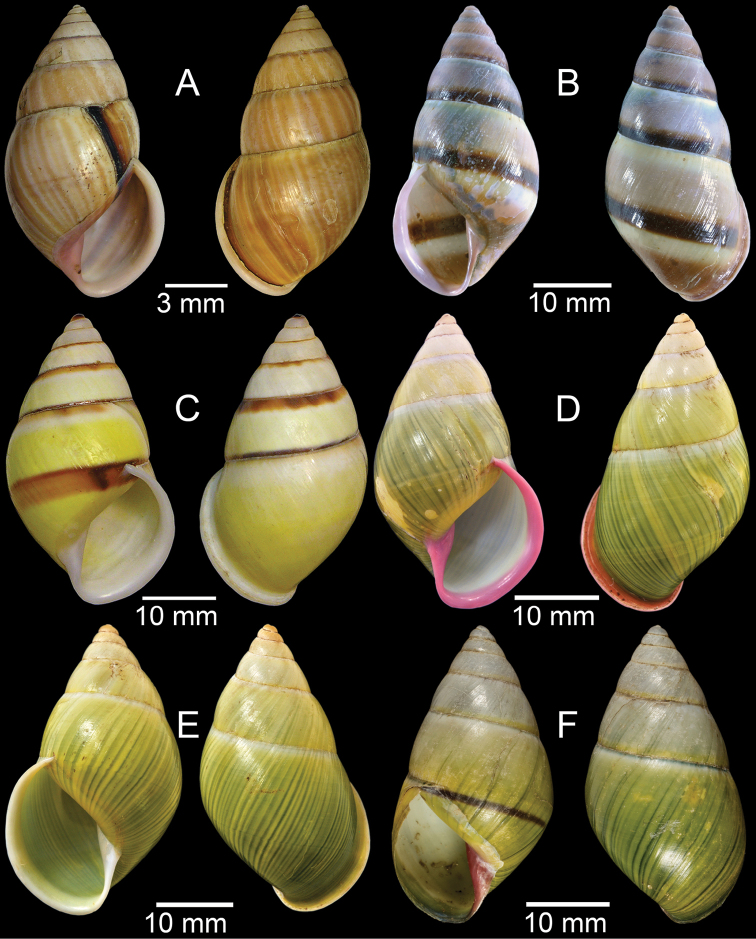
**A***Amphidromuslaosianus*, specimens RMNH 101049 **B***Amphidromuspervariabilis*, CUMZ collection **C***Amphidromusprotania*, holotype RMNH 98143 **D–F***Amphidromusroseolabiatus***D, E** CUMZ collection and **F** holotype of “*phuonglinhae*” MNHN-IM-2000-33200.

Specimens RMNH 101049 (2 shells) from Khone District, Champasak Province (Fig. 45A).

################ Distribution.

Known only from the type locality in Laos (Inkhavilay et al. 2017).

################ Remarks.

No material of this species was found, and only the old specimens were examined.

############### Amphidromus monsecourorum

Taxon classificationAnimaliaStylommatophoraCamaenidae

Thach & Huber, 2017

Amphidromus monsecourorum Thach & Huber in Thach, 2017: 43, figs 513–518. Type locality: Attapeu Province, southeast of Laos, close to Vietnam border.

################ Material examined.

Holotype RBINS-MT-3576 figured in Thach (2017: figs 513–515).

################ Distribution.

Known only from the type locality in Laos (Thach 2017).

################ Remarks.

No material of this species was found, and only the type specimens were examined.

############### Amphidromus pervariabilis

Taxon classificationAnimaliaStylommatophoraCamaenidae

Bavay & Dautzenberg, 1909

Amphidronus pervariabilis Bavay & Dautzenberg, 1909d[1908]: 246, 247. Type locality: Ban-Lao [Ban Lao in Muong Bum Commune, Thuan Chau District, Son La Province, Vietnam], Muong-Kong [Muong Khuong District, Lao Cai Province, Vietnam], Pha-Long [Pha Long Commune, Muong Khuong District, Lao Cai Province, Vietnam] and Pac Kha [Pa Kha Commune, Bac Ha District, Lao Cai Province, Vietnam]. Bavay and Dautzenberg 1909c: 279–281, pl. 9, figs 1–10, pl. 10, figs 1–8. Laidlaw and Solem 1961: 527, 528, 614, 648. Schileyko 2011: 50.
Amphidromus (Amphidromus) pervariabilis : Inkhavilay et al. 2017: 10, fig. 5a–l.

################ Material examined.

Syntypes MNHM-IM-2000-2049 (3 shells) figured in Inkhavilay et al. (2017: fig. 5a–b). Specimens from Ban Nam Lee village (km 34), Khoua District, Phongsaly Province (Fig. 45B).

################ Distribution.

Laos and Vietnam (Schileyko 2011, Inkhavilay et al. 2017)

############### Amphidromus protania

Taxon classificationAnimaliaStylommatophoraCamaenidae

Lehmann & Maassen, 2004

Amphidromus (Amphidromus) protania Lehmann & Maassen, 2004: 17–20, figs 1–4. Type locality: South Laos, Salavan Province, near the Ban Donxé village, the east bank of the Se Don River. Inkhavilay et al. 2017: 15, fig. 4h.

################ Material examined.

Holotype RMNH 98143 (Fig. 45C).

################ Distribution.

Laos (Inkhavilay et al. 2017).

################ Remarks.

No material of this species was found, and only the type specimens were examined.

############### Amphidromus roemeri

Taxon classificationAnimaliaStylommatophoraCamaenidae

(Pfeiffer, 1863)

Bulimus römeri Pfeiffer, 1863a[1862]: 274, pl. 36, fig. 4. Type locality: Laos Mountains, Camboja [Cambodia or Laos]. Pfeiffer 1863b: 217, pl. 57, figs 10, 11.
Amphidromus roemeri : Laidlaw and Solem 1961: 654. Sutcharit et al. 2015: 87, fig. 13e, f.
Amphidromus (Syndromus) roemeri : Inkhavilay et al. 2017: 27.

################ Material examined.

Lectotype NHMUK 19601450 and paralectotype NHMUK 19601451 (2 shells) figured in Sutcharit et al. (2015: fig. 13e, f).

################ Distribution.

Probably in Laos and Cambodia (Laidlaw and Solem 1961, Inkhavilay et al. 2017).

################ Remarks.

No material of this species was found, and only the type specimens were examined.

############### Amphidromus roseolabiatus

Taxon classificationAnimaliaStylommatophoraCamaenidae

Fulton, 1896

Amphidromus roseolabiatus Fulton, 1896: 89, pl. 6, fig. 8. Type locality: Siam [Thailand]. Laidlaw and Solem 1961: 527, 655. Sutcharit et al. 2015: 88, fig. 13j, k.
Amphidromus (Amphidromus) roseolabiatus : Inkhavilay et al. 2017: 3, 6, 9, 10, figs 2a, b, 3a, b, 4a–f, 6a, b, 7a–c.
Amphidromus phuonglinhae Thach, 2017: 45, figs 581–584. Type locality: Bo Trach District, Quang Binh Province, Central Vietnam. New synonym.

################ Material examined.

Lectotype of “*roseolabiatus* Fulton, 1896” NHMUK 19601462 figured in Sutcharit et al. (2015: fig. 13j) and holotype of “*phuonglinhae* Thach, 2017” MNHN-IM-2000-33200 (Fig. 45F). Specimens CUMZ 7011 from fruit orchards at Ban Phavong village, Yommalath District, Khammouan Province (Figs 45D, 58A) and CUMZ 7004 from Tam Mung Korn, Khamkeut District, Bolikhamxay Province (Fig. 45E).

################ Distribution.

Laos, Thailand and Vietnam (Inkhavilay et al. 2017, Thach 2017)

################ Remarks.

The holotype of *A.phuonglinhae* Thach, 2017 is an immature specimen. The distinguished characters of yellowish-green periostracum and reddish-brown spiral band and columella (see Thach 2017) are identical to the typical colour form of *A.roseolabiatus* (see Inkhavilay et al. 2017). Therefore, we recognised this name as a junior synonym of *A.roseolabiatus*.

############### Amphidromus semitessellatus

Taxon classificationAnimaliaStylommatophoraCamaenidae

(Morlet, 1885)

Bulimus (Amphidromus) semitessellatus Morlet, 1885[1884]: 387, 388, pl. 11, figs 2, 2a. Type locality: les montagnes qui bordent le grand fleuve au delà de Stung-Treng. Les forêts et les montagnes de Kampot à Compong-Som [Mountains and forest in Stung Treng, Kampot and Sihanoukville Provinces, Cambodia].
Amphidromus (Syndromus) semitessellatus : Laidlaw and Solem 1961: 564, 658. Inkhavilay et al. 2017: 27, 28, fig. 10l, m.
Syndromus semitessellatus : Schileyko 2011: 51, 52.

################ Material examined.

Lectotype MNHN-IM-2000-1985 figured in Inkhavilay et al. (2017: fig. 10l).

################ Distribution.

Cambodia, Laos, Thailand and probably in Vietnam (Laidlaw and Solem 1961, Schileyko 2011, Inkhavilay et al. 2017).

################ Remarks.

No material of this species was found, and only the type specimen was examined.

############### Amphidromus syndromoideus

Taxon classificationAnimaliaStylommatophoraCamaenidae

Inkhavilay & Panha, 2017

Amphidromus (Amphidromus) syndromoideus Inkhavilay & Panha in Inkhavilay et al. 2017: 16, 17, figs 2d, 3d, 4j, k, 6c, d, 7d–f. Type locality: Tam Nang Ann, Thakhek District, Khammouan Province, Laos.

################ Material examined.

**Figure 46.**
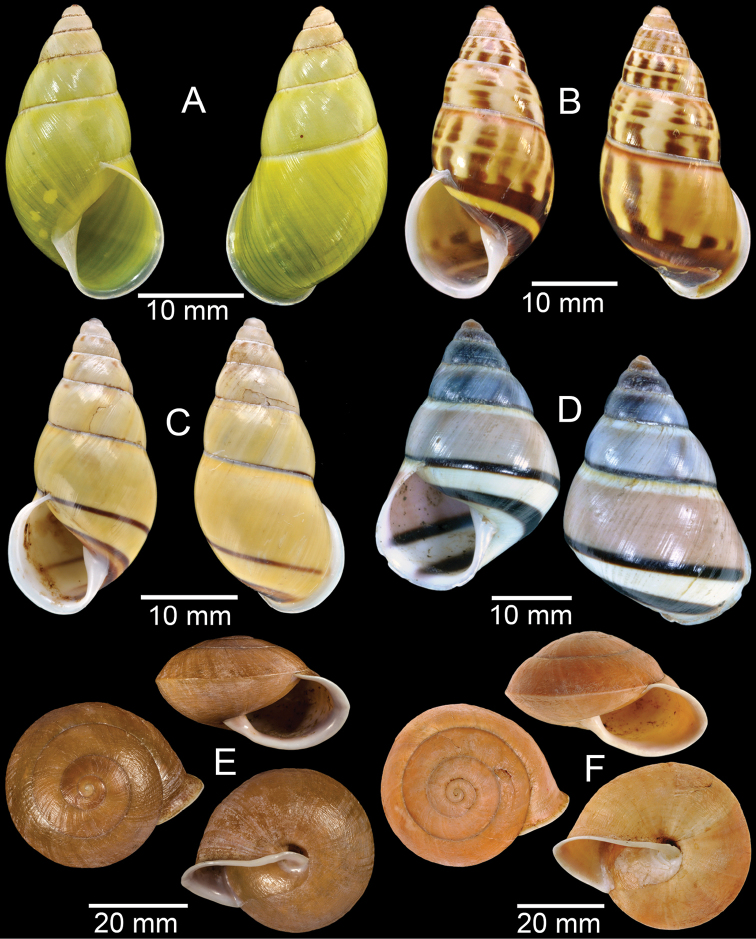
**A***Amphidromussyndromoideus*, holotype CUMZ 7019 **B, C***Amphidromusxiengensis*, CUMZ collection **D***Amphidromusxiengkhaungensis*, holotype CUMZ 7045 **E, F***Camaenachoboensis***E** syntype MNHN-IM-2000-1908 and **F** CUMZ collection.

Holotype CUMZ 7019 (Fig. 46A).

################ Distribution.

Known only from the type locality in Laos (Inkhavilay et al. 2017).

############### Amphidromus xiengensis

Taxon classificationAnimaliaStylommatophoraCamaenidae

Morlet, 1891

Amphidromus xiengensis Morlet, 1891b: 27. Type locality: Xieng-Mai et les forêts des bords du Ménam Pinh, Laos occidental [banks of Ping River, Chiang Mai Province, Thailand]. Morlet 1891a: 232, 240, 241, pl. 5, fig. 4. Solem 1966: 102, 103.
Amphidromus contrarius var. multifasciata Fulton, 1896: 78, pl. 7, fig. 5. Type locality: Cambodia. Laidlaw and Solem 1961: 642.
Amphidromus xiengensis var. clausus Pilsbry, 1900: 195, 196, pl. 63, figs 79–82. Type locality: Laos Mountains, Cambodia. Laidlaw and Solem 1961: 609.
Amphidromus (Syndromus) xiengensis : Laidlaw and Solem 1961: 564, 565, 672. Inkhavilay et al. 2017: 28, figs 9c, d, 12d–f, 13a–h, 14a, b.

################ Material examined.

Lectotype MNHN-IM-2000-5249 figured in Inkhavilay et al. (2017: fig. 13a). Specimens from Ban Na Deua village, Luang Phrabang District, Luang Phrabang Province (Fig. 46B, C).

################ Distribution.

Cambodia, Laos and Thailand (Laidlaw and Solem 1961, Solem 1966, Inkhavilay et al. 2017).

############### Amphidromus xiengkhaungensis

Taxon classificationAnimaliaStylommatophoraCamaenidae

Inkhavilay & Panha, 2017

Amphidromus (Syndromus) xiengkhaungensis Inkhavilay & Panha in Inkhavilay et al. 2017: 35, 36, fig. 13s, t. Type locality: Ban Nong Tang, Phou Kood District, Xieng Khaung Province, Laos.

################ Material examined.

Holotype CUMZ 7045 (Fig. 46D).

################ Distribution.

Known only from the type locality in Laos (Inkhavilay et al. 2017).

############### Amphidromus zebrinus

Taxon classificationAnimaliaStylommatophoraCamaenidae

(Pfeiffer, 1861)

Bulimus zebrinus Pfeiffer, 1861a: 194. Type locality: Siam [Thailand]. Pfeiffer 1861b: 172, pl. 46, figs 9, 10.
Amphidromus (Syndromus) zebrinus : Laidlaw and Solem 1961: 564, 673.
Syndromus zebrinus : Schileyko 2011: 52.
Amphidromus zebrinus : Sutcharit et al. 2015: 95, fig. 15k.

################ Material examined.

Lectotype NHMUK 19601439 figured in Sutcharit et al. (2015: fig. 15k).

################ Distribution.

Laos, Thailand and probably in Vietnam (Laidlaw and Solem 1961, Schileyko 2011).

################ Remarks.

No material of this species was found, and only the type specimen was examined.

############## *Camaena* Albers, 1850

############### Camaena choboensis

Taxon classificationAnimaliaStylommatophoraCamaenidae

(Mabille, 1889)

Helix choboensis Mabille, 1889: 7. Type locality: Tonkin.
Helix (Camaena) choboensis : Bavay and Dautzenberg 1909b: 173, 174.
Camaena choboensis : Schileyko 2011: 41.

################ Material examined.

Syntypes MNHN-IM-2000-1908 from “Tonkin” (2 shells; Fig. 46E). Specimens from limestone near Tam Tarn Kaison Cave, Viengxay District, Houaphanh Province (Fig. 46F).

################ Distribution.

Vietnam (Schileyko 2011).

############### Camaena huberi

Taxon classificationAnimaliaStylommatophoraCamaenidae

Thach, 2017

Camaena huberi Thach, 2017: 51, 52, figs 689–691. Type locality: south of Thakhek, Khammouane Province, Central Laos.

################ Material examined.

**Figure 47.**
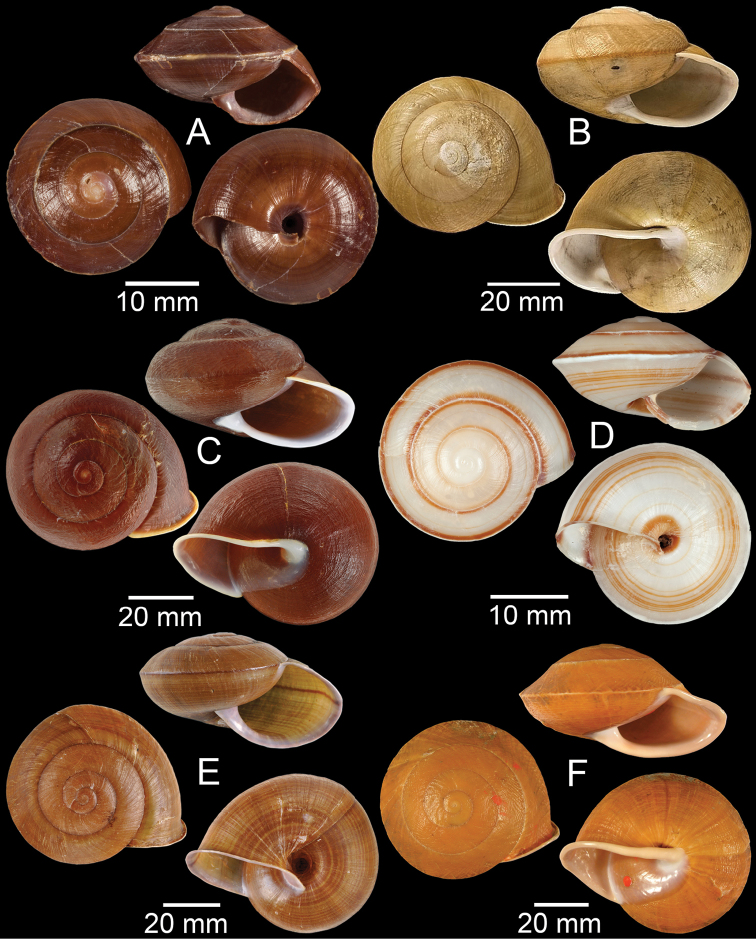
**A***Camaenahuberi*, holotype MNHN-IM-2000-33205 **B, C***Camaenaillustris***B** syntype NHMUK ex. Cuming collection and **C** CUMZ collection **D***Camaenaleeana*, holotype FMNH 381984 **E***Camaenasuprafusca*, CUMZ collection **F***Camaenavanbuensis*, holotype NHMUK 1896.1.25.1.

Holotype MNHN-IM-2000-33205 (Fig. 47A).

################ Distribution.

Known only from the type locality in Laos (Thach 2017).

################ Remarks.

This species was originally described based on the immature holotype, which have a relatively smaller size (width 26.3 mm, height 16.4 mm) and apertural lip not expanded. Therefore, this nominal species is possibly the young specimens of either *C.mansuyi* Dautzenberg & Fischer, 1906 or *C.suprafusca* Möllendorff, 1898, which are recorded from the massive karsts in Bolikhamxay and Khammouane Provinces.

############### Camaena illustris

Taxon classificationAnimaliaStylommatophoraCamaenidae

(Pfeiffer, 1863)

Helix illustris Pfeiffer, 1863a[1862]: 269, pl. 36, fig. 8. Type locality: Lao Mountains, Camboja [Cambodia or Laos]. Pfeiffer 1863b: 208, 209, pl. 55, figs 1–3.
Camaena illustris : Saurin 1953: 113. Richardson 1985: 74. Schileyko 2011: 42.

################ Material examined.

Syntypes NHMUK ex. Cuming collection from “Lao Mountains, Camboja” (2 shells; Fig. 47B). Specimens from km 31 from Xam Neua Town (Polytechnic School), Viengxay District, Houaphanh Province (Figs 47C, 58B).

################ Distribution.

Laos and Vietnam (Saurin 1953, Schileyko 2011).

############### Camaena leeana

Taxon classificationAnimaliaStylommatophoraCamaenidae

Thach, 2017

Camaena leeana Thach, 2017: 52, figs 693–696. Type locality: south of Thakhek, Khammouane Province, Central Laos.

################ Material examined.

Holotype FMNH 381984 (Fig. 47D).

################ Distribution.

Known only from the type locality in Laos (Thach 2017).

################ Remarks.

No material of this species was found, and only the type specimens were examined.

############### Camaena suprafusca

Taxon classificationAnimaliaStylommatophoraCamaenidae

Möllendorff, 1898

Camaena suprafusca Möllendorff, 1898: 71. Type locality: Boloven [Boloven Plateau, Paksong District, Champasak Province, Laos]. Richardson 1985: 78. Schileyko 2011: 43.
Camaena (Camaena) suprafusca : Zilch 1964: 245, pl. 6, fig. 4.

################ Material examined.

Lectotype SMF 8105 figured in Zilch (1964: pl. 6, fig. 4) and paralectotype SMF 8106 (1 shell). Specimens from Tad Moang waterfall, Khamkeut District, Bolikhamxay Province (Figs 47E, 58C).

################ Distribution.

Laos and possibly in Vietnam (Schileyko 2011).

############### Camaena vanbuensis

Taxon classificationAnimaliaStylommatophoraCamaenidae

Smith, 1896

Camaena vanbuensis Smith, 1896: 129, 130. Type locality: Vanbu, Tonkin [Van Ban District, Lao Cai Province, Vietnam]. Schileyko 2011: 43.
Camaena illustris vanbuensis : Richardson 1985: 74.

################ Material examined.

**Figure 48.**
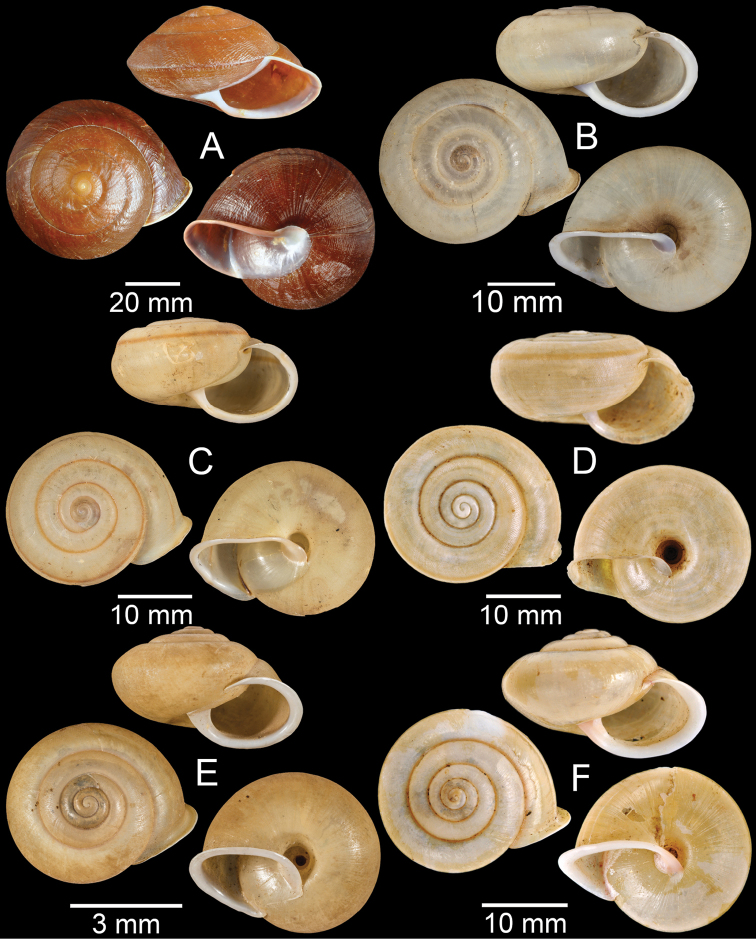
**A***Camaenavanbuensis*, CUMZ collection **B–D***Chloritisbalansai***B** syntype MNHN-IM-2000-2078 **C** syntype of “var. *cincta*” MNHN-IM-2000-2077 and **D** CUMZ collection **E, F***Chloritiscondoriana***E** syntype MNHN-IM-2000-1866 and **F** CUMZ collection.

Holotype NHMUK 1896.1.25.1 (Fig. 47F). Specimens from Ban Bo-Khoun village near Laos-China border, Boun Neua District, Phongsaly Province (Figs 48A, 58D).

################ Distribution.

Vietnam (Schileyko 2011).

############## *Chloritis* Beck, 1837

############### Chloritis balansai

Taxon classificationAnimaliaStylommatophoraCamaenidae

(Morlet, 1886)

Helix balansai Morlet, 1886a: 1. Type locality: Baie ďHalong et montagne de ľÉléphant [Ha Long Bay and Elephant Mountain, Quang Ninh Province, Vietnam]. Morlet 1887[1886]: 258, 270, 271, pl. 12, figs 4, 4a, b.
Helix (Chloritis) balansaivar.cincta Dautzenberg & Fischer, 1905: 90, 91, pl. 3, figs 5–9. Type locality: Ile Krieu, Archipel des Faï-Tsi-Long, Tonkin [Krieu Island, Ha Long Provincial, Quang Ninh Province, Vietnam]. Schileyko 2011: 45.
Chloritis (Trichochloritis) balansai : Gude 1906: 116.
Chloritis (Trichochloritis) balansaivar.cincta : Gude 1906: 116.
Chloritis balansai : Richardson 1985: 86.
Chloritis balansai cincta : Richardson 1985: 86.
Trachia balansai : Schileyko 2011: 45.

################ Material examined.

Syntype of “*balansai* Morlet, 1887” MNHN-IM-2000-2078 from “Tonkin” (1 shell; Fig. 48B). Syntypes of “var. *cincta*Dautzenberg and Fischer 1905” MNHN-IM-2000-2077 from “Ile Krieu” (2 shells; Fig. 48C). Specimens from Ngoy Town, Ngoy District, Luang Phrabang Province (Fig. 48D).

################ Distribution.

Laos and Vietnam (Schileyko 2011).

############### Chloritis caseus

Taxon classificationAnimaliaStylommatophoraCamaenidae

(Pfeiffer, 1860)

Helix caseus Pfeiffer, 1860a: 134. Type locality: Siam [Thailand].
Helix (Plectotropis) caseus : Fischer 1891: 26.
Chloritis (Trichochloritis) caseus : Gude 1906: 115.
Chloritis caseus : Richardson 1985: 88, 89.

################ Material examined.

**Figure 49.**
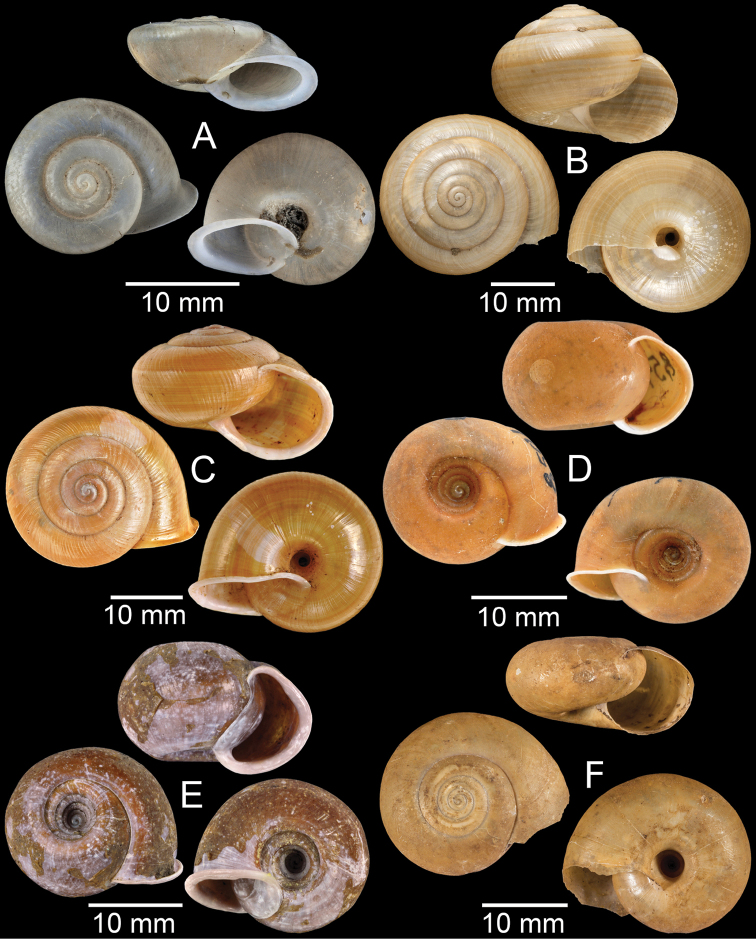
**A***Chloritiscaseus*, syntype NHMUK 20160333 **B, C***Chloritisdeliciosa***B** syntype NHMUK 20170017 and **C** CUMZ collection **D, E***Chloritisdiplochone***D** lectotype SMF 8594 and **E** CUMZ collection **F***Chloritisdurandi*, syntype MNHN-IM-2000-1882.

Syntypes NHMUK 20160333 from “Siam” (3 shells; Fig. 49A).

################ Distribution.

Cambodia, Laos and Thailand (Fischer 1891, Gude 1906).

################ Remarks.

No material of this species was found, and only the type specimens were examined.

############### Chloritis condoriana

Taxon classificationAnimaliaStylommatophoraCamaenidae

(Crosse & Fischer, 1863)

Helix condoriana Crosse & Fischer, 1863b: 351–353, pl. 14, fig. 1. Type locality: Poulo-Condor [Con Dao Islands, Ba Ria–Vung Tau Province, Vietnam].
Chloritis (Trichochloritis) condoriana : Gude 1906: 115.
Chloritis condoriana : Richardson 1985: 91.
Trichochloritis condoriana : Schileyko 2011: 47.

################ Material examined.

Syntype MNHN-IM-2000-1866 from “Ile de Poulo-Condor” (1 shell; Fig. 48E). Specimens from km 30, Laos-Vietnam border road, Yommalath District, Khammouan Province (Fig. 48F).

################ Distribution.

Vietnam (Schileyko 2011).

############### Chloritis deliciosa

Taxon classificationAnimaliaStylommatophoraCamaenidae

(Pfeiffer, 1863)

Helix deliciosa Pfeiffer, 1863a[1862]: 271, pl. 36, fig. 3. Type locality: Lao Mountains, Camboja [Cambodia or Laos].
Chloritis (Trichochloritis) deliciosa : Gude 1906: 116.
Chloritis deliciosa : Richardson 1985: 93.
Camaena deliciosa : Schileyko 2011: 42.

################ Material examined.

Syntypes NHMUK 20170017 from “Lao Mountains, Camboja” (3 shells; Fig. 49B). Specimens from Ban Bo-Khoun village, Laos-China border, Boun Neua District, Phongsaly Province (Figs 49C, 58E).

################ Distribution.

Laos, Vietnam and possibly in Cambodia (Pfeiffer 1863a, Schileyko 2011).

############### Chloritis diplochone

Taxon classificationAnimaliaStylommatophoraCamaenidae

Möllendorff, 1898

Chloritis diplochone Möllendorff, 1898: 72. Type locality: Boloven [Boloven Plateau, Paksong District, Champasak Province, Laos]. Richardson 1985: 94. Sutcharit and Panha 2010: 283, figs 1c, d, 2g–l, 3e–h.
Chloritis (Trichochloritis) diplochone : Gude 1906: 116.
Trichochloritis diplochone : Schileyko 2011: 47.

################ Material examined.

Lectotype SMF 8594 (Fig. 49D) and paralectotype SMF 8595 (1 shell). Specimens from km 30, Laos-Vietnam border road, Yommalath District, Khammouan Province (Fig. 49E).

################ Distribution.

Laos, Thailand and Vietnam (Sutcharit and Panha 2010, Schileyko 2011).

############### Chloritis durandi

Taxon classificationAnimaliaStylommatophoraCamaenidae

(Bavay & Dautzenberg, 1900)

Helix (Chloritis) durandi Bavay & Dautzenberg, 1900b: 111, 112. Type locality: Bac-Kan [Bac Kan Province, Vietnam]. Bavay and Dautzenberg 1900a: 441, pl. 11, figs 1–3.
Chloritis (Eustomopsis) durandi : Gude 1906: 112.
Chloritis durandi : Richardson 1985: 94, 95.
Trachia (?) durandi: Schileyko 2011: 45.

################ Material examined.

**Figure 50.**
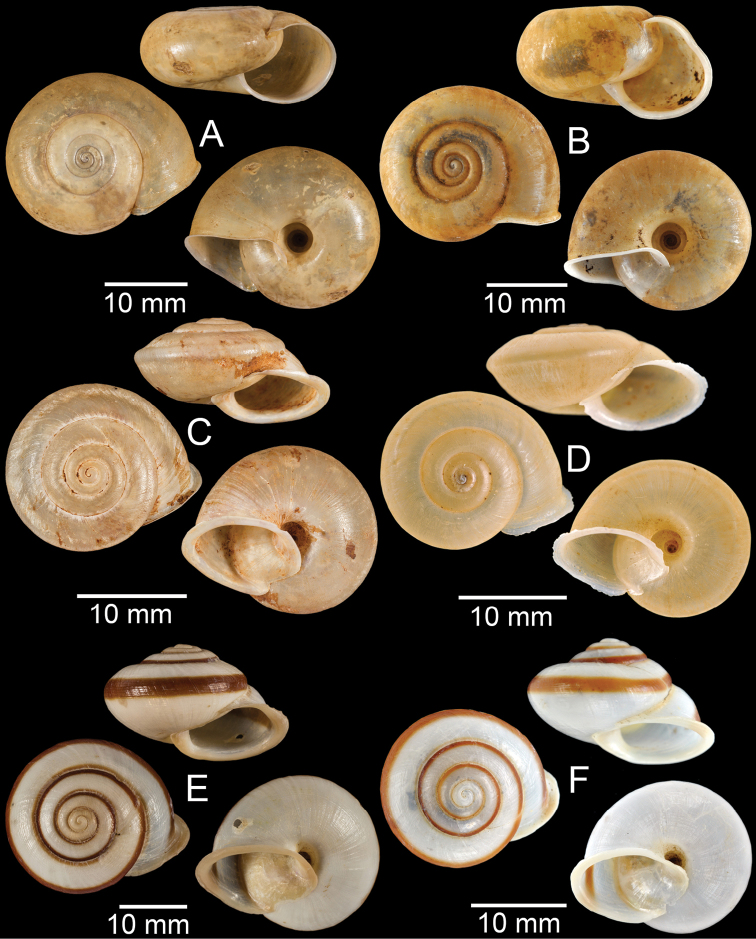
**A, B***Chloritisdurandi***A** syntype MNHN-IM-2000-1881 and **B** CUMZ collection **C, D***Chloritisfouresi***C** syntype MNHN-IM-2000-1888 and **D** CUMZ collection **E, F***Chloritiskhammouanensis* Inkhavilay and Panha, nom. nov. **E** holotype MNHN-IM-2000-33214 and **F** CUMZ collection.

Syntypes MNHN-IM-2000-1881 from “Bac-Kan, Tonkin” (1 shell; Fig. 50A) and MNHN-IM-2000-1882 From “Bac-Kan” (3 shells; Fig. 49F). Specimens from Ngoy Town, Ngoy District, Luang Phrabang Province (Figs 50B, 58F).

################ Distribution.

Vietnam (Schileyko 2011).

############### Chloritis fouresi

Taxon classificationAnimaliaStylommatophoraCamaenidae

(Morlet, 1886)

Helix fouresi Morlet, 1886b: 74. Type locality: Plateau de Stang-Trang, Cambodge [Steung Treng Province, Cambodia].
Fruticicola fouresi : Morlet 1889: 126, 176, 177, pl. 6, fig. 3.
Chloritis (Trichochloritis) fouresi : Gude 1906: 115.
Chloritis fouresi : Richardson 1985: 97.

################ Material examined.

Syntype MNHN-IM-2000-1888 from “Strung-Trang” (1 shell; Fig. 50C). Specimens from Nam Noua bridge, Viengxay District, Houaphanh Province (Fig. 50D).

################ Distribution.

Cambodia and Thailand (Morlet 1889).

############### Chloritis khammouanensis

Taxon classificationAnimaliaStylommatophoraCamaenidae

Inkhavilay & Panha nom. nov.

Megalacron huberi Thach, 2017: 53, 54, figs 741–743. [non Chloritishuberi Thach, 2016: 72, 73, figs 49, 407–410]. Type locality: Thakhek, Khammouane Province, South Central Laos.

################ Etymology.

The species name “*khammouanensis*” is from the type locality of the type specimens in Laos.

################ Material examined.

Holotype MNHN-IM-2000-33214 (Fig. 50E). Specimens from Tam Xang Cave, Thakhek District, Khammouan Province (Fig. 50F).

################ Distribution.

Known only from the type locality in Laos (Thach 2017).

################ Remarks.

This species was originally placed in the genus *Megalacron* Rensch, 1934. It seemed inappropriate since all the known members are distributed in the Bismarck and Solomon Islands. Moreover, the subfamily Papuninae where *Megalacron* belongs has a restricted distribution in New Guinea, Australia and Melanesia (Schileyko 2003b: 1605, 1606). With a medium shell size, depressed helicoid, expanded lip and rimate umbilicus, they are practically identical to the generic characters of *Chloritis* s.l., although this placement requires further anatomical study for confirmation.

By relocating *Megalacronhuberi* Thach, 2017 to the genus *Chloritis*, it becomes a junior secondary homonym of *Chloritishuberi* Thach, 2016 (see below). According to the ICZN guideline (ICZN 1999: Arts 57.3.1 and 60.3), the species name of a junior homonym has to be replaced, and so we propose *Chloritiskhammouanensis* Inkhavilay & Panha, nomen novum as the new replacement name.

############### Chloritis klausgrohi

Taxon classificationAnimaliaStylommatophoraCamaenidae

Thach & Huber, 2017

Chloritis klausgrohi Thach & Huber in Thach, 2017: 52, figs 729–732. Type locality: Laos.

################ Material examined.

**Figure 51.**
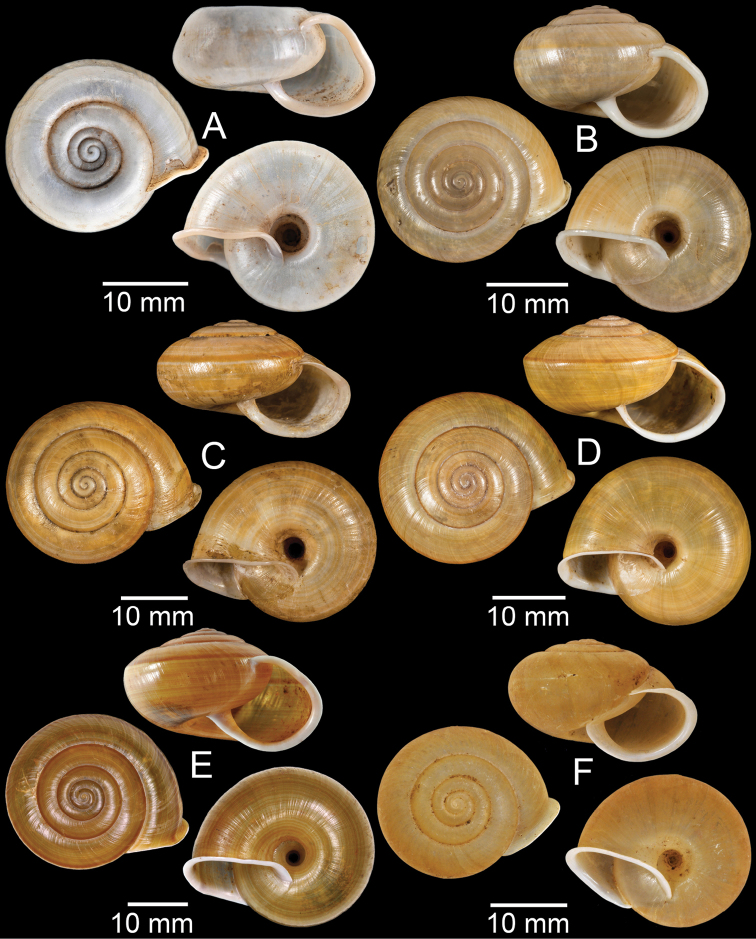
**A***Chloritisklausgrohi*, CUMZ collection **B***Chloritislemeslei*, syntype MNHN-IM-2000-1925 **C–E***Chloritismarimberti***C** syntype MNHN-IM-2000-1935 **D** syntype of “var. *carinata*” MNHN-IM-2000-2040 and **E** CUMZ collection **F***Chloritismicrotricha*, specimen NHMUK 1910.12.30.45.

Holotype RMNH 5006711 figured in Thach (2017: figs 729–731). Specimens from limestone outcrop at Tam Nang Rod Cave, Na-dan village, Yommalath District, Khammouan, Laos (Fig. 51A).

################ Distribution.

Known only from the type locality and Khammouan Provinces (Thach 2017).

############### Chloritis lemeslei

Taxon classificationAnimaliaStylommatophoraCamaenidae

(Morlet, 1891)

Helix (Chloritis) lemeslei Morlet, 1891a: 245, 249, 250, pl. 7, fig. 1. Type locality: Song-Ma [Song Ma District, Son La Province, Vietnam].
Chloritis (Trichochloritis) lemeslei : Gude 1906: 116.
Chloritis lemeslei : Richardson 1985: 101.
Camaena lemeslei : Schileyko 2011: 43.

################ Material examined.

Syntype MNHN-IM-2000-1925 from “Song-Ma” (1 shell; Fig. 51B).

################ Distribution.

Laos and Vietnam (Schileyko 2011).

################ Remarks.

No material of this species was found, and only the type specimen was examined.

############### Chloritis marimberti

Taxon classificationAnimaliaStylommatophoraCamaenidae

(Bavay & Dautzenberg, 1900)

Helix (Chloritis) marimberti Bavay & Dautzenberg, 1900b: 111. Type locality: Cho-Ra [Cho Ra Township, Ba Be District, Bac Kan Province, Vietnam]. Bavay and Dautzenberg 1900a: 440, 441, pl. 10, figs 4–6.
Chloritis (Trichochloritis) marimberti : Gude 1906: 116.
Helix (Chloritis) marimbertivar.carinata Bavay & Dautzenberg, 1909b: 180. Type locality: Muong Kong, Muong-Hum [Muong Khuong District; Muong Hum Town, Bat Xat District, Lao Cai Province, Vietnam].
Chloritis marimberti : Saurin 1953: 113. Richardson 1985: 103.
Chloritis marimberti carinata : Richardson 1985: 103.
Trachia marimberti carinata : Schileyko 2011: 45.
Trachia marimberti marimberti : Schileyko 2011: 45.

################ Material examined.

Syntype of “*marimberti* Bavay & Dautzenberg, 1900” MNHN-IM-2000-1935 from “Cho-Ra” (1 shell; Fig. 51C). Syntypes of “var. *carinata* Bavay & Dautzenberg, 1909” MNHN-IM-2000-2039 from “Muong Kong” (2 shells) and MNHN-IM-2000-2040 from “Muong-Hum” (2 shells; Fig. 51D). Specimens from Ban Nong Tang village, Phookood District, Xieng Khaung Province (Fig. 51E).

################ Distribution.

Laos and Vietnam (Saurin 1953, Schileyko 2011).

############### Chloritis microtricha

Taxon classificationAnimaliaStylommatophoraCamaenidae

Möllendorff, 1898

Chloritis microtricha Möllendorff, 1898: 71, 72. Type locality: Boloven [Boloven Plateau, Paksong District, Champasak Province, Laos]. Richardson 1985: 104.
Chloritis (Trichochloritis) microtricha : Gude 1906: 115. Zilch 1966: 304, pl. 9, fig. 23.
Trichochloritis microtricha : Schileyko 2011: 47.

################ Material examined.

Lectotype SMF 8540 figured in Zilch (1966: pl. 9, fig. 23) and paralectotypes SMF 8541 (3 shells). Specimen NHMUK 1910.12.30.45 from “Annam” (1 shell; Fig. 51F).

################ Distribution.

Laos and Vietnam (Schileyko 2011).

################ Remarks.

No material of this species was found, and only the old specimen was examined.

############### Chloritis nasuta

Taxon classificationAnimaliaStylommatophoraCamaenidae

(Bavay & Dautzenberg, 1908)

Helix (Chloritis) nasuta Bavay & Dautzenberg, 1908: 237, 238. Type locality: Muong-Hum [Muong Hum Commune, Bat Xat District, Lao Cai Province, Vietnam]. Bavay and Dautzenberg 1909b: 183, 184, pl. 6, figs 15–17.
Chloritis nasuta : Richardson 1985: 105.
Trachia nasuta : Schileyko 2011: 45.

################ Material examined.

**Figure 52.**
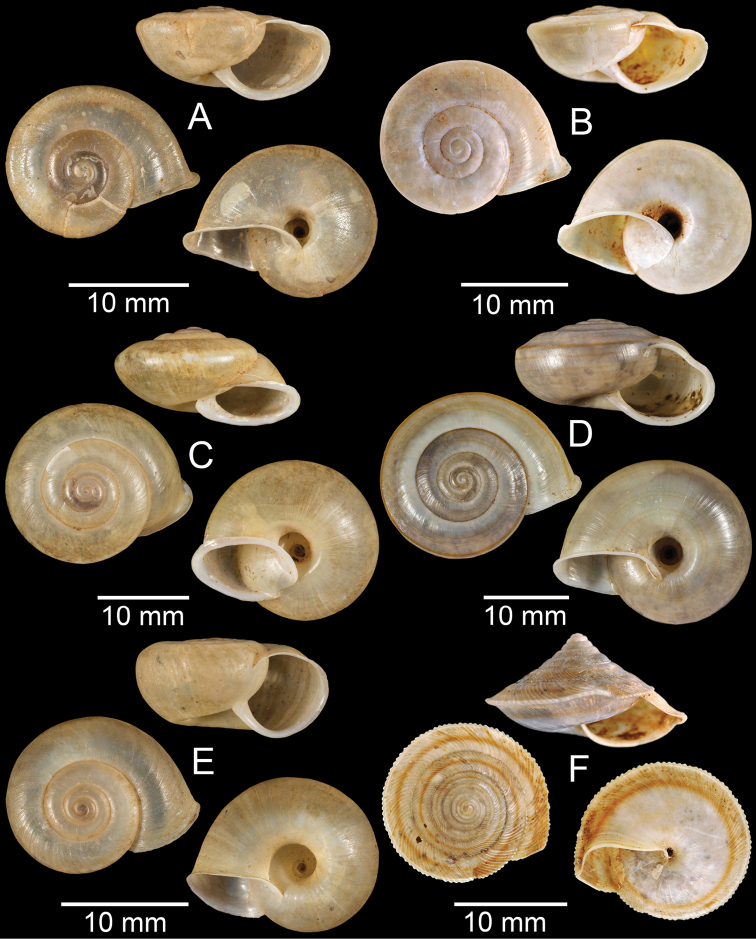
**A, B***Chloritisnasuta***A** syntype MNHN-IM-2000-2043 and **B** CUMZ collection **C***Chloritisnorodomiana*, syntype MNHN-IM-2000-1953 **D***Chloritisremoratrix*, syntype MNHN-IM-2000-1981 **E***Chloritistenella*, syntype MNHN-IM-2000-2045 **F***Ganesellahyperteleia*, CUMZ collection.

Syntype MNHN-IM-2000-2043 from “Muong-Hum” (1 shell; Fig. 52A). Specimens from Ngoy Town, Ngoy District, Luang Phrabang Province (Fig. 52B).

################ Distribution.

Vietnam (Schileyko 2011).

############### Chloritis norodomiana

Taxon classificationAnimaliaStylommatophoraCamaenidae

(Morlet, 1883)

Helix norodomiana Morlet, 1883: 106, 107, pl. 4, figs 3, 3a, b. Type locality: Khamchay [Cambodia].
Chloritis (Trichochloritis) norodomiana : Gude 1906: 116.
Chloritis norodomiana : Richardson 1985: 105, 106.
Trachia norodomiana : Schileyko 2011: 45.

################ Material examined.

Syntype MNHN-IM-2000-1953 from “Kamchay” (1 shell; Fig. 52C).

################ Distribution.

Cambodia, Laos, probably in Thailand (Chiang Mai Province) and Vietnam (Schileyko 2011).

################ Remarks.

No material of this species was found, and only the type specimen was examined.

############### Chloritis remoratrix

Taxon classificationAnimaliaStylommatophoraCamaenidae

(Morlet, 1893)

Helix (Chloritis) remoratrix Morlet, 1893[1892]: 317, 318, pl. 6, figs 3, 3a, b. Type locality: Route de Bassac à Siempang, sur la rive gauche de Mékong, dans le Laos [road from Champasak (Laos) to Siem Pang District, Stung Treng Province (Cambodia), on the left bank of Mekong River in Laos].
Chloritis remoratrix : Fischer and Dautzenberg 1904: 401. Richardson 1985: 110.
Chloritis (Trichochloritis) remoratrix : Gude 1906: 116.

################ Material examined.

Syntype MNHN-IM-2000-1981 from “Route de Bassac à Siempang, Laos” (1 shell; Fig. 52D).

################ Distribution.

Laos, probably in Cambodia, and Vietnam (Fischer and Dautzenberg 1904).

################ Remarks.

No material of this species was found, and only the type specimen was examined.

############### Chloritis tenella

Taxon classificationAnimaliaStylommatophoraCamaenidae

(Pfeiffer, 1862)

Helix tenella Pfeiffer, 1862: 42, pl. 5, figs 6, 7. Type locality: Siam [Thailand].
Chloritis (Trichochloritis) tenella : Gude 1906: 116.
Chloritis tenella : Richardson 1985: 113.
Aegista (?) tenella: Schileyko 2011: 38.

################ Material examined.

Syntype MNHN-IM-2000-2045 from “Siam” (1 shell; Fig. 52E).

################ Distribution.

Cambodia, Laos, Thailand and Vietnam (Schileyko 2011).

################ Remarks.

No material of this species was found, and only the type specimen was examined.

############## *Ganesella* Blanford, 1863

############### Ganesella hyperteleia

Taxon classificationAnimaliaStylommatophoraCamaenidae

(Morlet, 1892)

Helix (Plectotropis) hyperteleia Morlet, 1892b: 82, 83. Type locality: Kham-Keute, dans le Laos [around Kham Kheuth District, Bolikhamxay Province, Laos]. Morlet 1893[1892]: 316, 317, pl. 6, figs 2, 2a, b.
Ganesella hyperteleia : Richardson 1985: 137.
Plectotropis hyperteleia : Schileyko 2011: 39.

################ Material examined.

Specimens from Tam Mungkorn Cave, Khamkeut District, Bolikhamxay Province (Fig. 52F).

################ Distribution.

Laos and probably in Vietnam (Schileyko 2011).

############### Ganesella leptopomopsis

Taxon classificationAnimaliaStylommatophoraCamaenidae

(Dautzenberg & Fischer, 1908)

Satsuma leptopomopsis Dautzenberg & Fischer, 1908: 180, 181, pl. 4, figs 17–19. Type locality: Lung-Phoi, près That-Khé [Lung Po Town, Tra Linh District, Cao Bang Province; That Khe Town, Trang Dinh District, Lang Son Province, Vietnam].
Ganesella leptopomopsis : Richardson 1985: 139. Schileyko 2011: 48.

################ Material examined.

**Figure 53.**
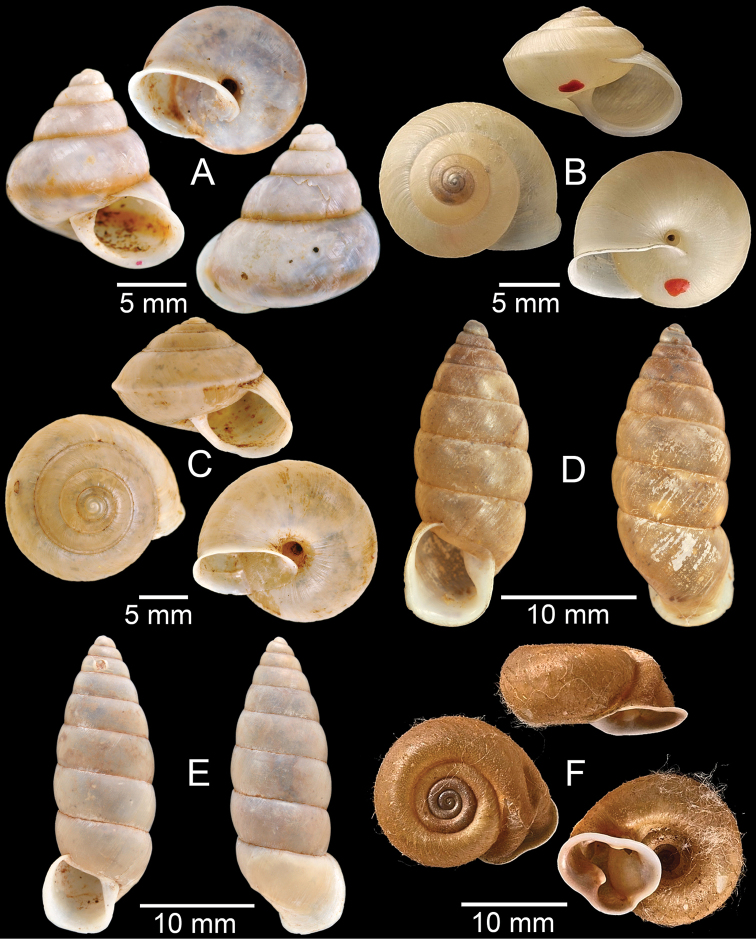
**A***Ganesellaleptopomopsis*, CUMZ collection **B, C***Ganesellarostrella***B** syntype NHMUK 20130217 and **C** CUMZ collection **D, E***Giardiasiamensis***D** specimen NHMUK ex. Cuming collection and **E** CUMZ collection **F***Moellendorffiahorrida*, syntype NHMUK ex. Cuming collection.

Specimens from Tam Xang Cave, Ban Nam Kha village, Kham District, Xieng Khaung Province (Fig. 53A).

################ Distribution.

Vietnam (Schileyko 2011).

############### Ganesella rostrella

Taxon classificationAnimaliaStylommatophoraCamaenidae

(Pfeiffer, 1863)

Helix rostrella Pfeiffer, 1863a[1862]: 270. Type locality: Lao Mountains, Camboja [Cambodia or Laos]. Pfeiffer 1868b: 379, pl. 88, figs 1–3.
Bradybaena (Torobaena) rostrella : Richardson 1983: 46.
Bradybaena (?) rostrella: Schileyko 2011: 40.

################ Material examined.

Syntypes NHMUK 20130217 from “Lao Mountains, Camboja” (3 shells; Fig. 53B). Specimens from Par-Houak limestone, Ban Vieng Swarng village, Vieng Phouka District, Luang Namtha Province (Fig. 53C).

################ Distribution.

Laos, Vietnam, and possibly in Cambodia (Pfeiffer 1863a, Schileyko 2011).

############## *Giardia* Ancey, 1906

############### Giardia siamensis

Taxon classificationAnimaliaStylommatophoraCamaenidae

(Redfield, 1853)

Bulimus siamensis Redfield, 1853: 15, 16. Type locality: Siam [Thailand]. Pfeiffer 1861b: 170, pl. 46, figs 3, 4.
Pseudobuliminus (Giardia) siamensis : Solem 1966: 104.
Pseudobuliminus (Girardius [sic]) siamensis: Richardson 1983: 94, 95.
Giardia siamensis : Schileyko 2003b: 1519, fig. 1960. Schileyko 2011: 46.

################ Material examined.

Specimens NHMUK ex. Cuming collection from “Siam” (3 shells; Fig. 53D). Specimens from Ban Phone village, Lamam District, Sekong Province (Figs 53E, 58G).

################ Distribution.

Cambodia, Thailand and Vietnam (Solem 1966, Schileyko 2011).

############## *Moellendorffia* Ancey, 1887

############### Moellendorffia horrida

Taxon classificationAnimaliaStylommatophoraCamaenidae

(Pfeiffer, 1863)

Helix horrida Pfeiffer, 1863a[1862]: 272, pl. 36, fig. 15. Type locality: Lao Mountains, Camboja [Cambodia or Laos]. Pfeiffer 1868b: 399, 400, pl. 92, figs 17–19.
Helicodonta (Moellendorffia) horrida : Fischer and Dautzenberg 1904: 404
Moellendorffia horrida : Saurin 1953: 113. Richardson 1985: 185.

################ Material examined.

**Figure 54.**
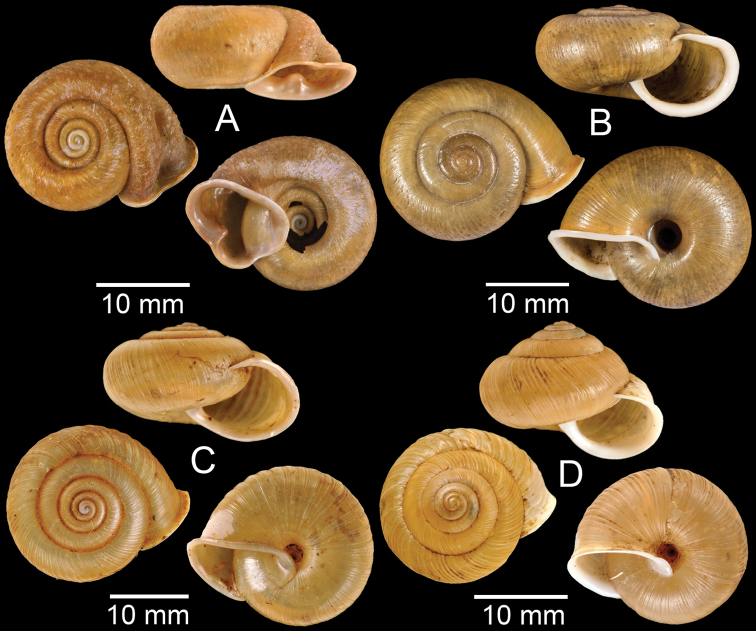
**A***Moellendorffiahorrida*, CUMZ collection **B–D***Trachiapseudomiara***B** syntype MNHN-IM-2000-31774 and **C, D** CUMZ collection.

Syntypes NHMUK ex. Cuming collection from “Lao Mountains, Camboja: (3 shells; Fig. 53F). Specimens from Ngoi Town, Ngoy District, Luang Phrabang Province (Figs 54A, 58H).

################ Distribution.

Laos (Fischer and Dautzenberg 1904, Saurin 1953).

############## *Trachia* Martens, 1860

############### Trachia pseudomiara

Taxon classificationAnimaliaStylommatophoraCamaenidae

(Bavay & Dautzenberg, 1909)

Helix (Chloritis) pseudomiara Bavay & Dautzenberg, 1909d[1908]: 236. Type locality: Nat-Son, Binh-Lu, Muong-Hum [Nat Son Commune, Kim Boi District, Hoa Binh Province; Binh Lieu District, Quang Ninh Province; Muong Hum Commune, Bat Xat District, Lao Cai Province, Vietnam]. Bavay and Dautzenberg 1909b: 181, 182, pl. 6, figs 5–8.
Helix (Chloritis) pseudomiaravar.minor Bavay & Dautzenberg, 1909d[1908]: 236. Type locality: Phong-Tho [Phong Tho District, Lai Chau Province, Vietnam]. Bavay and Dautzenberg 1909b: 181, 182. Schileyko 2011: 45.
Chloritis pseudomaria [sic]: Richardson 1985: 108.
Chloritis pseudomaria [sic] minor: Richardson 1985: 108.
Trachia pseudomiara : Schileyko 2011: 45.

################ Material examined.

Syntype of “*pseudomiara* Bavay & Dautzenberg, 1909” MNHN-IM-2000-31774 from “Nat-Son” (1 shell; Fig. 54B). Specimens from Wat Pathammawath Sen Oudom, Lak 20, Khamkeut District, Bolikhamxay Province (Fig. 54C, D).

################ Distribution.

Vietnam (Schileyko 2011).

**Figure 55.**
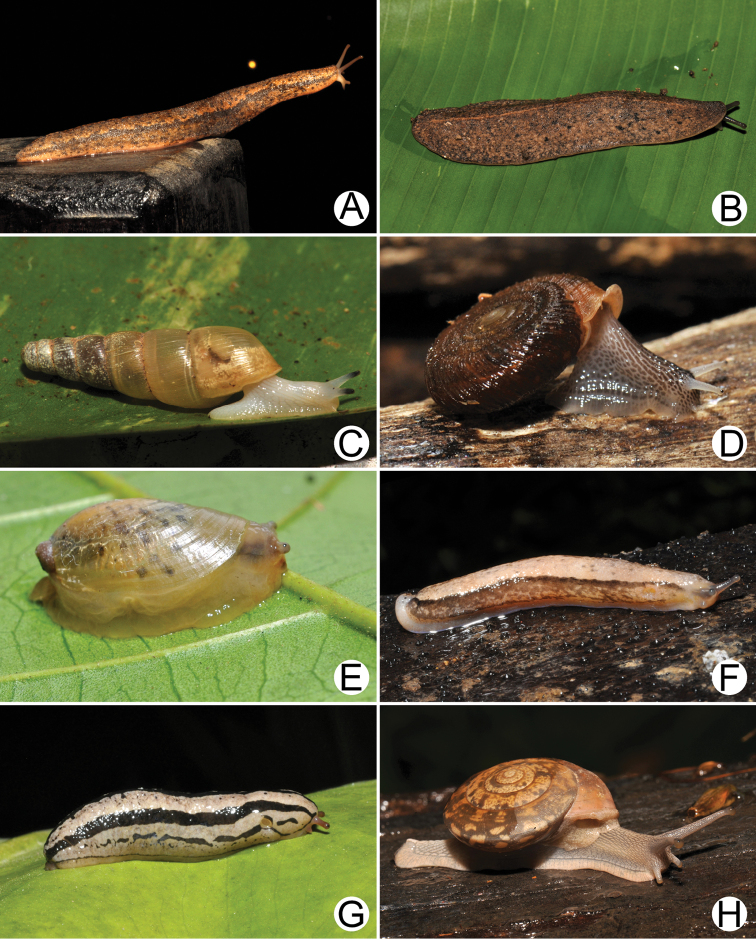
Living snails of **A***Atoposlaidlawi***B***Valigunasiamensis***C***Prosopeasexcellens***D***Gudeodiscus* sp. **E***Succenia* sp. **F***Meghimatiumbilineatum***G***Meghimatiumpictum***H***Quantulaweinkauffiana*. All not to scale.

**Figure 56.**
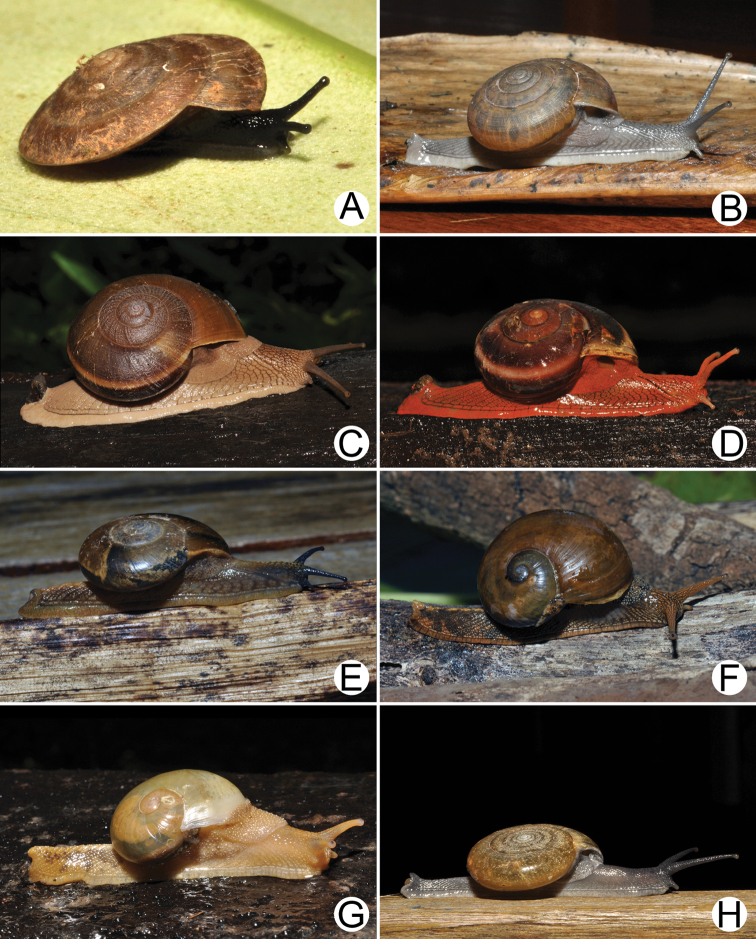
Living snails of **A***Trochomorphapaviei***B***Cryptozonasiamensis***C***Hemiplectadistincta***D***Hemiplectapluto***E***Macrochlamyscallojuncta***F***Megausteniamalefica***G***Megausteniasiamensis***H***Sarikadespecta*. All not to scale.

**Figure 57.**
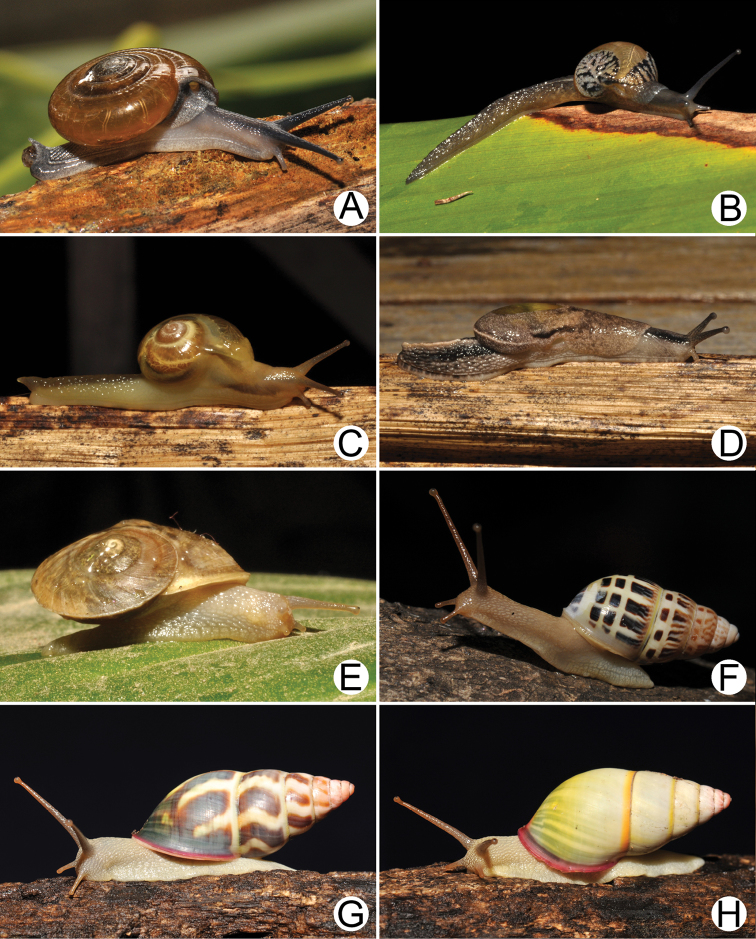
Living snails of **A***Sarikaresplendens***B***Cryptosemelus* sp. **C***Durgellalibas***D***Parmarionmartensi***E***Aegistaemma***F***Amphidromusareolatus***G, H***Amphidromusfuscolabris*. All not to scale.

**Figure 58.**
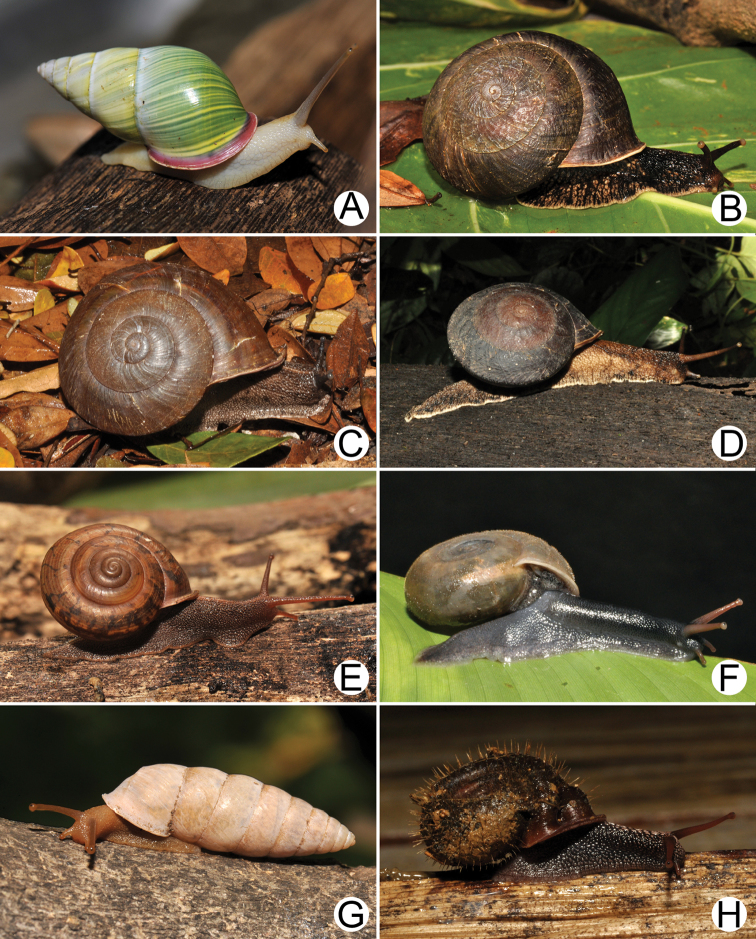
Living snails of **A***Amphidromusroseolabiatus***B***Camaenaillustris***C***Camaenasuprafusca***D***Camaenavanbuensis***E***Chloritisdeliciosa***F***Chloritisdurandi***G***Giardiasiamensis***H***Moellendorffiahorrida*. All not to scale.

##### Uncertain records in Laos

###### Family Streptaxidae Gray, 1860

####### *Discartemon* Pfeiffer, 1856

######## Discartemon discus

Taxon classificationAnimaliaStylommatophoraStreptaxidae

(Pfeiffer, 1853)

Streptaxis discus Pfeiffer, 1853a[1851]: 252. Type locality: unknown.
Streptaxis (Discartemon) paradiscus Möllendorff, 1900: 117. Type locality: Phucson bei Touranne, Annam [Da Nang Province, Vietnam].
Discartemon paradiscus : Schileyko 2011: 22, 23.
Discartemon discus : Siriboon et al. 2014: 53–55, figs 4a–c, 11a–c, 22a. Inkhavilay et al. 2016a: 26.

######### Material examined.

**Figure 59.**
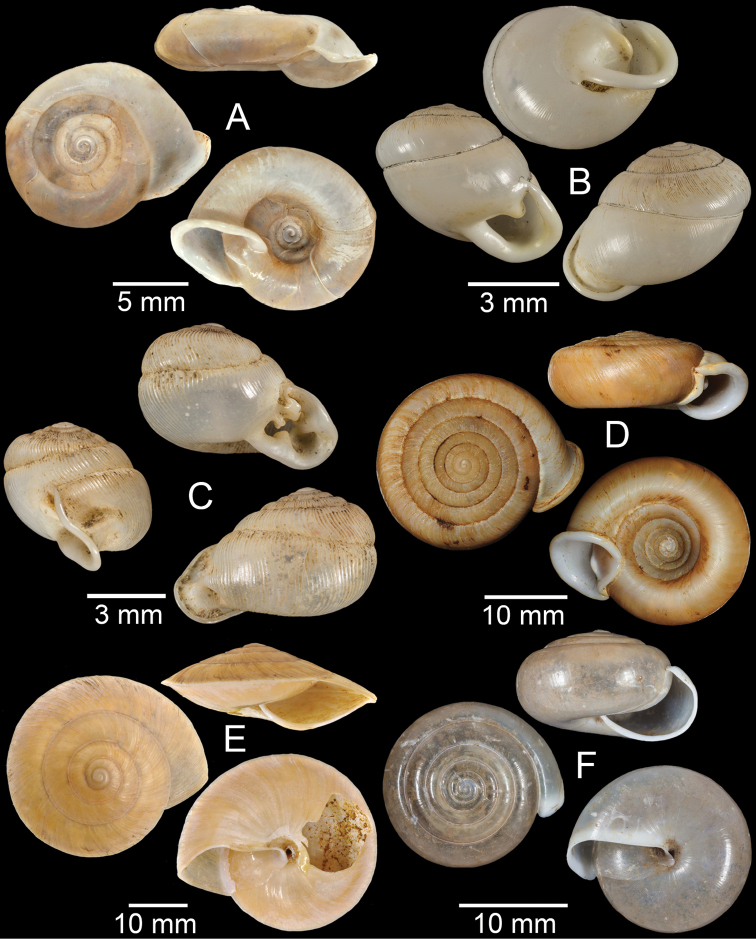
**A***Discartemondiscus*, holotype NHMUK 20130684 **B***Haploptychiusfischeri*, lectotype MNHN-IM-2000-30873 **C***Perrottetiadaedalea*, lectotype MNHN-IM-2000-30871 **D***Halongellaschlumbergeri*, syntype MNHN-IM-2000-24582 **E***Hemiplectadura*, syntype NHMUK ex. Cuming collection **F***Sarikadugasti*, syntype MNHN-IM-2000-27884. Photo: B. Páll-Gergely (**D**).

Lectotype NHMUK 20130684 (Fig. 59A).

######### Distribution.

Vietnam and Laos (Schileyko 2011).

######### Remarks.

No material of this species was found, and only the type specimen was examined. As Schileyko (2011) did not give the exact locality and reference to the record of this species in Laos, and all other previous records were from “Annam” [the old name for Vietnam] (Siriboon et al. 2014a), the occurrence of this species in Laos is questionable.

######## Haploptychius fischeri

Taxon classificationAnimaliaStylommatophoraStreptaxidae

(Morlet, 1886)

Streptaxis fischeri Morlet, 1886a: 2. Type locality: baie ďHalong et montagne de ľÉléphant [Ha Long Bay and Elephant Mountain, Quang Ninh Province, Vietnam]. Morlet 1887[1886]: 259, 274, 275, pl. 12, figs 1, 1a.
Haploptychius fischeri : Schileyko 2011: 25. Inkhavilay et al. 2016a: 35, 36, fig. 3g.

######### Material examined.

Lectotype MNHN-IM-2000-30873 (Fig. 59B).

######### Distribution.

Laos and Vietnam (Schileyko 2011).

######### Remarks.

No material of this species was found, and only the type specimen was examined. Schileyko (2011) listed this species as occurring in Laos from the Elephant Mountains. However, this locality is possibly more likely to be the Elephant Islet of Ha Long Bay, Quang Ninh Province, Vietnam. Thus, the occurrence of this species in Laos is questionable.

######## Perrottetia daedalea

Taxon classificationAnimaliaStylommatophoraStreptaxidae

(Bavay & Dautzenberg, 1909)

Streptaxis daedaleus Bavay & Dautzenberg, 1909d[1908]: 230. Type locality: Pac-Kha [Pa Kha in Long Luong Commune, Van Ho District, Son La Province, Vietnam]. Bavay and Dautzenberg 1909b: 164, 165, pl. 4, figs 1–4.
Streptaxis daedaleus var. major Bavay and Dautzenberg 1909d[1908]: 231. Type locality: Pac-Kha [Pa Kha in Long Luong Commune, Van Ho District, Son La Province, Vietnam].
Perrottetia daedalea : Schileyko 2011: 23.
Perrottetia daedaleus [sic]: Inkhavilay et al. 2016a: 38–40, fig. 5c.

######### Material examined.

Syntype of “var. *major* Bavay & Dautzenberg, 1909” MNHN-IM-2000-30871 from “Pac-Kha” (1 shell; Fig. 59C).

######### Distribution.

Laos and possibly in Vietnam (Schileyko 2011).

######### Remarks.

No material of this species was found, and only the type specimen was examined. Schileyko (2011) attributed the type locality (Pac-Kha) to Laos. However, this locality is more likeky to be Pa Kha in Long Luong Commune, Van Ho District, Son La Province, Vietnam. Thus, the occurrence of this species in Laos is questionable.

###### Family Plectopylidae Möllendorff, 1898

####### *Halongella* Páll-Gergely, 2015

######## Halongella schlumbergeri

Taxon classificationAnimaliaStylommatophoraPlectopylidae

(Morlet, 1886)

Helix (Plectopylis) schlumbergeri Morlet, 1886a: 1, 2. Type locality: baie ďHalone et montagne de ľÉléphant, Tonkin [Ha Long Bay and Elephant Mountain, Quang Ninh Province, Vietnam]. Morlet 1887[1886]: 259, 272–274, pl. 12, figs 2, 2a–c.
Endoplon schlumbergeri : Schileyko 2011: 8.
Halongella schlumbergeri : Páll-Gergely et al. 2015b: 71–86, figs 6a–d, 9m, n, 14h–n, 26, 29a, b, h, 30g–i, 33a–g, 36d–f, 45b.

######### Material examined.

Syntypes MNHN-IM-2000-24582 (2 shells; Fig. 59D).

######### Distribution.

Laos and Vietnam (Schileyko 2011, Páll-Gergely et al. 2015b).

######### Remarks.

Although Schileyko (2011) listed this species as occurring in Laos from the Elephant Mountains, the recent revision by Páll-Gergely et al. (2015b) did not list any specimens from Laotian area. In addition, Elephant Mountain is more likely to be the Elephant Islet of Ha Long Bay, Quang Ninh Province, Vietnam. Thus, the occurrence of this species in Laos is questionable and no material of this species was found from this survey to clarify the distribution range of this species.

###### Family Vertiginidae Fitzinger, 1833

####### Gyliotrachela crossei

Taxon classificationAnimaliaStylommatophoraVertiginidae

(Morlet, 1886)

Hypselostoma crossei Morlet, 1886a: 2, 3. Morlet 1887[1886]: 259, 275, 276, pl. 12, figs 5, 5a–c. Type locality: Montagne de ľÉléphant, Tonkin [Elephant Mountain of Ha Long Bay, Quang Ninh Province, Vietnam].
Gyliotrachela crossei crossei : Schileyko 2011: 3.

######## Distribution.

Cambodia, Laos and Vietnam (Schileyko 2011).

######## Remarks.

No material of this species was found and the type specimen could not be traced. This species was figured in Morlet (1887: pl. 12, fig. 5; see Fig. 17I). Schileyko (2011) listed this species as occurring in Laos from the Elephant Mountains. However, this locality is possibly more likely to be the Elephant Islet of Ha Long Bay, Quang Ninh Province, Vietnam. Thus, the occurrence of this species in Laos is questionable.

###### Family Trochomorphidae Möllendorff, 1890

####### Trochomorpha sapeca

Taxon classificationAnimaliaStylommatophoraTrochomorphidae

(Heude, 1890)

Helix sapeca Heude, 1890: 143, pl. 38, fig. 13. Type locality: in monte conico juxta Tay-ninh Cochinchine [probably refers to the hills in Tay Ninh Province, Vietnam].
Geotrochus sapeca : Schileyko 2011: 36.

######## Distribution.

Laos and Vietnam (Schileyko 2011).

######## Remarks.

No material of this species was found. Schileyko (2011) listed this species as occurring in Laos from the Elephant Mountains. However, this locality is possibly more likely to be the Elephant Islet of Ha Long Bay, Quang Ninh Province, Vietnam. Thus, the occurrence of this species in Laos is questionable.

###### Family Ariophantidae Godwin-Austen, 1888

####### Hemiplecta dura

Taxon classificationAnimaliaStylommatophoraAriophantidae

(Pfeiffer, 1864)

Helix dura Pfeiffer, 1864[1863]: 524. Type locality: Waigiou Island [Waigeo Island, Raja Ampat Regency, West Papua Province, Indonesia]. Pfeiffer 1868a: 129.
Hemiplecta dura : Wallace 1865: 406. Gude 1903a: 7. Gude 1903b: 96.
Nanina (Hemiplecta) dura : Tapparone Canefri 1883: 201.
Ariophanta (Hemiplecta) dura : Fischer 1891: 22.

######## Material examined.

Syntypes NHMUK ex. Cuming collection from “Waigiou Island” (2 shells; Fig. 59E).

######## Distribution.

West Papua Province, Indonesia (Wallace 1865, Tapparone Canefri 1883, Gude 1903b) and Laos (Pfeiffer 1868a, Fischer 1891, Gude 1903a).

######## Remarks.

No material of this species was found in this study and no specimen with a precise locality from Indochina has been recorded so far. Only the type specimens were examined, and the type specimen is illustrated herein for the first time. The species was originally described from West Papua, Indonesia (Pfeiffer 1864). Later, Pfeiffer (1868a) recorded this species from “Lao Mountains, Cambojae” based on H. Mouhot specimens. Since then, subsequent records followed the previous literature without specimens from Indochina. Thus, we place this species as an uncertain record in Laos.

####### *Microparmarion* Simroth, 1893

######## Microparmarion andamanica

Taxon classificationAnimaliaStylommatophoraAriophantidae

Collinge, 1901

Microparmarion andamanica Collinge, 1901b: 17, 18, pl. 1, figs 7–10. Type locality: North Andaman [error]. Schileyko 2011: 35.
Microparmarion annamica Collinge, 1901a: 120. Type locality: Mekong Valley, Annam [correct type locality].

######### Distribution.

Laos and probably in Vietnam (Schileyko 2011).

######### Remarks.

The correct type locality of this species should be “Mekong Valley, Annam” (see Collinge 1901a). No material of this species was found, and the species was figured in Collinge (1901b: pl. 1, figs 7, 8, see Fig. 17J). Schileyko (2011) attributed the type locality (Mekong Valley) to Laos, whereas Collinge (1901b) also mentioned “Annam” which is the old name for Vietnam. Thus, the occurrence of this species in Laos is questionable.

######## Sarika dugasti

Taxon classificationAnimaliaStylommatophoraAriophantidae

(Morlet, 1891)

Macrochlamys dugasti Morlet, 1891b: 25, 26. Type locality: forêts des bords du Ménam-Pinh, Laos occidental [forest edges of Ping River, Thailand].
Ariophanta (Macrochlamys) dugasti : Morlet 1891a: 231, 239, 240, pl. 5, figs 1, 1a.
Sarika dugasti : Schileyko 2011: 34.

######### Material examined.

Syntype MNHN-IM-2000-27884 from “Forêts des bords du Ménam-Pinh, Laos occidental” (1 shell; Fig. 59F).

######### Distribution.

Laos, Nepal, Thailand and probably in Vietnam (Schileyko 2011).

######### Remarks.

No material of this species was found, and only the type specimen was examined. The type locality is mentioned as “Ménam-Pinh, Laos occidental” where Schileyko (2011) attributed this locality to Laos. However, this locality is probably the Ping River which flows from Chiang Mai to Lamphun, Tak, Kampang Phet and Nakhon Sawan Provinces in Thailand. This species has been collected from several localities in Chiang Mai, Lamphun and Tak Provinces, so these are likely to be a more precise type locality. Thus, the occurrence of this species in Laos is questionable.

###### Family Camaenidae Pilsbry, 1895

####### Subfamily Camaeninae Pilsbry, 1895

######## Amphidromus dautzenbergi

Taxon classificationAnimaliaStylommatophoraCamaenidae

Fulton, 1899

Amphidromus dautzenbergi Fulton, 1899: 303, fig. 3. Type locality: Tonkin [north of Vietnam]. Laidlaw and Solem 1961: 527, 528. Sutcharit et al. 2015: 67, fig. 6g. Schileyko 2011: 50.

######### Material examined.

**Figure 60.**
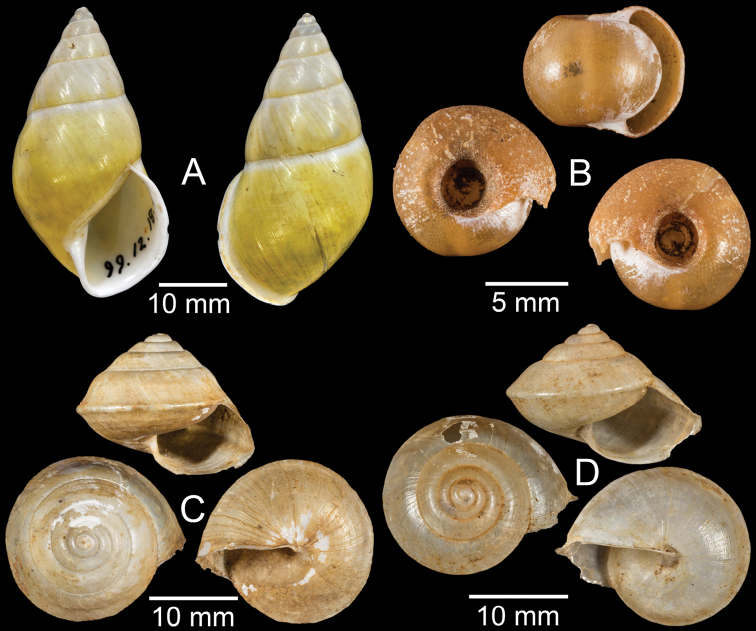
**A***Amphidromusdautzenbergi*, holotype NHMUK 1899.12.18.38 **B***Chloritishuberi*, paratype MNHN-IM-2014-6068 **C, D***Globotrochusonestera***C** syntype MNHN-IM-2000-32456 and **D** specimen MNHN-IM-2000-2073.

Holotype NHMUK 1899.12.18.38 (Fig. 60A).

######### Distribution.

Laos and Vietnam (Schileyko 2011).

######### Remarks.

No material of this species was found, and only the type specimen was examined. Schileyko (2011) listed this species as occurring in Laos from Ban Lao. However, this locality is more likely to be Ban Lao in Muong Bum Commune, Thuan Chau District, Son La Province, Vietnam. Thus, the occurrence of this species in Laos is questionable.

######## Camaena hainanensis

Taxon classificationAnimaliaStylommatophoraCamaenidae

(Adams, 1870)

Helix (Camaena) hainanensis Adams, 1870: 8, pl. 1, fig. 15. Type locality: Hainan [Hainan Province, China].
Camaena hainanensis : Richardson 1985: 73.
Camaena hainanensis hainanensis : Schileyko 2011: 42.

######### Distribution.

Hainan Island, Laos and Vietnam (Schileyko 2011).

######### Remarks.

Schileyko (2011) listed this species as occurring in Laos from the Elephant Mountains. However, this locality is more likely to be the Elephant Islet of Ha Long Bay, Quang Ninh Province, Vietnam. In addition, Páll-Gergely (pers. comm.) suggested this species does not occur in Laos. Thus, the occurrence of this species in Laos is questionable.

######## Chloritis huberi

Taxon classificationAnimaliaStylommatophoraCamaenidae

Thach, 2016

Chloritis huberi Thach, 2016: 72, 73, figs 49, 407–410. Type locality: Khanh Vinh, Kanh Hao Province, (Central Vietnam).

######### Material examined.

Holotype ANSP 466247, paratype MNHN-IM-2014-6068 (1 shell; Fig. 60B).

######### Distribution.

The type locality and probably in Da Lat, Lam Dong Province, Vietnam (Thach 2016).

######### Remarks.

The relatively small size, and a simple apertural lip without constriction all probably indicate that the type specimens are juveniles. In the original description, Thach (2016) compared this new species with *Chloritisbifoveata* (Benson, 1856b: 251) from Myanmar instead of the proximal species, *Chloritisdiplochone* from Southern Laos (Sutcharit and Panha 2010). However, a biconcave shell shape with a narrow umbilicus and spire side, and the last whorl superimposed on the penultimate whorl are likely to be unusual distinguishing characters of the species. As this species is similar to *Chloritisdiplochone* (Fig. 48D, E), we surmise that this species could possibly occur in Laos. Therefore, we follow the original identification and wait for additional adult specimens to clarify the species status and its distribution range.

####### *Globotrochus* Haas, 1935

######## Globotrochus onestera

Taxon classificationAnimaliaStylommatophoraCamaenidae

(Mabille, 1887)

Helix onestera Mabille, 1887a: 3. Type locality: Tonkin [Northern Vietnam]. Mabille 1887b: 89, 90, pl. 2, figs 4, 5.
Ganesella onestera : Richardson 1985: 140.
Globotrochus onestera : Schileyko 2011: 46.

######### Material examined.

Syntypes MNHN-IM-2000-32456 from “Tonkin” (2 shells; Fig. 60C). Specimen MNHN-IM-2000-2073 from “Tonkin” (1 shell; Fig. 60D).

######### Distribution.

Laos and Vietnam (Schileyko 2011).

######### Remarks.

No material of this species was found, and only the type specimens were examined. Schileyko (2011) listed this species as occurring in Laos from the Elephant Mountains. However, this locality is possibly more likely to be the Elephant Islet of Ha Long Bay, Quang Ninh Province, Vietnam. Thus, the occurrence of this species in Laos is questionable.
